# CatSper mediates not only chemotactic behavior but also the motility of ascidian sperm

**DOI:** 10.3389/fcell.2023.1136537

**Published:** 2023-11-02

**Authors:** Taiga Kijima, Daisuke Kurokawa, Yasunori Sasakura, Michio Ogasawara, Satoe Aratake, Kaoru Yoshida, Manabu Yoshida

**Affiliations:** ^1^ Misaki Marine Biological Station, School of Science, The University of Tokyo, Miura, Kanagawa, Japan; ^2^ Shimoda Marine Research Center, University of Tsukuba, Shimoda, Japan; ^3^ Department of Biology, Graduate School of Science, Chiba University, Chiba, Japan; ^4^ Faculty of Biomedical Engineering, Toin University of Yokohama, Yokohama, Kanagawa, Japan

**Keywords:** sperm motility, chemotaxis, Ca^2+^ channel, CatSper, spermatogenesis, CRISPR/Cas9, *Ciona intestinalis*

## Abstract

**Introduction:** Sperm motility, including chemotactic behavior, is regulated by changes in the intracellular Ca^2+^ concentration, and the sperm-specific Ca^2+^ channel CatSper has been shown to play an important role in the regulation of intracellular Ca^2+^. In particular, in mammals, CatSper is the only functional Ca^2+^ channel in the sperm, and mice deficient in the genes comprising the pore region of the Ca^2+^ channel are infertile due to the inhibition of sperm hyperactivation. CatSper is also thought to be involved in sea urchin chemotaxis. In contrast, in ascidian *Ciona intestinalis*, SAAF, a sperm attractant, interacts with Ca^2+^/ATPase, a Ca^2+^ pump. Although the existence of *CatSper* genes has been reported, it is not clear whether CatSper is a functional Ca^2+^ channel in sperm.

**Results:** We showed that CatSper is present in the sperm flagella of *C. intestinalis* as in mammalian species, although a small level of gene expression was found in other tissues. The spermatozoa of *CatSper3* KO animals were significantly less motile, and some motile sperms did not show any chemotactic behavior. These results suggest that CatSper plays an important role in ascidians and mammals, and is involved in spermatogenesis and basic motility mechanisms.

## 1 Introduction

The regulation of sperm motility, including chemotactic behavior, is a well-organized single-cell behavior in complex field environments, and Ca^2+^ is indispensable for controlling sperm behavior (Yoshida and Yoshida, 2011; Yoshida and Yoshida, 2018). In particular, Ca^2+^ regulates flagellar beating patterns (Brokaw et al., 1974; Brokaw, 1979) and the direction of sperm swimming (Shiba et al., 2008). The concentration of intracellular Ca^2+^ ([Ca^2+^]_i_) is regulated by Ca^2+^ influx via Ca^2+^ channels and Ca^2+^ efflux driven by Ca^2+^ exchangers or Ca^2+^ pumps; thus, both Ca^2+^ channels and Ca^2+^ pumps play a role in sperm function.

In ascidians, an increase in [Ca^2+^]_i_ is also involved in sperm motility and chemotactic behavior toward the egg (Yoshida et al., 1994; Yoshida et al., 2003; Shiba et al., 2008). The sulfate-conjugated hydroxysteroid, SAAF, acts as a sperm activator and attractant in ascidians (Yoshida et al., 2002; Oishi et al., 2004). Spermatozoa with chemotactic behavior undergo transient increases in [Ca^2+^]_i_ in the flagellum (Ca^2+^ bursts) when the concentration of SAAF decreases to a maximum, forming an asymmetric flagellar waveform of the sperm and inducing a series of sperm movements, including turning and straight swimming (Yoshida et al., 2003; Shiba et al., 2008; Miyashiro et al., 2015). The regulation of [Ca^2+^]_i_ is mainly carried out by SAAF, which controls the Ca^2+^ efflux, and SAAF binds to and activates Ca^2+^/ATPase on the sperm plasma membrane (PMCA), thus causing Ca^2+^ efflux (Yoshida et al., 2018). However, the molecules involved in Ca^2+^ influx remain unknown, although the involvement of store-operated Ca^2+^ channels has been suggested pharmacologically (Yoshida et al., 2003).

Among many Ca^2+^-regulating systems, the Ca^2+^ channel on the plasma membrane is an important player in Ca^2+^ influx. In mammalian sperm, CatSper, which is a sperm-specific Ca^2+^ channel, plays a crucial role in the regulation of sperm function. CatSper is homologous to a voltage-gated Ca^2+^ channel (Ren et al., 2001) and consists of four pore-forming subunits (CatSper 1, 2, 3, and 4) (Qi et al., 2007; Lishko et al., 2012; Bystroff, 2018) and several auxiliary subunits (CatSper β, *γ*, *δ*, *ε*, *ζ*, *η*, and EFCAB9) (Liu et al., 2007; Chung et al., 2011; Chung et al., 2017; Hwang et al., 2019). CatSper is located in the sperm flagella and mediates hyperactivation (Quill et al., 2001; Ren et al., 2001; Carlson et al., 2003; Qi et al., 2007). Furthermore, CatSper seems to be the only functional Ca^2+^ channel in mammalian sperm (Zeng et al., 2013); thus, CatSper is implicated in not only mediating hyperactivation but also flagellar beating and swimming patterns.

In contrast to its crucial role in mammals, CatSper is absent in many animals, including birds, amphibians, and teleostean fishes (Cai and Clapham, 2008). Furthermore, the expression and function of CatSper proteins in animals other than mammals have been poorly investigated, although CatSper appears to regulate sperm chemotaxis in sea urchins (Seifert et al., 2015). In the genome database of the ascidian *Ciona intestinalis*, all CatSper pore-forming isoforms (CatSper 1–4) are present, similar to mammals (Cai and Clapham, 2008).

Because CatSper is a complex protein with many subunits, it has not been successfully expressed functionally in cultured cells (Chung et al., 2017). There is only one study in which the voltage sensor domain of CatSper3 was expressed in HEK293T cells and *Xenopus* oocytes (Arima et al., 2018). Therefore, functional analyses of CatSper have been performed using spermatozoa from genetically deficient animals, and until now, most studies have been conducted using mice. Thus, we examined the role of CatSper in ascidians to identify the effectors of Ca^2+^ influx during sperm chemotaxis. Interestingly, CatSper expression in the ascidian was not restricted to the sperm flagellum but was also observed in other cells. Furthermore, we developed the *CatSper3*-deficient ascidian using the CRISPR/Cas9 system and found that CatSper is an indispensable Ca^2+^ channel in ascidian sperm. However, it plays a role not only in sperm chemotaxis but also in fundamental sperm motility.

## 2 Materials and methods

### 2.1 Materials

The ascidian *C. intestinalis* (type A; also called *Ciona robusta*) was obtained from the National BioResource Project for *Ciona* (http://marinebio.nbrp.jp/). Ascidian sperm were obtained as previously described (Yoshida et al., 1993). Artificial seawater (ASW) contained 462 mM NaCl, 9 mM KCl, 10 mM CaCl_2_, 48 mM MgCl_2_, and 10 mM HEPES-NaOH (pH 8.2). SAAF and its derivatives were synthesized, as described previously (Oishi et al., 2003; Oishi et al., 2004). Juveniles were developed by artificial insemination using mature eggs and sperm obtained from the dissected gonoducts. Whole-mount juveniles and dissected adult specimens were prepared for *in situ* hybridization, as described by Nakayama and Ogasawara (2017).

### 2.2 RNA isolation and cDNA synthesis

Tissue samples were collected from the testes, ovaries, muscles, gills, and hearts of adult individuals (*n* = 5) of *C. intestinalis.* Whole juveniles 1 week (100 individuals), 3 weeks (30 individuals), and 4 weeks (15 individuals) after fertilization were used in one batch (*n* = 3). Total RNA was extracted from each specimen using an RNeasy Plant Mini Kit (QIAGEN, Hilden, Germany), according to the manufacturer’s instructions. The total RNA concentration was determined using the Qubit RNA HS Assay (Thermo Fisher Scientific, Waltham, MA, United States). RNA quality was analyzed using a Bioanalyzer 2000 system (Agilent, Santa Clara, CA, United States) with an RNA 6000 Nano Kit (Agilent). The results of the RNA quality check are shown in Supplementary Material (Supplementary Figure S2). According to the MIQE guidelines, only RNA samples with an RIN value of eight or higher were used for qPCR analysis. Total RNA (150 ng) was reverse-transcribed to cDNA using oligo d(T)_20_ primers or random hexamers and the SuperScript IV First-Strand Synthesis System Kit (Thermo Fisher Scientific), according to the manufacturer’s instructions. Individually synthesized cDNA using oligo d(T)_20_ primers and random hexamers was combined and used for quantitative real-time polymerase chain reaction (qRT-PCR) analyses.

### 2.3 Quantitative real-time PCR (qRT-PCR)

Equal amounts of cDNA from each tissue sample were used for the qRT-PCR analysis. Quantitative polymerase chain reactions were performed using the PowerUp SYBR Green Master Mix (Thermo Fisher Scientific), according to the manufacturer’s instructions. Briefly, 1 μL of cDNA was used in a final volume of 20 μL, containing 10 μL of PowerUp SYBR Green Master Mix and 500 nM of each primer for *CatSper1*, *CatSper2*, *CatSper3*, *CatSper4*, and *GAPDH*. Predicted *CatSper* and *GAPDH* genes were identified from the genome database of *C. intestinalis* type A (*C. robusta*) (Ghost: http://ghost.zool.kyoto-u.ac.jp/default_ht.html) (Satou et al., 2022). The gene IDs are as follows: *CatSper1*, KH.C10.377; *CatSper2*, KH.S391.7; *CatSper3*, KH. C2.323; and *CatSper4*, KH.C2.993. Real-time PCR was performed using a StepOnePlus Real-Time PCR System (Thermo Fisher Scientific), and 40 PCR cycles were performed. Prior to the cycling and DNA denaturation steps, polymerase activation was achieved at 95°C for 20 s. The cycling conditions consisted of denaturation at 95°C for 3 s, annealing and extension at 60°C for 30 s, and plate reading at 50°C for 2 min. A melt-curve analysis of 15 s at 95°C and 1 min at 60°C, increasing 0.2°C per second to 95°C, was performed at the end. qPCR data were analyzed using StepOne software for calculating Cp values, and the standard curve method was used to determine the expression of *CatSper1*, *CatSper2*, *CatSper3*, and *CatSper4* relative to *GAPDH*, which was used as a housekeeping gene. The primers used for qPCR are listed in Supplementary Table S1.

### 2.4 Whole-mount *in situ* hybridization

Digoxigenin-labeled antisense RNA probes for the *Ciona* CatSper genes were synthesized from T7-RNA polymerase promoter-attached amplified cDNA, and the probes were purified by centrifugal ultrafiltration, as previously described by Ogasawara et al. (2001). Whole-mount *in situ* hybridization (WISH) of the *Ciona* specimens was performed using “*InSitu* Chip,” as described by Ogasawara et al. (2006). Gene expression signals were visualized using nitroblue tetrazolium/5-bromo-4-chloro-3-indolylphosphate (NBT/BCIP) solutions using a standard method (Thermo Fisher Scientific). Whole-mount and sectioned specimens of *Ciona* were observed using an SZX12 stereomicroscope (Olympus, Tokyo, Japan).

### 2.5 Gene knockdown by the CRISPR/Cas9 system

The CRISPR/Cas9 method (Cong et al., 2013; Hwang et al., 2013) was used for genome editing in *C. intestinalis*, as previously described by Sasaki et al. (2014). We designed target sequences of gRNA that are specific for *CatSper3* as 5′-CGA​AAG​CGA​AGT​TTT​TAA​TGG​TCT-3′ at exon 5 using ZiFiT (http://zifit.partners.org/ZiFiT) (Sander et al., 2007; Sander et al., 2010). The target DNAs were inserted into the BsaI site of pDR274 (Addgene; Hwang et al., 2013) as vectors for the *in vitro* transcription of gRNAs. The Cas9 gene, a gift from George Church (hCas9) (Addgene plasmid #41815; http://n2t.net/addgene:41815; RRID:Addgene_41815) (Mali et al., 2013), was sub-cloned into pCS2+ as a vector for *in vitro* transcription of mRNAs. Cas9 gRNAs and mRNAs were synthesized using the MEGAshortscript T7 and MEGAshortscript SP6 Transcription Kits (Thermo Fisher Scientific), respectively.

We injected approximately 30–100 pL of the RNA solution containing 400 ng/μL gRNA, 800 ng/μL Cas9 mRNA, and 10% phenol red into unfertilized eggs, and reared embryos and animals in aquariums.

We extracted DNA from juveniles or muscles of adults and performed PCR to check the genome editing efficiency using the heteroduplex mobility assay (Ota et al., 2013). The primers used for PCR are 5′-TTG​ACT​GCT​CTC​ATA​GAC​ACG​ATG-3′ and 5′-TTA​CCG​TAA​CGA​AGC​TGA​AGA​GC-3′. After purifying the PCR bands by electrophoresis, the products were sub-cloned into pGEM-T Easy (Promega, Madison, WI, United States) for sequencing analysis using a Genetic Analyzer (ABI 3130, Applied Biosystems).

### 2.6 Histochemistry

The testes were dissected and fixed in Bouin’s fixative consisting of 71.4% saturated picric acid, 23.8% formalin, and 4.7% acetic acid for 2 h at room temperature. The tissues were embedded in paraffin and cut into 4-μm-thick sections. Some sections were stained with hematoxylin and eosin for morphological observation and made into permanent specimens.

### 2.7 Western blotting

Samples were directly solubilized in the NuPAGE LDS sample buffer (Thermo Fisher Scientific). The solubilized samples were treated with benzonase (Novagen, Billerica, MA, United States). Proteins were separated on a NuPAGE SDS-PAGE Gel System using 4%–12% Bis–Tris gels (Thermo Fisher Scientific) and transferred onto PVDF membranes. The anti-CatSper3 antibody (ab197924; Abcam, Cambridge, UK), horseradish peroxidase (HRP)-conjugated anti-rabbit IgG (GE Healthcare), and ECL Prime (GE Healthcare) were used to detect CatSper. Luminescence was imaged using a C-DiGit Blot Scanner (LI-COR Biosciences, Lincoln, NE, United States). Images were processed using Adobe Photoshop and Adobe Illustrator (Adobe, San Jose, CA, United States).

### 2.8 Indirect immunofluorescence

Sperm suspension was put onto a coverslip, fixed with 4% paraformaldehyde for 10 min, and washed twice with ASW for 5 min. To visualize, the sperm suspension was incubated with 1 µM MitoBright IM Red (Dojindo, Kumamoto, Japan) for 30 min before fixation. The fixed sperms were permeabilized with 0.1% NP-40 for 15 min, blocked with 1% BSA for 1 h, and incubated with 0.6 μg/mL anti-α tubulin monochronal antibody (#236-10501: Invitrogen) or 4.3 μg/mL anti-CatSper3 antibody (ab197924, Abcam) with 1% BSA in PBS for 1 h. Sperms were washed three times with PBS and incubated with x1/2000 Alexa 488-conjugated anti-rabbit IgG (ab150077, Abcam) or x1/2000 Alexa 568-conjugated anti-mouse IgG (ab175473, Abcam) for 1 h. After washing twice with PBS, the stained sperms were mounted with ProLong Glass Antifade Mountant with NucBlue Stain (Thermo Fisher Scientific) and observed by confocal laser-scanning microscopy (FV3000; Olympus, Tokyo, Japan). For control experiments, the samples were stained only with Alexa 488-conjugated anti-rabbit IgG.

### 2.9 Analysis of sperm motility and chemotaxis

Sperm chemotaxis was examined, as previously described by Shiba et al. (2008). Briefly, semen was diluted 10^4^–10^5^ times in ASW with 1 mM theophylline (Sigma-Aldrich, Tokyo, Japan) to activate motility (Yoshida et al., 1994). The activated sperm suspension was placed in the observation chamber, and the sperm movement around the micropipette tip containing SAAF was recorded. The position of the sperm head was analyzed using Bohboh (BohbohSoft, Tokyo, Japan) (Shiba et al., 2002). The motility and chemotactic activity parameters (trajectory, distance between the capillary tip and sperm, and linear-equation-based chemotaxis index (LECI)) were calculated, as previously described by Yoshida et al. (2002). LECI is an index of the slope of a linear approximation of the time course of the distance between the sperm head and the attraction source, which indicates the average speed at which the sperms approach the attraction source (Yoshida et al., 2002).

### 2.10 Statistical analysis

**FIGURE 1:**
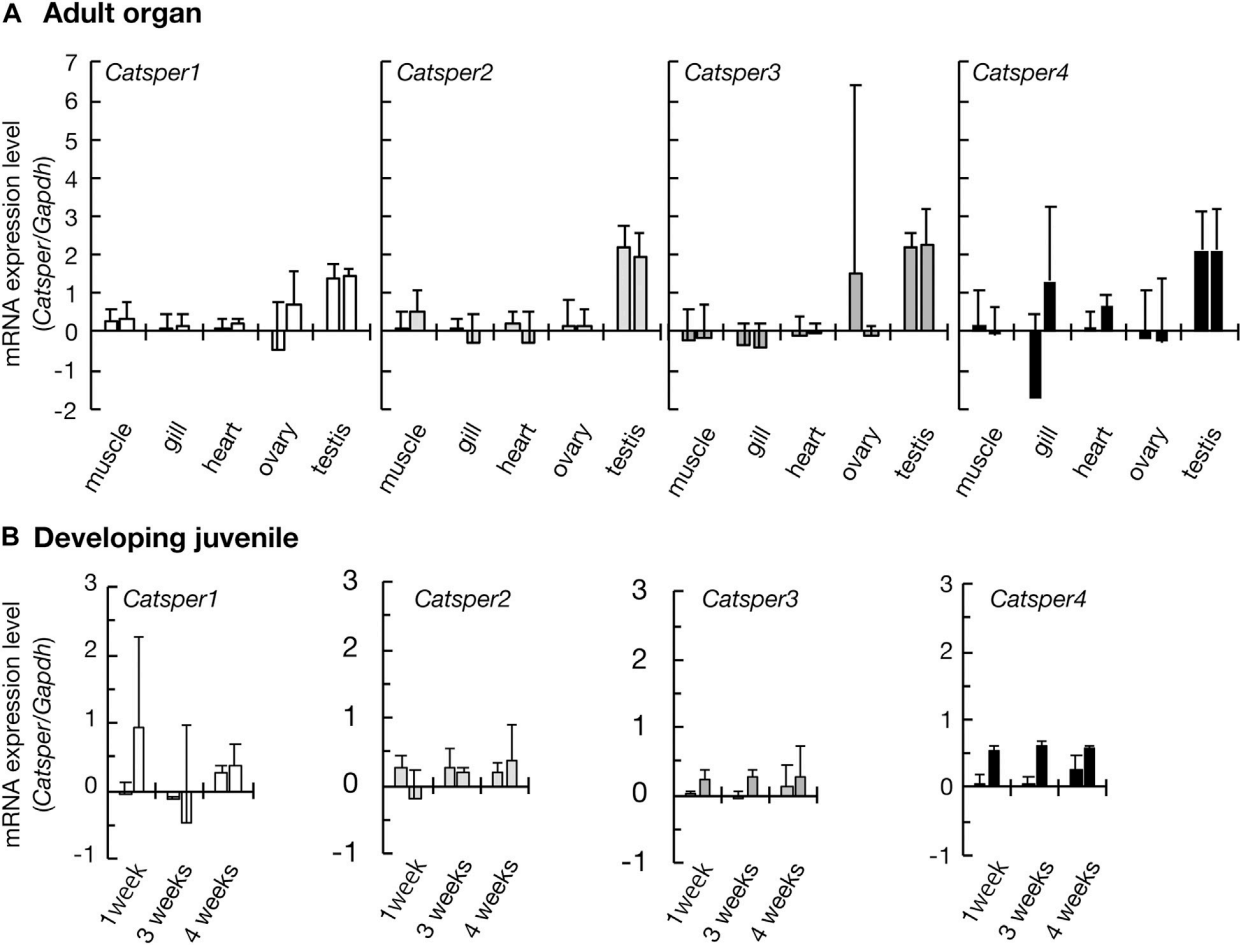
Expression of *CatSper* genes in the ascidian *C*. *intestinalis*. Gene expression was examined by quantitative real-time PCR. **(A)** Gene expression in the adult organs. **(B)** Gene expression in the developing juveniles. Two different primer pairs were used for the quantification of each gene (see Supplementary Table S1). Gene expression levels were normalized to the household gene glyceraldehyde-3-phosphate dehydrogenase (*GAPDH*). Values are expressed as mean ± S.D. (*n* = 3–5). There is a significant difference between the testis and some other organs (Tukey’s HSD-test: *p < 0.05). All raw data on qPCR are shown in Supplementary Figure S3 in Supplementary Material.

All experiments were repeated at least three times for different specimens. Data are expressed as mean ± SD. Statistical significance in Figure 1 was calculated using Tukey’s test; *p* < 0.05 was considered significant. Statistical significance in Figures 6, 8 was calculated using the Student’s t-test; *p* < 0.01 was considered significant. Tukey’s analyses were performed using EZR (Saitama Medical Center, Jichi Medical University, Saitama, Japan), a graphical user interface for R (The R Foundation for Statistical Computing, Vienna, Austria).

## 3 Results

### 3.1 CatSper expression in the ascidian

**FIGURE 2:**
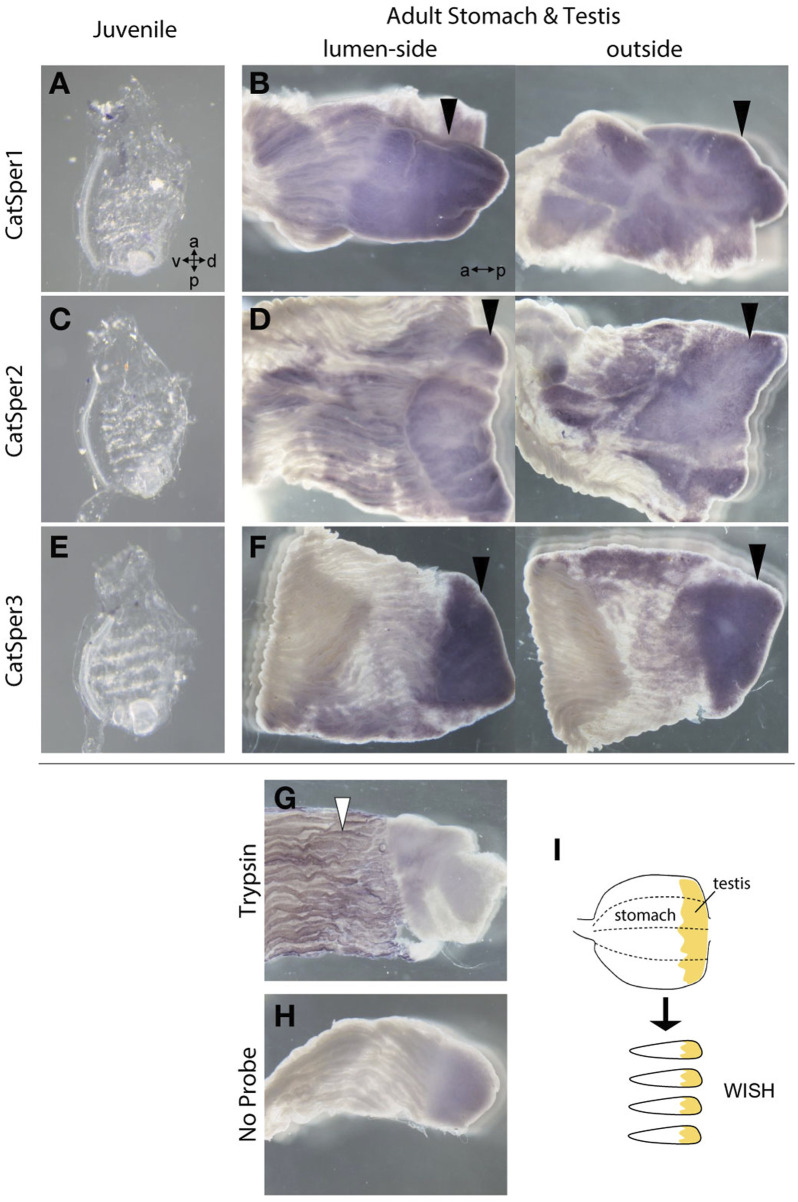
The WISH method was used to analyze the expression of *CatSper1*, *CatSper2*, and *CatSper3* in the ascidian juveniles and adult stomach with testis. **(A, B)** Typical expression of *CatSper1* in the juvenile **(A)** and the adult stomach with testis **(B)**. **(C, D)** Typical expression of *CatSper2* in the juvenile **(C)** and the adult stomach with testis **(D)**. **(E, F)** Typical expression of *CatSper3* in the juvenile **(E)** and the adult stomach with testis **(F)**. None of the *CatSper* subtypes were expressed in juveniles but only in adult testes (black arrowhead). a, anterior side; p, posterior side; v, ventral side; d, dorsal side. **(G, H)** Control of WISH: the probe of *trypsin-like* (XP_002126930.1) for positive control **(G)** and no probe for negative control **(H)**. Positive signal is denoted by a white arrowhead. **(I)** Schematic drawing of stomach preparations.

Initially, we examined CatSper expression in the ascidian *C. intestinalis* using RT-PCR. In adults, all *CatSper* subtypes (*CatSper1*, *2*, *3*, and *4*) were mainly expressed in the testis, but weak expression was also observed in other organs: the ovary, heart, gill, and muscle (Figure 1). Although the expression levels of *CatSper* genes in organs other than the testes varied because quantities of mRNA were adjusted to the heart in the qPCR process, which has the lowest amount of obtained total RNA, in most cases, the expression levels in the testes were still significantly higher than those in the other organs. In contrast, for the *CatSper4* gene, no significant differences in expression levels between the heart and testes were observed for either of the two primer sets used. The expression of *CatSper* genes in organs other than the testes was also confirmed using semi-quantitative RT-PCR (Supplementary Figure S1). When the expression of *CatSper* was examined in developmental juveniles, all *CatSper* genes were expressed in juveniles aged 1–4 weeks, although the expression was weak (Figure 1, Supplementary Figure S1). The 1- and 2.5-week-old juveniles were still immature, and their testes had not developed at this stage (Okada and Yamamoto, 1999). Thus, the expression of *CatSper* in ascidians is not restricted to the testis. On the other hand, using WISH, the expression of *CatSper* was observed in adult testis but not detected in juveniles (Figure 2). These results indicate that ascidian *CatSper* genes are mainly expressed in the testis, similar to those of mammalian species, but are weakly expressed in places other than the testis.

### 3.2 CatSper3 was localized around the ascidian sperm flagella

**FIGURE 3:**
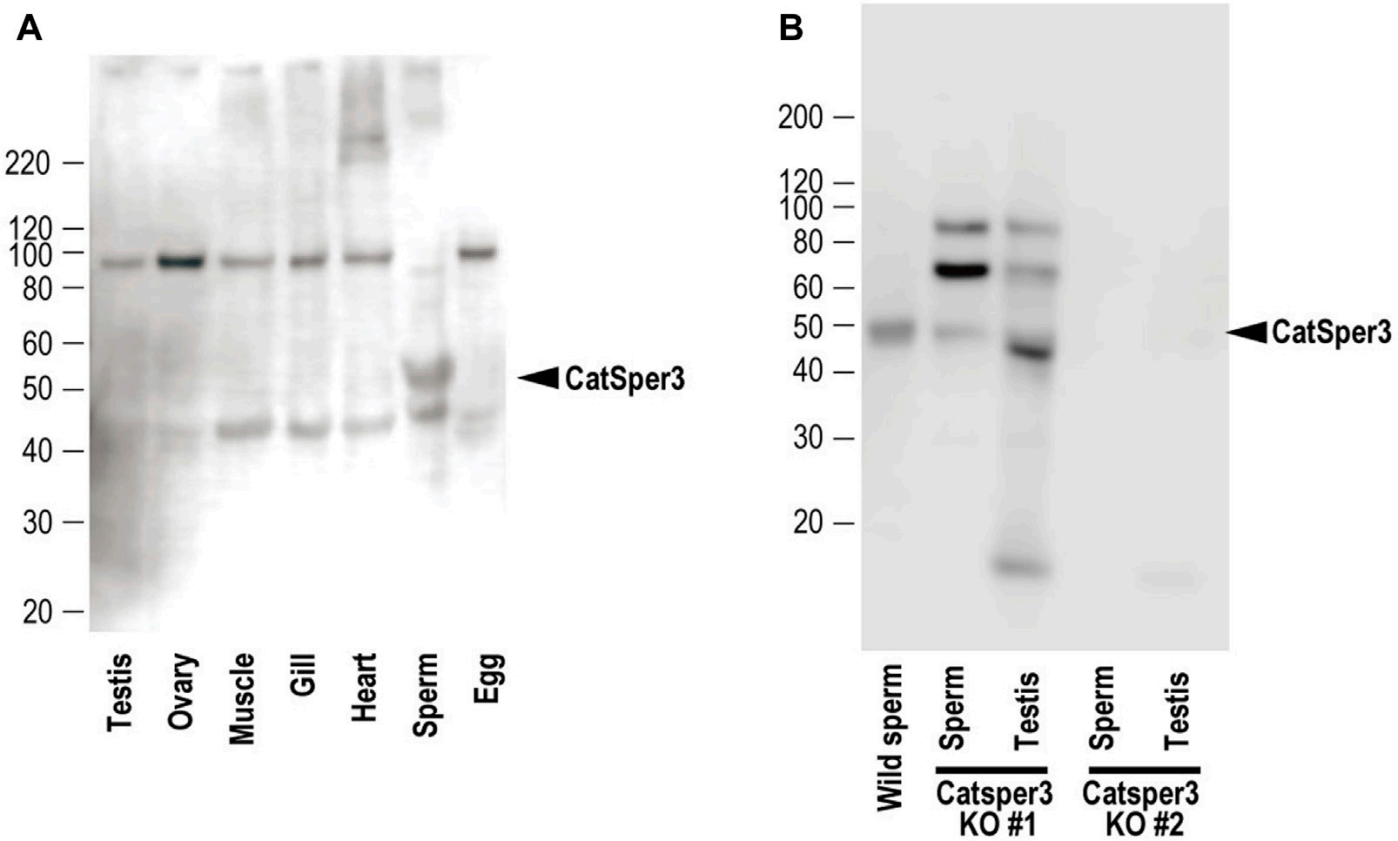
Representative results of Western blotting with the anti-CatSper3 antibody of **(A)** each tissue of mature adults, and **(B)** sperm and testis from the two *CatSper3* KO animals. The ascidian CatSper3 protein showed a 50-kDa band (arrowhead), whereas the predicted size from the deduced amino acid sequence is 49809 Da. Control data (without the anti-CatSper3 antibody) on the Western blotting are shown in Supplementary Figure S4 in Supplementary Material.

**FIGURE 4:**
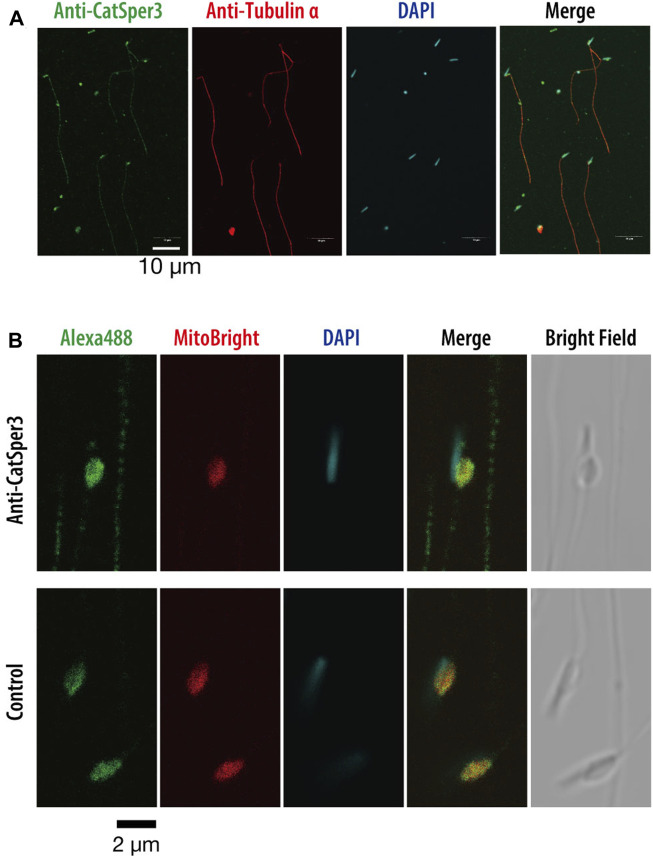
Indirect immunofluorescence assay with the anti-CatSper3 antibody in the ascidian sperm. **(A)** Nuclei of the sperm are shown with DAPI staining, and flagella are shown with anti-tubulin antibody staining. Scale bar = 10 µm. **(B)** Enlarged view of the head area. The nucleus and mitochondrion of the sperm were stained with DAPI and MitoBright IM Red, respectively. Control experiments were stained without a primary antibody (lower row). Scale bar = 2 µm.

Next, we examined the presence of CatSper proteins. As previously described, the ascidian *CatSper* genes were mainly expressed in the testis but were also weakly expressed in the heart, muscles, and gills. Therefore, we examined whether CatSper proteins were seen in these tissues. In mammals, CatSper proteins were seen only in sperm flagella. In ascidians, Western blotting with an anti-human CatSper3 antibody in each ascidian tissue showed that a band of 50 kDa, the molecular weight of the CatSper3 protein, existed only in the sperm (Figure 3A). Precise observation by immunocytochemistry showed that CatSper3 was localized in the flagella, similar to that in mammalian sperm (Figure 4). Staining with the anti-CatSper3 antibody was also observed in the head region, where mitochondria were located; however, the difference from the control experiment was unclear (Figure 4B), and its presence could not be concluded.

### 3.3 CatSper3-knockout animals delay their development

**FIGURE 5:**
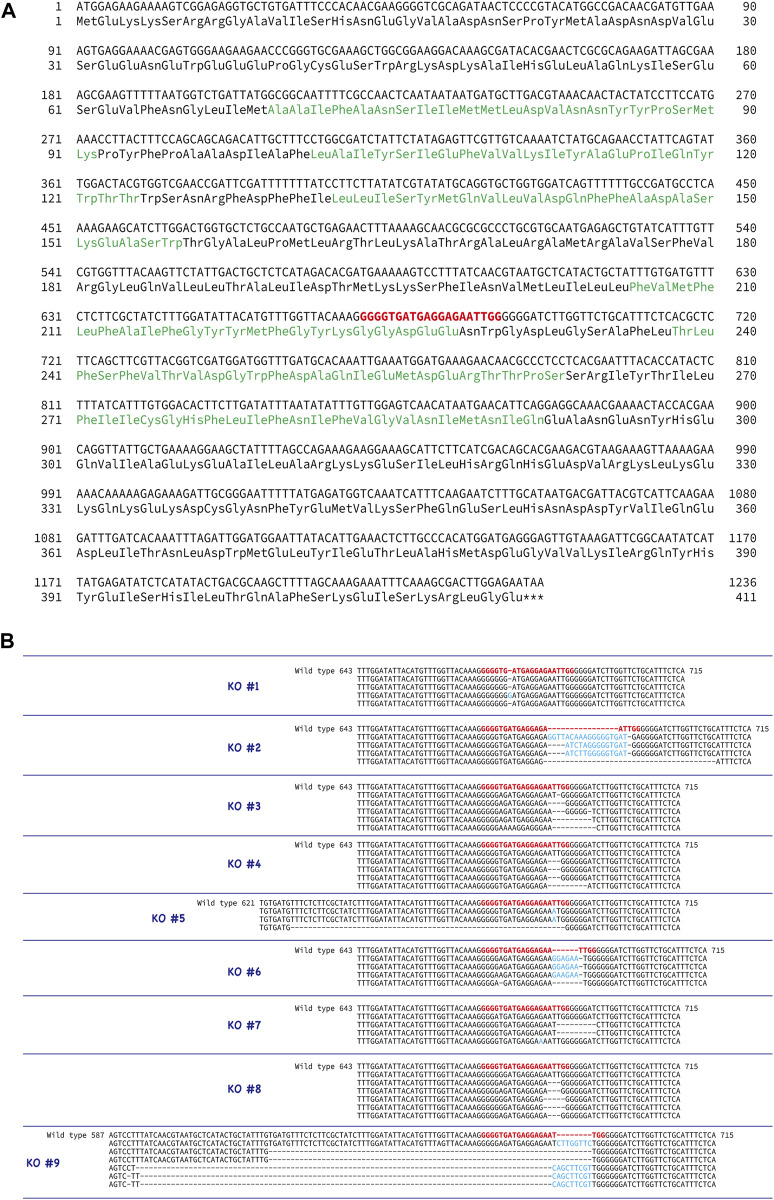
Generation of *CatSper3* KO ascidians. **(A)** DNA and amino acid sequences of *CatSper3*. Target sequence for the CRISPR/Cas9 system is shown in red bold letters. **(B)** Representative DNA sequences around the target sequence of the *CatSper3* KO ascidians with the CRISPR/Cas9 system. The DNA sequence of the un-muted wildtype *CatSper3* is shown in the upper row (wildtype). Bold red letters show the target sequence for the CRISPR/Cas9 system. Deleted and inserted nucleotides are denoted by dashes and blue letters, respectively.

To examine the function of the CatSper channel in ascidian sperm, we tried to produce the *CatSper-*deficient ascidian using the CRISPR/Cas9 system. We selected exon 5 of *CatSper3* as the target gene of the CRISPR/Cas9 system (Figure 5A) because we could not find an effective target site for the other ascidian *CatSper* genes. The mutation frequency on the *CatSper3* gene using the CRISPR/Cas9 system was 94.7% (*n* = 38) when checked at the juvenile stage.

**FIGURE 6:**
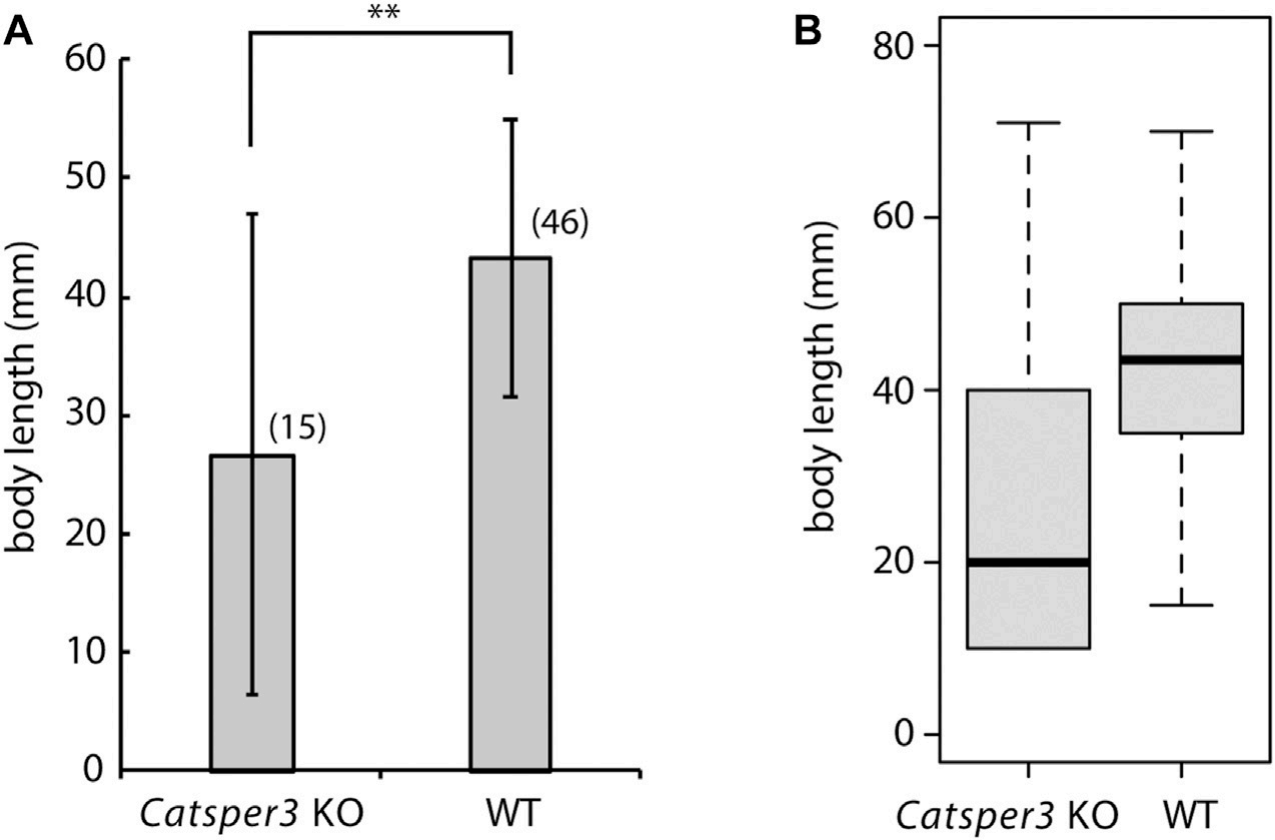
Average **(A)** and box plot **(B)** of body sizes of the *CatSper3* KO and wildtype (WT) animals. The examined KO and WT animals are 60–74 days old and 47–75 days old, respectively. Values are expressed as mean ± S.D. The number in the parenthesis represents the number of examined animals. There is a significant difference between wildtype and KO animals (Student’s *t*-test: **p < 0.01).

Unfortunately, F_0_ animals in which the *CatSper3* gene was edited by the CRISPR/Cas9 system (CatSper3 KO animals) tended to die when their tadpole larvae settled and metamorphosed into juveniles. Furthermore, the growth rate of *CatSper3* KO animals was much slower than that of the control; the body size of the two-month-old individuals was significantly smaller than that of the wildtype animals (Figure 6). Finally, we obtained nine KO animals that reached the mature reproductive stage (Figure 5B). Because gametes from the KO animals could not be fertilized, we used the KO animals for the following analyses.

### 3.4 Spermatogenesis of the CatSper3-knockout animals

In the sperm obtained from CatSper3 KO animals, the 50-kDa CatSper3 protein was reduced or completely lost (Figure 3B). The antibody-reacted band in the Western blotting was seen at 90 kDa and 70 kDa, which was higher than the wildtype CatSper3 protein, and 42 kDa, which was lower than the wildtype CatSper3 protein in some KO sperms and testes (Figure 3B).

**FIGURE 7:**
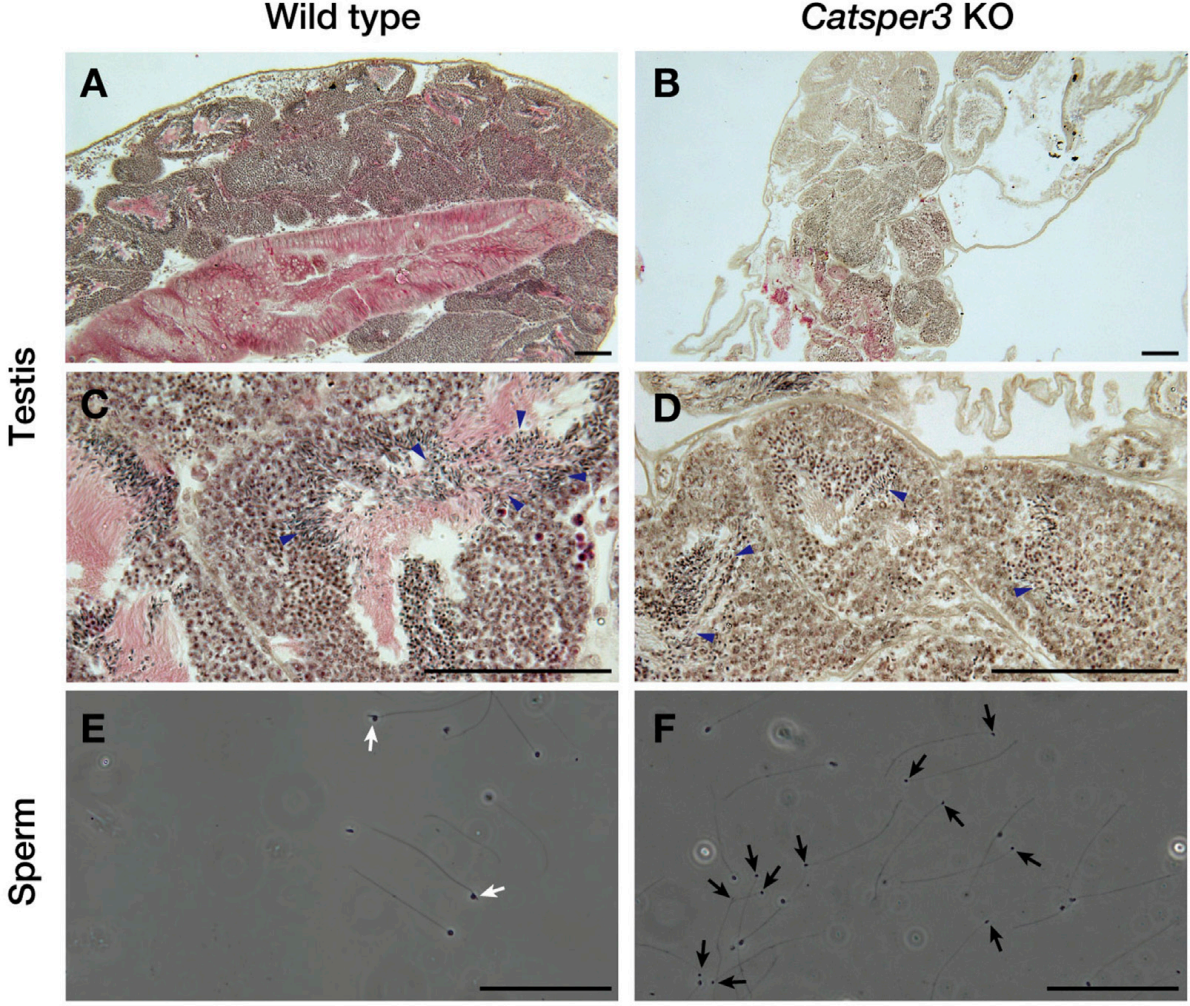
Histological assessment of testes and sperm of the *CatSper3* KO ascidians. **(A–D)** Lower **(A, B)** and higher **(C, D)** magnification of testicular sections stained with hematoxylin and eosin taken from the wildtype **(A, C)** and *CatSper3* KO **(B, D)** ascidians. Arrowheads denote sperm produced in the testis. **(E, F)** Spermatozoa obtained from the spermiduct of the wildtype **(E)** and *CatSper3* KO **(F)** ascidians. Some sperm of the KO animal lost head or have broken flagella (black arrows). The typical head of the wildtype sperm is indicated by white arrows. Scale bar = 50 µm.

The testes of CatSper3 KO animals were smaller than those of wildtype animals at the same age, which was consistent with the poor development of the individuals. Spermatogenesis appeared to occur normally in the KO animals (Figures 7B, D), which yielded mature spermatozoa whose shape appeared normal. However, spermatozoa from KO animals were fragile; headless or broken-flagellar spermatozoa were often observed by microscopy (Figure 7F).

### 3.5 Sperm motility and chemotactic behavior of the CatSper3-knockdown animals

**FIGURE 8:**
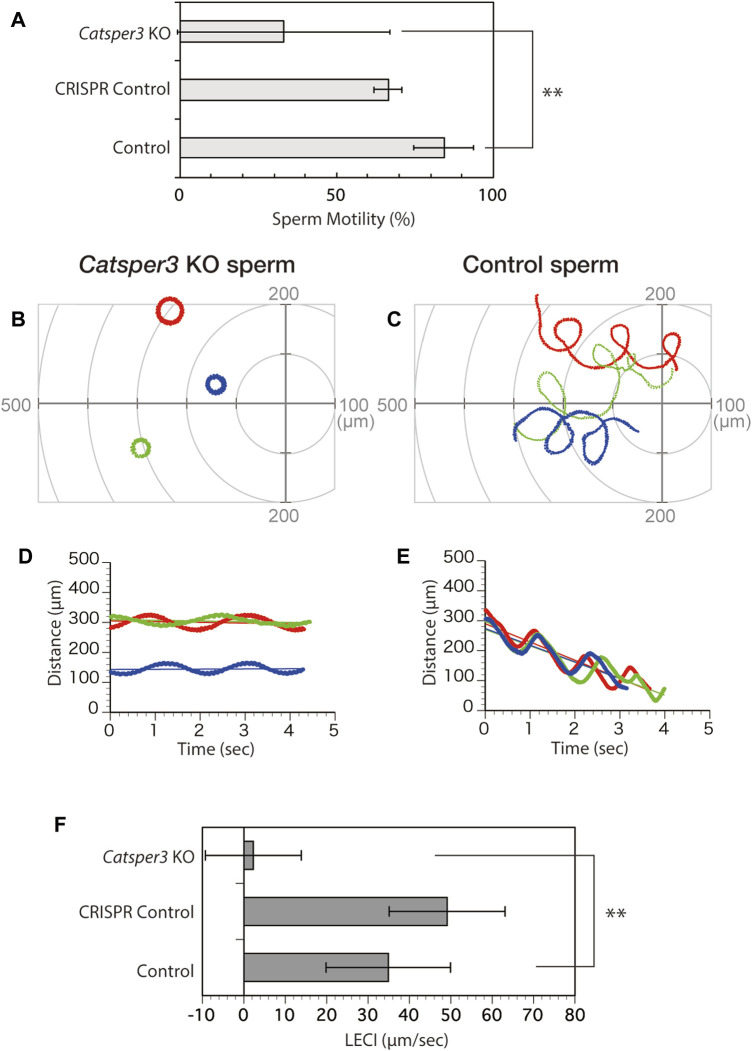
Chemotactic behavior of the CatSper3-knockout sperm. **(A)** Rate of *CatSper3* KO and wildtype spermatozoa with forward motility. *CatSper3* KO, five individuals; control, four individuals. CRISPR control comprises the data on sperm from an animal that was injected with Cas9 and gRNA but did not show any genetic mutation (one individual). Values are expressed as mean ± S.D. Statistical significance was shown with **p < 0.01 (Student’s *t*-test). **(B, C)** Typical three sperm trajectories of *CatSper3* KO **(B)** and wildtype **(C)** animals suspended in seawater around the tip of a capillary containing 5 μM SAAF. The origin of the coordinates indicates the capillary tip. **(D, E)** Changes in the distance between the capillary tip and the head of the swimming sperm of *CatSper3* KO **(D)** and wildtype **(E)** animals shown in **(B)** and **(C)**, respectively. The colors shown in **(B)** and **(C)**, and that shown in **(D)** and **(E)** represent the same sperm. The lines represent the linear equation of time vs. distance. The chemotaxis index (LECI) is calculated as negative values of the coefficients in the equation. **(F)** Chemotaxis indexes of sperm. *CatSper3* KO, *n* = 32 from five individuals; control, *n* = 12 from four individuals. CRISPR control, *n* = 8 from one individual. Values are expressed as mean ± S.D. Statistical significance is shown with **p < 0.01 (Student’s *t*-test).

The sperm appeared normal in shape, but many of the sperms had no motility, even in the presence of theophylline, which is an activating agent for ascidian sperm motility (Figure 8, Supplementary Movies S1, S2). A few spermatozoa showed forward motility, and those seemed to have a large lateral amplitude of the head (Supplementary Movie S2, Figure 8), similar to sperm movement in the presence of a PMCA inhibitor (Yoshida et al., 2018). None of the spermatozoa exhibited chemotactic responses (Figure 8).

## 4 Discussion

In the 20 years since the discovery of CatSper as a sperm-specific Ca^2+^ channel (Ren et al., 2001), several studies have shown that CatSper is the only important functional Ca^2+^ channel in mammalian sperm (Lishko and Mannowetz, 2018). CatSper is localized at linear quadrilateral nanodomains along the flagellum in the mammalian sperm, and it mediates rheotactic behavior in the female reproductive tract (Chung et al., 2014; Chung et al., 2017). However, many animals, including birds and amphibians, lack the CatSper genes (Cai and Clapham, 2008), and it is not known whether CatSper has the same functions in the sperm of animals other than mammals, although only in the sea urchin does CatSper seem to mediate chemotactic behavior according to pharmacological studies (Rennhack et al., 2018). The ascidian *C. intestinalis* is a chordate and has four CatSper isoforms (CatSper 1–4), as in mammals. Interestingly, CatSper does not seem to be restricted to sperm, and our study shows that ascidian CatSper expression was not restricted to the testis but was also observed in the heart, gills, ovary, and muscles, even though normal CatSper protein was only observed in sperm. CatSper in ascidian sperm appeared to be localized in the flagellum, similar to that in mammalian sperm (Figure 4). Furthermore, most *CatSper3*-KO ascidians died within a month because of stunted growth, and the body size of mature *CatSper3*-KO ascidians was significantly small. These data suggest that CatSper plays a role in development and/or nutrient uptake. Although the expression area of CatSper3 in organs other than the testes has not been examined, the cilia seemed to be immunoreactive to the anti-CatSper3 antibody. Thus, CatSper might be expressed in the cilia and flagella, not only in the sperm but also in the whole body, and play some role in the movement of the cilia/flagella.

The shape of the sperm obtained from the *CatSper3*-KO ascidians was almost normal, but they were fragile; the head was easily disconnected from the flagellum (Figure 7). Furthermore, the spermatozoa were almost quiescent and never activated by any stimulation. Some sperm were motile but did not show chemotactic behavior. In the mouse, there are no morphological differences between the wildtype and the *CatSper*-KO sperm in any subunits (Ren et al., 2001; Quill et al., 2003; Liu et al., 2007; Qi et al., 2007; Chung et al., 2011; Chung et al., 2017; Hwang et al., 2019). In contrast, spermatogenesis in the *CatSper3*-KO male individual did not appear to differ from that in the wildtype, although the testes in KO animals were smaller than those in the wildtype (Figure 7). Whether morphological abnormalities in spermatozoa are due to abnormal spermatogenesis or low nutrition caused by eating disorders requires further detailed analysis.

*CatSper3*-KO sperm did not show any response to SAAF, and no chemotactic behavior was observed. This result is consistent with the pharmacological findings on sperm chemotaxis in the sea urchin, *Arbacia punctulata* (Seifert et al., 2015). In mammals, the sperm lacking CatSper are motile but cannot show hyperactivation, resulting in infertility (Ren et al., 2001; Qi et al., 2007). Therefore, CatSper may be involved in the regulation of flagellar motility in ascidians, sea urchins, and mammals. In mammals, CatSper is assumed to be a direct or indirect target of various chemicals and is involved in chemotaxis (Brenker et al., 2012). In humans, progesterone, which is a putative sperm attractant, has been shown to activate CatSper through binding to α/β hydrolase domain-containing protein 2 (Miller et al., 2016). Furthermore, subunits of CatSper, EFCAB9 and Catsperζ, regulate the opening of CatSper channels in response to changes in intracellular Ca^2+^ and pH (Hwang et al., 2019). On the other hand, in ascidians, the sperm attractant SAAF directly binds to PMCA and seems to induce the Ca^2+^ efflux from the sperm (Yoshida et al., 2018). Furthermore, the *CatSper3*-KO sperm not only lacked chemotactic behavior but also had significant effects on motility itself, with many sperms failing to initiate movement. In mammals, sperms lacking any pore-forming CatSper subunits have similar initial velocities, as their wildtype counterparts (Qi et al., 2007), and CatSperζ-null and EFCAB9-null spermatozoa are also motile, although they show abnormal flagellar waveforms (Chung et al., 2017; Hwang et al., 2019). On the other hand, as for the ascidian auxiliary subunits of CatSper, *ß* (KH.C9.522), γ (KH.C11.410), EFCAB9 (KY21. Chr7.569), and ABHD2 (KY21. Chr3.1310) have been found in the *Ciona* genome database; however, *δ*, *ε*, *ζ*, and *η* have not been found and do not appear to exist. Thus, the system regulating sperm flagellar beating, including the CatSper channel, seems to differ from that in mammals, even though it is mainly regulated by [Ca^2+^]_i_: CatSper is also an indispensable Ca^2+^ channel in ascidian sperm, but it plays roles not only in sperm flagellar beatings but also in fundamental sperm motility.

In addition, although an increase in pH is necessary for the activation of ascidian sperm, sperm chemotaxis itself is observed even under low pH conditions (Sensui et al., 2023). This suggests that ascidian CatSper is involved in motility activation rather than chemotaxis.

In conclusion, this study indicates that CatSper plays an important role in ascidian sperm development. However, unlike in mammals, CatSper seems to be involved not only in the regulation of sperm flagellar beatings but also in sperm motility. Therefore, CatSper may play a role in development and spermatogenesis. Notably, although the role of CatSper in ascidians appears to be more important than that in mammals, this may only be due to impaired spermatogenesis, and more detailed studies are needed. We would like to identify the differences in CatSper roles.

## Data Availability

The datasets presented in this study can be found in online repositories. The names of the repository/repositories and accession number(s) can be found in the article/Supplementary Material.
