# Supplementary material for: CatSper mediates not only chemotactic behavior but also the motility of ascidian sperm
Source: Front Cell Dev Biol. 2023 Nov 2;11:1136537. doi: 10.3389/fcell.2023.1136537 (PMC10652287; doi:10.3389/fcell.2023.1136537)
Supplement: Supplementary file 1 [file DataSheet1.zip › Fig_S2.pdf]

Assay Class: Eukaryote Total RNA Nano
Data Path: C:\...Eukaryote Total RNA Nano_DE54108122_2023-05-03_11-55-39.xad

Created: 5/3/2023 11:55:38 AM
Modified: 5/3/2023 12:19:27 PM

Electrophoresis File Run Summary

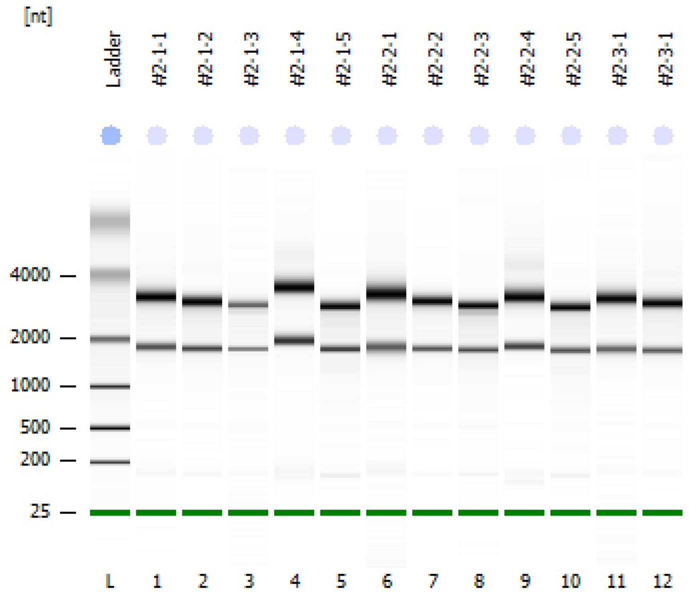

Instrument Information:

Instrument Name: DE54108122 Firmware: C.01.069

Serial#: DE54108122 Type: G2939A

Assay Information:

Assay Origin Path: C:\Program Files (x86)\Agilent\2100 bioanalyzer\2100 expert\assays\RNA\Eukaryote Total RNA Nano Series II.xsy

Assay Class: Eukaryote Total RNA Nano

Version: 2.6

Assay Comments: Total RNA Analysis ng sensitivity (Eukaryote)

© Copyright 2003 - 2009 Agilent Technologies, Inc.

Chip Information:

Chip Lot #:

Reagent Kit Lot #:

Chip Comments:

#2-1-1

RIN:10

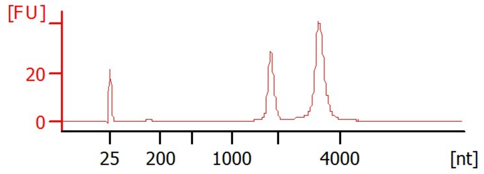

#2-1-2

RIN:10

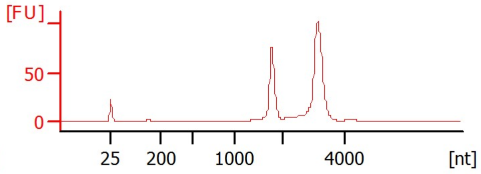

#2-1-3

RIN:10

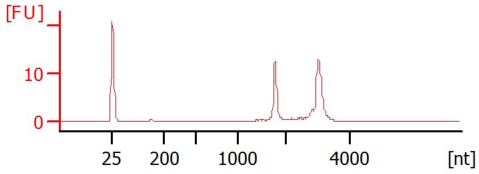

#2-1-4

RIN:10

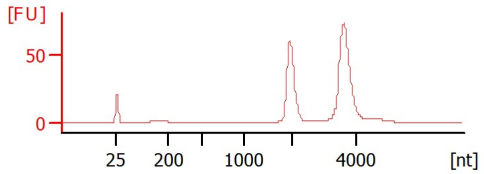

#2-1-5

RIN: 9.80

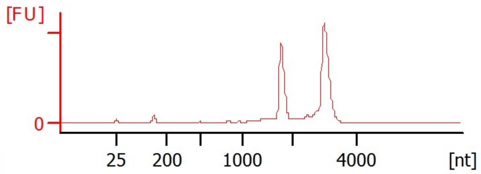

#2-2-1

RIN:10

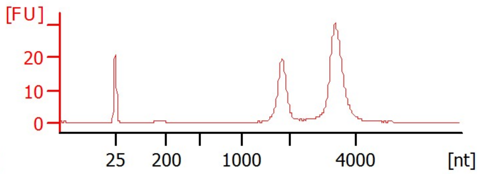

#2-2-2

RIN:10

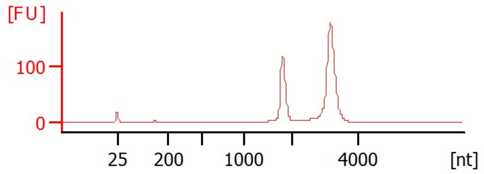

#2-2-3

RIN:10

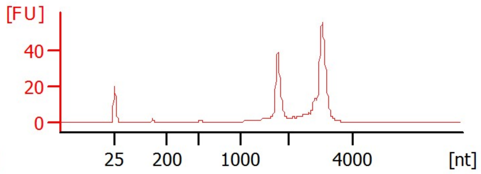

#2-2-4

RIN:10

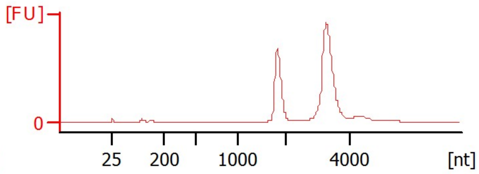

#2-2-5

RIN: 9.80

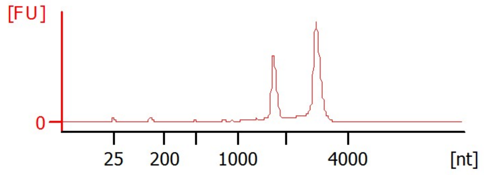

#2-3-1

RIN:10

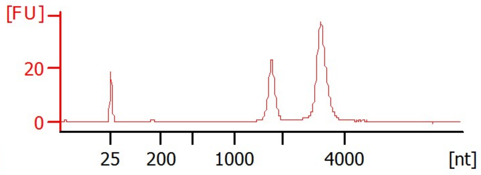

#2-3-1

RIN:10

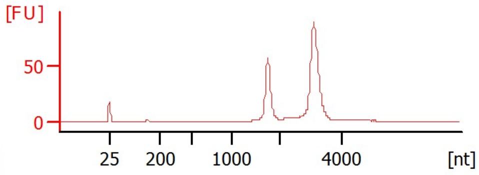

 © Copyright 2003 - 2015 Agilent Technologies, Inc.

Assay Class: Eukaryote Total RNA Nano
Data Path: C:\...Eukaryote Total RNA Nano_DE54108122_2023-05-03_11-55-39.xad
Created: 5/3/2023 11:55:38 AM
Modified: 5/3/2023 12:19:27 PM

Electrophoresis File Run Summary (Chip Summary)

Sample Name	Sample Comment	Status	Result Label	Result Color
#2-1-1		✔	RIN:10	
#2-1-2		✔	RIN:10	
#2-1-3		✔	RIN:10	
#2-1-4		✔	RIN:10	
#2-1-5		✔	RIN: 9.80	
#2-2-1		✔	RIN:10	
#2-2-2		✔	RIN:10	
#2-2-3		✔	RIN:10	
#2-2-4		✔	RIN:10	
#2-2-5		✔	RIN: 9.80	
#2-3-1		✔	RIN:10	
#2-3-1		✔	RIN:10	
Ladder		✔	All Other Samples	

Chip Lot #

Reagent Kit Lot #

Chip Comments :

© Copyright 2003 - 2015 Agilent Technologies, Inc.

Assay Class: Eukaryote Total RNA Nano
Data Path: C:\...Eukaryote Total RNA Nano_DE54108122_2023-05-03_11-55-39.xad
Created: 5/3/2023 11:55:38 AM
Modified: 5/3/2023 12:19:27 PM

Electrophoresis Assay Details

General Analysis Settings

Number of Available Sample and Ladder Wells (Max.) : 13
Minimum Visible Range [s] : 17
Maximum Visible Range [s] : 70
Start Analysis Time Range [s] : 19
End Analysis Time Range [s] : 69
Ladder Concentration [ng/µl] : 150
Lower Marker Concentration [ng/µl] : 0
Upper Marker Concentration [ng/µl] : 0
Used Lower Marker for Quantitation
Standard Curve Fit is Logarithmic
Show Data Aligned to Lower Marker

Integrator Settings

Integration Start Time [s] : 19
Integration End Time [s] : 69
Slope Threshold : 0.6
Height Threshold [FU] : 0.5
Area Threshold : 0.2
Width Threshold [s] : 0.5
Baseline Plateau [s] : 6

Filter Settings

Filter Width [s] : 0.5
Polynomial Order : 4

Ladder

Ladder Peak	Size
1	25
2	200
3	500
4	1000
5	2000
6	4000

 © Copyright 2003 - 2015 Agilent Technologies, Inc.

Assay Class: Eukaryote Total RNA Nano
Data Path: C:\...Eukaryote Total RNA Nano_DE54108122_2023-05-03_11-55-39.xad

Created: 5/3/2023 11:55:38 AM
Modified: 5/3/2023 12:19:27 PM

Electropherogram Summary

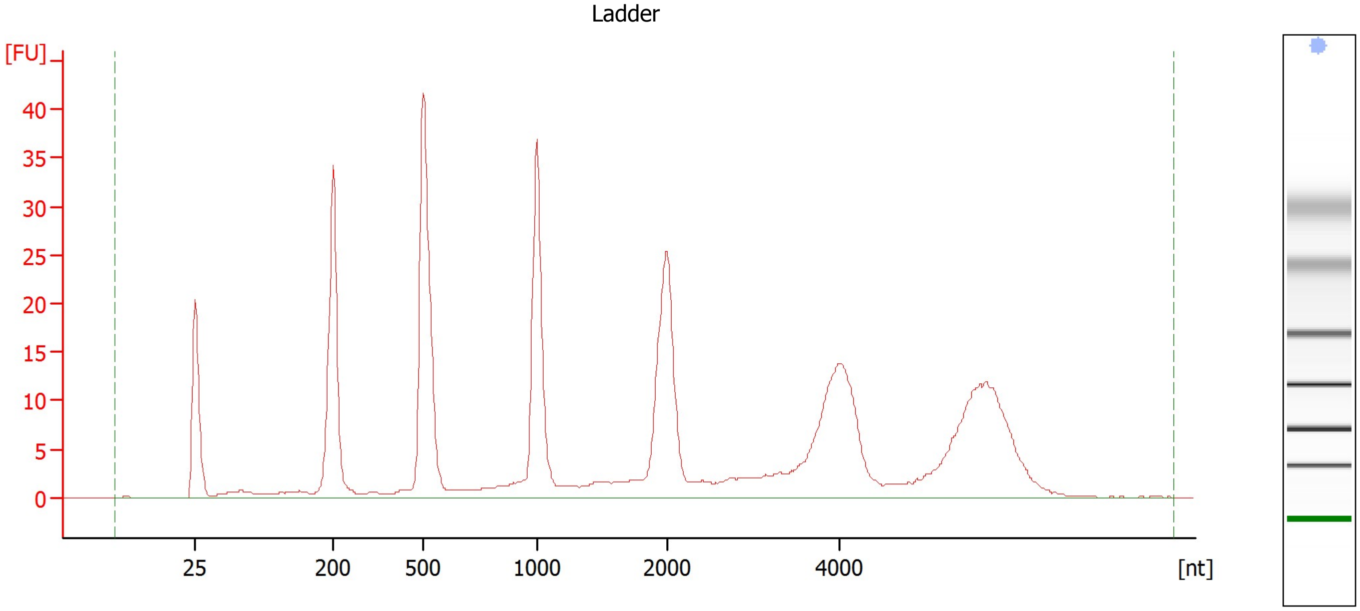

Overall Results for Ladder

RNA Area:	395.5	Result Flagging Color:	
RNA Concentration:	150 ng/µl	Result Flagging Label:	All Other Samples

 © Copyright 2003 - 2015 Agilent Technologies, Inc.

Assay Class: Eukaryote Total RNA Nano
Data Path: C:\...Eukaryote Total RNA Nano_DE54108122_2023-05-03_11-55-39.xad
Created: 5/3/2023 11:55:38 AM
Modified: 5/3/2023 12:19:27 PM

Electropherogram Summary Continued ...

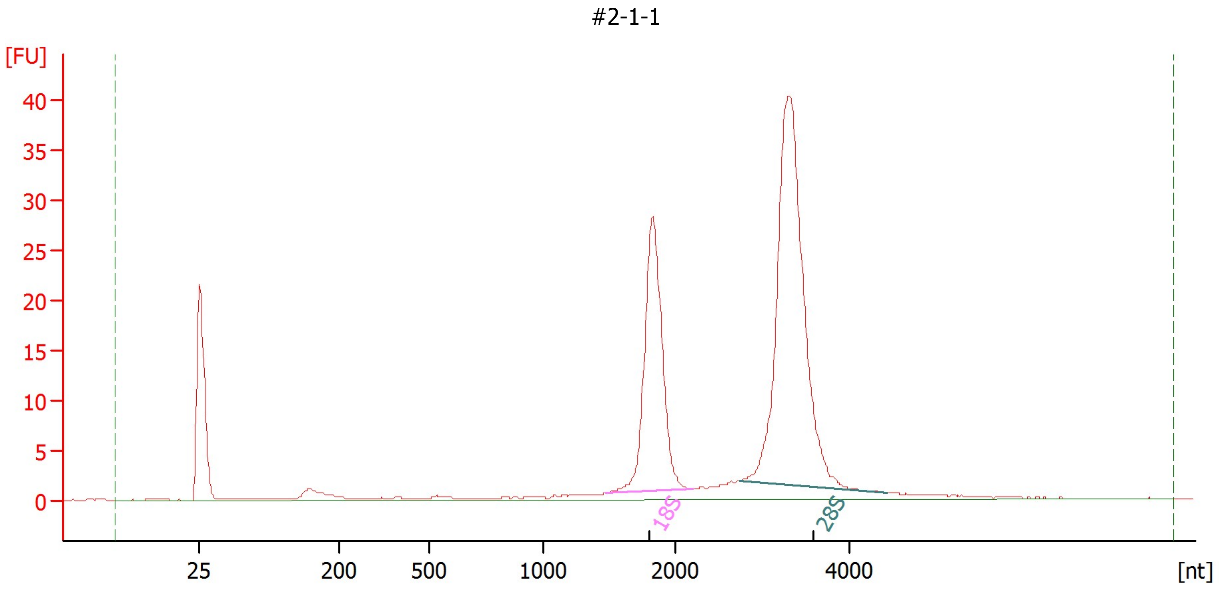

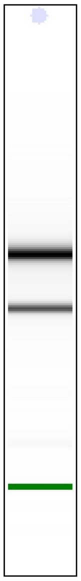

Overall Results for sample 1 : #2-1-1

RNA Area:	217.6	RNA Integrity Number (RIN):	10 (B.02.08)
RNA Concentration:	83 ng/µl	Result Flagging Color:	
rRNA Ratio [28s / 18s]:	1.9	Result Flagging Label:	RIN:10

Fragment table for sample 1 : #2-1-1

Name	Start Size [nt]	End Size [nt]	Area	% of total Area
18S	1,473	2,198	59.0	27.1
28S	2,736	4,426	109.3	50.3

© Copyright 2003 - 2015 Agilent Technologies, Inc.

Assay Class: Eukaryote Total RNA Nano
Data Path: C:\...Eukaryote Total RNA Nano_DE54108122_2023-05-03_11-55-39.xad
Created: 5/3/2023 11:55:38 AM
Modified: 5/3/2023 12:19:27 PM

Electropherogram Summary Continued ...

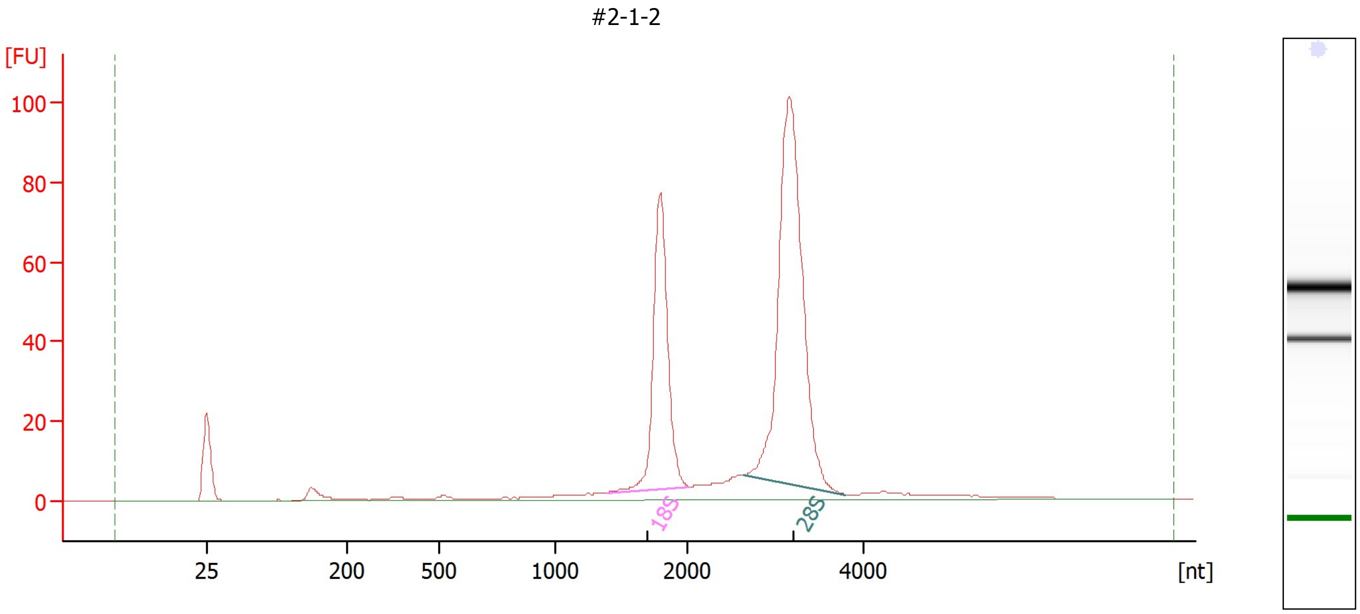

Overall Results for sample 2 : #2-1-2

RNA Area:	511.0	RNA Integrity Number (RIN):	10 (B.02.08)
RNA Concentration:	194 ng/µl	Result Flagging Color:	
rRNA Ratio [28s / 18s]:	1.9	Result Flagging Label:	RIN:10

Fragment table for sample 2 : #2-1-2

Name	Start Size [nt]	End Size [nt]	Area	% of total Area
18S	1,394	2,030	127.2	24.9
28S	2,634	3,782	246.4	48.2

© Copyright 2003 - 2015 Agilent Technologies, Inc.

Assay Class: Eukaryote Total RNA Nano
Data Path: C:\...Eukaryote Total RNA Nano_DE54108122_2023-05-03_11-55-39.xad
Created: 5/3/2023 11:55:38 AM
Modified: 5/3/2023 12:19:27 PM

Electropherogram Summary Continued ...

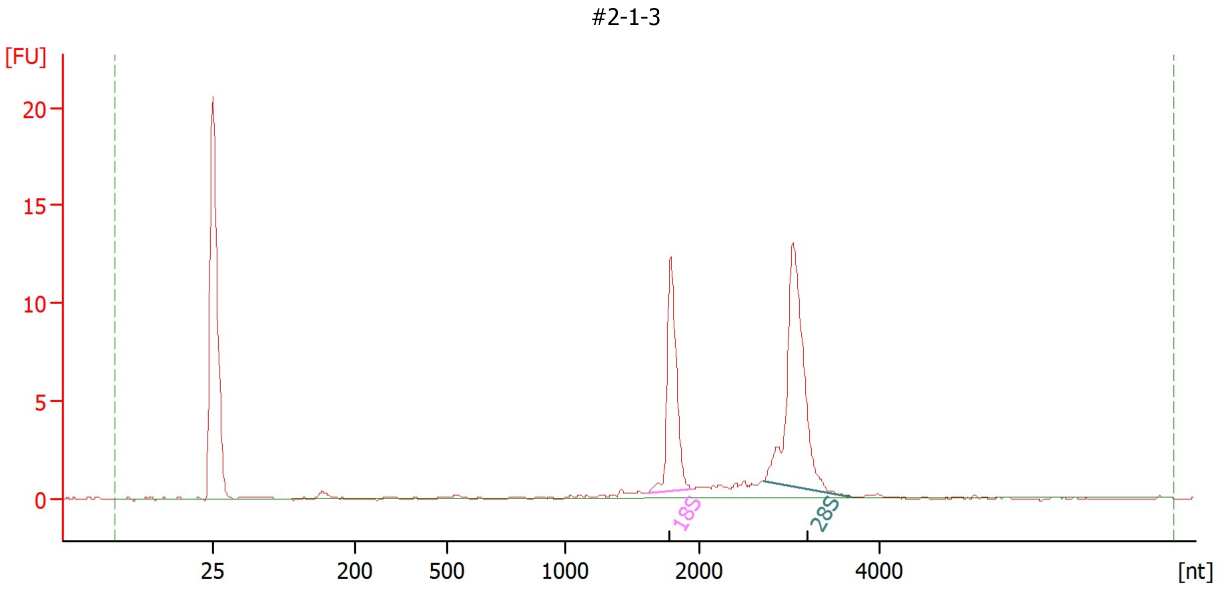

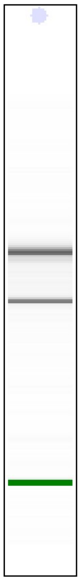

Overall Results for sample 3 : #2-1-3

RNA Area:	51.2	RNA Integrity Number (RIN):	10 (B.02.08)
RNA Concentration:	19 ng/µl	Result Flagging Color:	
rRNA Ratio [28s / 18s]:	1.7	Result Flagging Label:	RIN:10

Fragment table for sample 3 : #2-1-3

Name	Start Size [nt]	End Size [nt]	Area	% of total Area
18S	1,610	1,935	13.4	26.1
28S	2,714	3,692	22.6	44.1

 © Copyright 2003 - 2015 Agilent Technologies, Inc.

Assay Class: Eukaryote Total RNA Nano
Data Path: C:\...Eukaryote Total RNA Nano_DE54108122_2023-05-03_11-55-39.xad
Created: 5/3/2023 11:55:38 AM
Modified: 5/3/2023 12:19:27 PM

Electropherogram Summary Continued ...

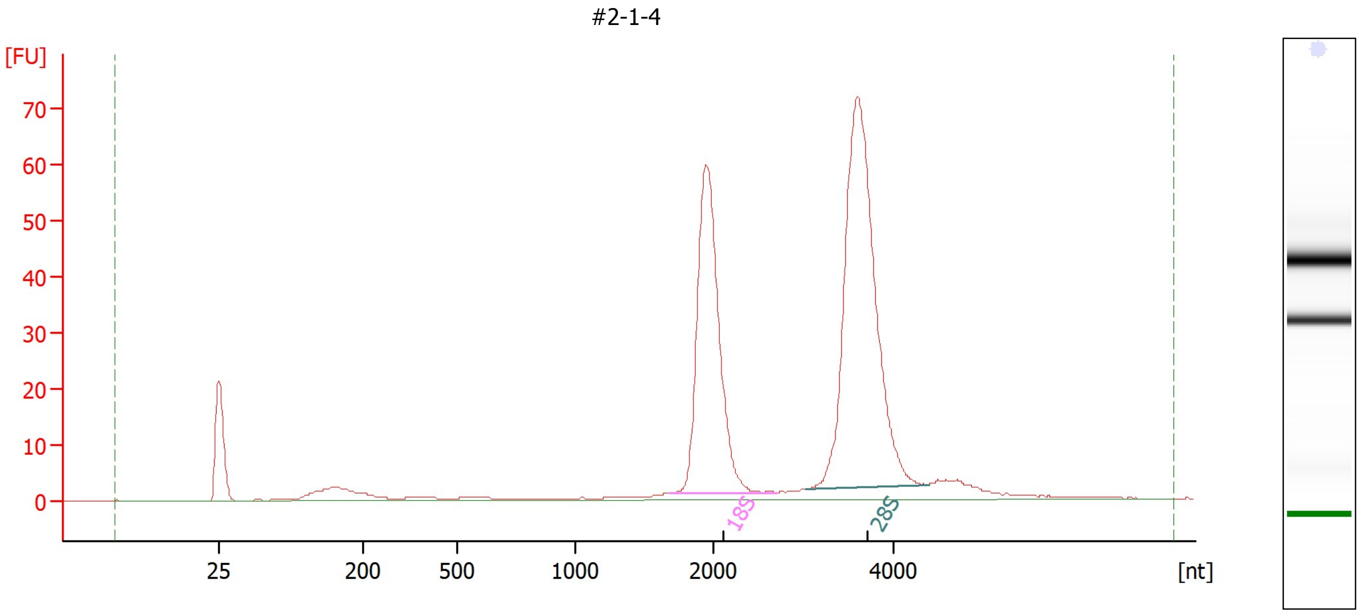

Overall Results for sample 4 : #2-1-4

RNA Area:	441.5	RNA Integrity Number (RIN):	10 (B.02.08)
RNA Concentration:	167 ng/µl	Result Flagging Color:	
rRNA Ratio [28s / 18s]:	1.4	Result Flagging Label:	RIN:10

Fragment table for sample 4 : #2-1-4

Name	Start Size [nt]	End Size [nt]	Area	% of total Area
18S	1,688	2,708	145.7	33.0
28S	3,015	4,382	208.9	47.3

 © Copyright 2003 - 2015 Agilent Technologies, Inc.

Assay Class: Eukaryote Total RNA Nano
Data Path: C:\...Eukaryote Total RNA Nano_DE54108122_2023-05-03_11-55-39.xad
Created: 5/3/2023 11:55:38 AM
Modified: 5/3/2023 12:19:27 PM

Electropherogram Summary Continued ...

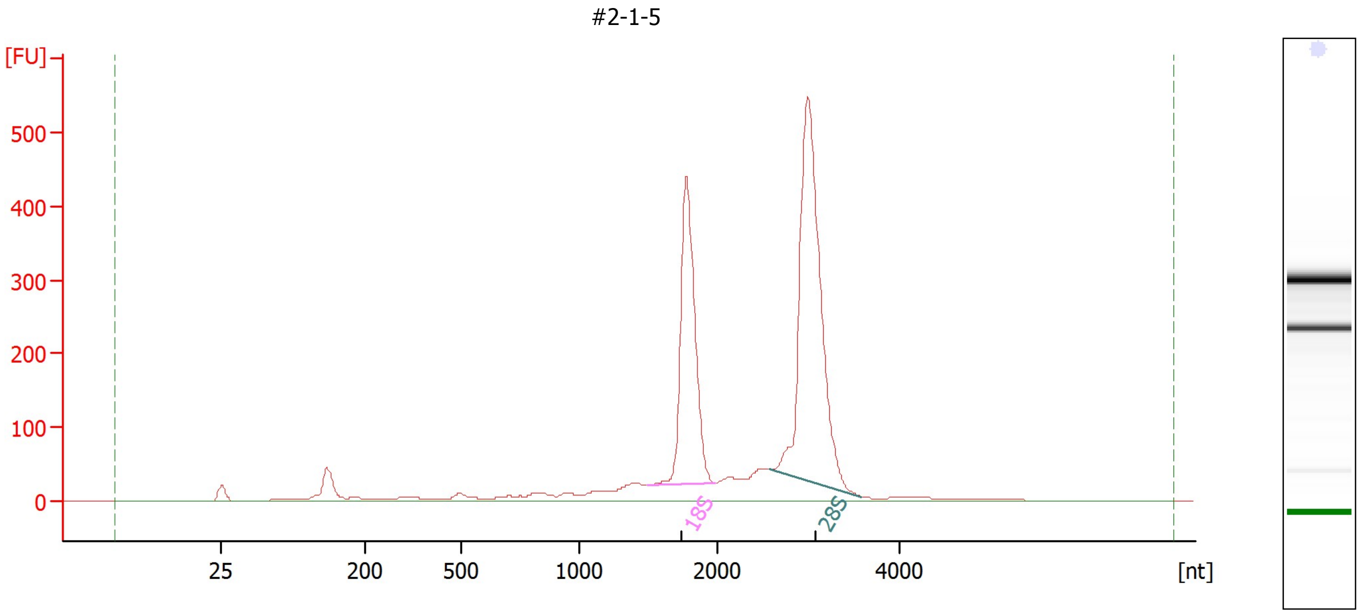

Overall Results for sample 5 : #2-1-5

RNA Area:	2,808.4	RNA Integrity Number (RIN):	9.8 (B.02.08)
RNA Concentration:	1,065 ng/µl	Result Flagging Color:	
rRNA Ratio [28s / 18s]:	1.6	Result Flagging Label:	RIN: 9.80

Fragment table for sample 5 : #2-1-5

Name	Start Size [nt]	End Size [nt]	Area	% of total Area
18S	1,495	1,986	687.6	24.5
28S	2,578	3,587	1,111.8	39.6

 © Copyright 2003 - 2015 Agilent Technologies, Inc.

Assay Class: Eukaryote Total RNA Nano
Data Path: C:\...Eukaryote Total RNA Nano_DE54108122_2023-05-03_11-55-39.xad
Created: 5/3/2023 11:55:38 AM
Modified: 5/3/2023 12:19:27 PM

Electropherogram Summary Continued ...

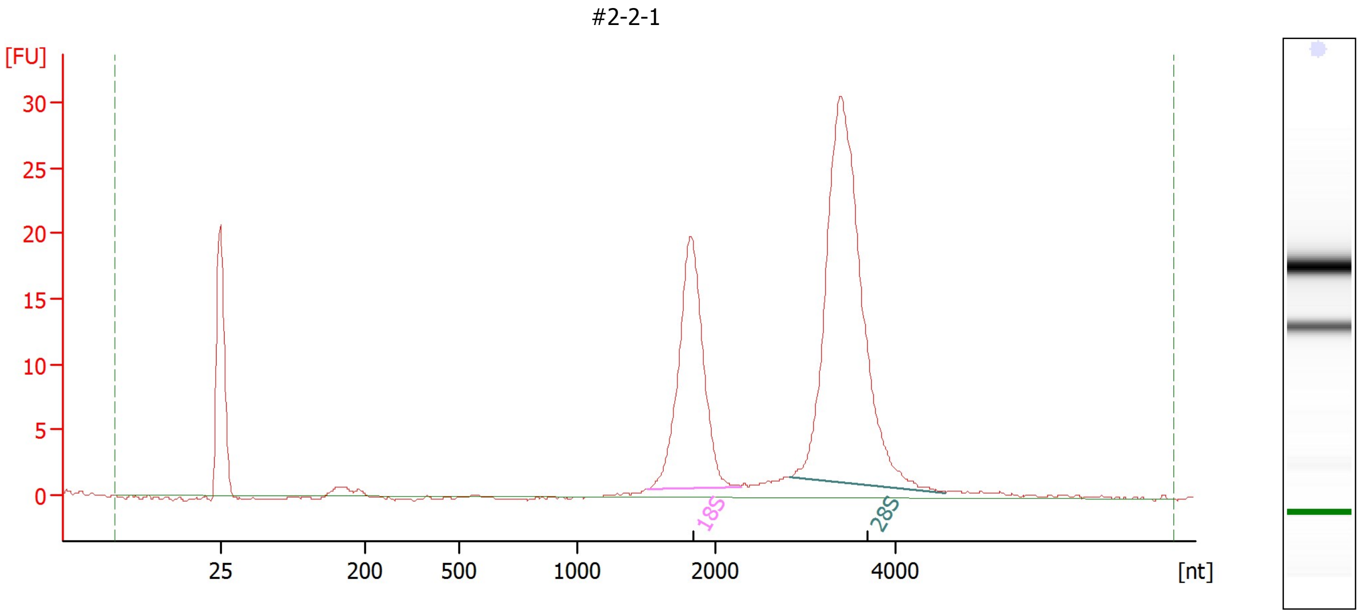

Overall Results for sample 6 : #2-2-1

RNA Area:	190.5	RNA Integrity Number (RIN):	10 (B.02.08)
RNA Concentration:	72 ng/µl	Result Flagging Color:	
rRNA Ratio [28s / 18s]:	1.9	Result Flagging Label:	RIN:10

Fragment table for sample 6 : #2-2-1

Name	Start Size [nt]	End Size [nt]	Area	% of total Area
18S	1,501	2,284	54.9	28.8
28S	2,813	4,543	105.4	55.3

© Copyright 2003 - 2015 Agilent Technologies, Inc.

Assay Class: Eukaryote Total RNA Nano
Data Path: C:\...Eukaryote Total RNA Nano_DE54108122_2023-05-03_11-55-39.xad
Created: 5/3/2023 11:55:38 AM
Modified: 5/3/2023 12:19:27 PM

Electropherogram Summary Continued ...

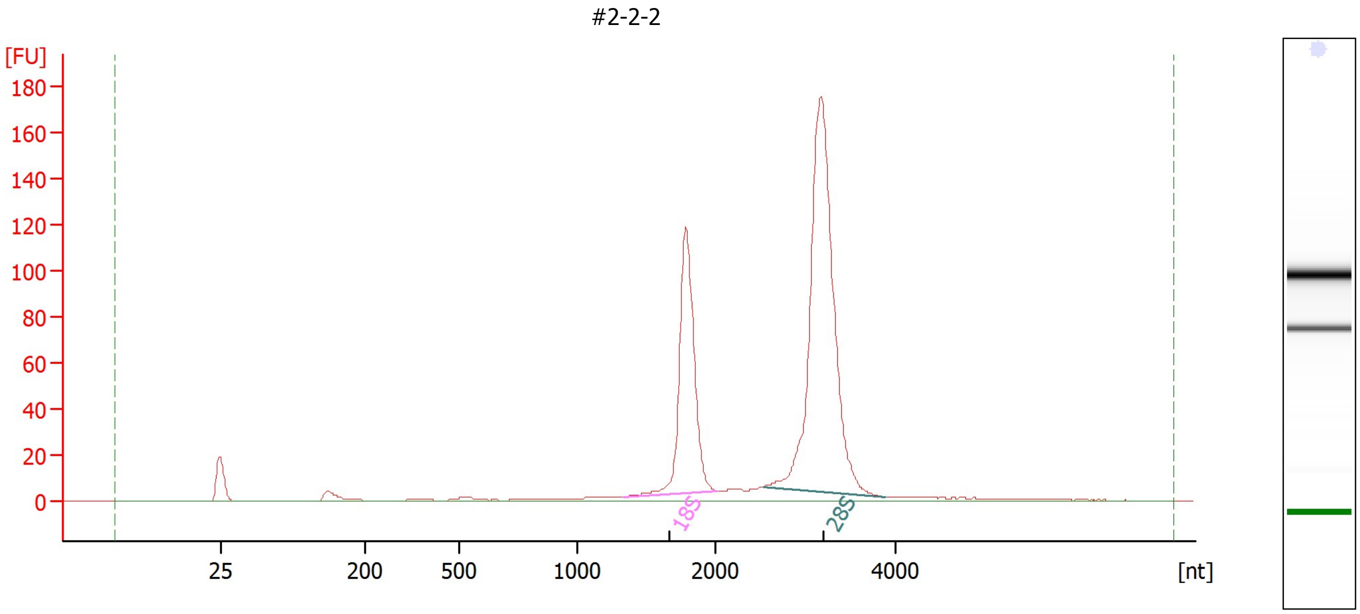

Overall Results for sample 7 : #2-2-2

RNA Area:	757.2	RNA Integrity Number (RIN):	10 (B.02.08)
RNA Concentration:	287 ng/µl	Result Flagging Color:	
rRNA Ratio [28s / 18s]:	2.0	Result Flagging Label:	RIN:10

Fragment table for sample 7 : #2-2-2

Name	Start Size [nt]	End Size [nt]	Area	% of total Area
18S	1,337	2,037	199.2	26.3
28S	2,543	3,872	407.6	53.8

© Copyright 2003 - 2015 Agilent Technologies, Inc.

Assay Class: Eukaryote Total RNA Nano
Data Path: C:\...Eukaryote Total RNA Nano_DE54108122_2023-05-03_11-55-39.xad

Created: 5/3/2023 11:55:38 AM
Modified: 5/3/2023 12:19:27 PM

Electropherogram Summary Continued ...

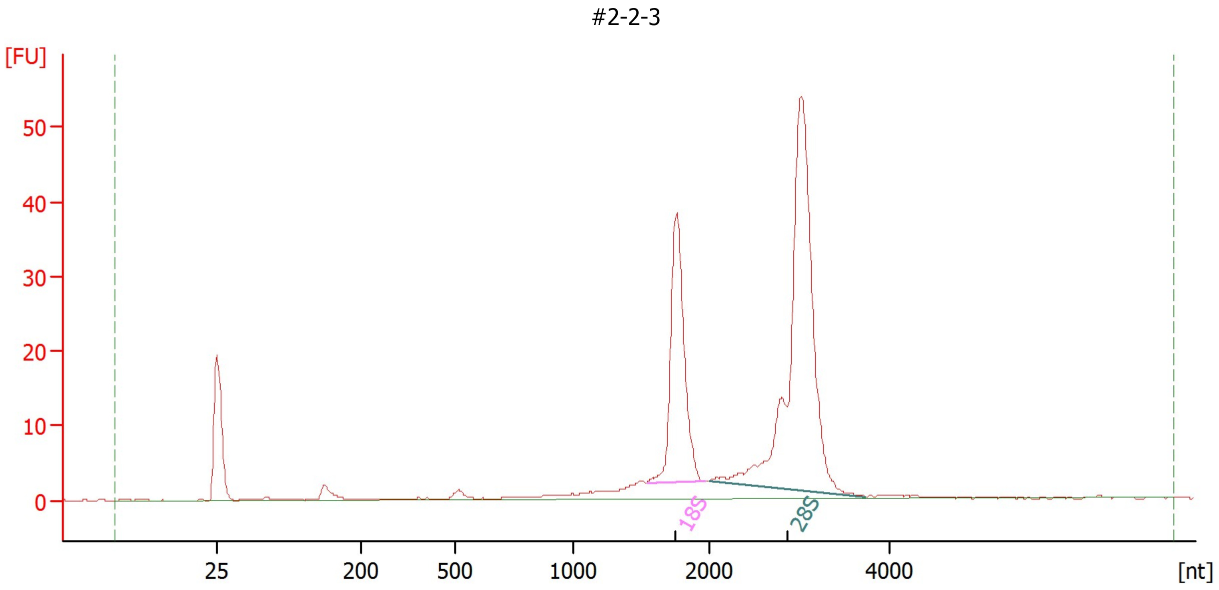

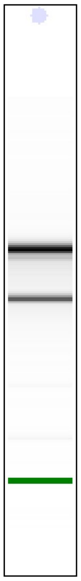

Overall Results for sample 8 : #2-2-3

RNA Area:	246.0	RNA Integrity Number (RIN):	10 (B.02.08)
RNA Concentration:	93 ng/µl	Result Flagging Color:	
rRNA Ratio [28s / 18s]:	2.2	Result Flagging Label:	RIN:10

Fragment table for sample 8 : #2-2-3

Name	Start Size [nt]	End Size [nt]	Area	% of total Area
18S	1,544	1,984	57.0	23.2
28S	2,000	3,728	125.7	51.1

 © Copyright 2003 - 2015 Agilent Technologies, Inc.

Assay Class:	Eukaryote Total RNA Nano	Created:	5/3/2023 11:55:38 AM
Data Path:	C:\...Eukaryote Total RNA Nano_DE54108122_2023-05-03_11-55-39.xad	Modified:	5/3/2023 12:19:27 PM

Electropherogram Summary Continued ...

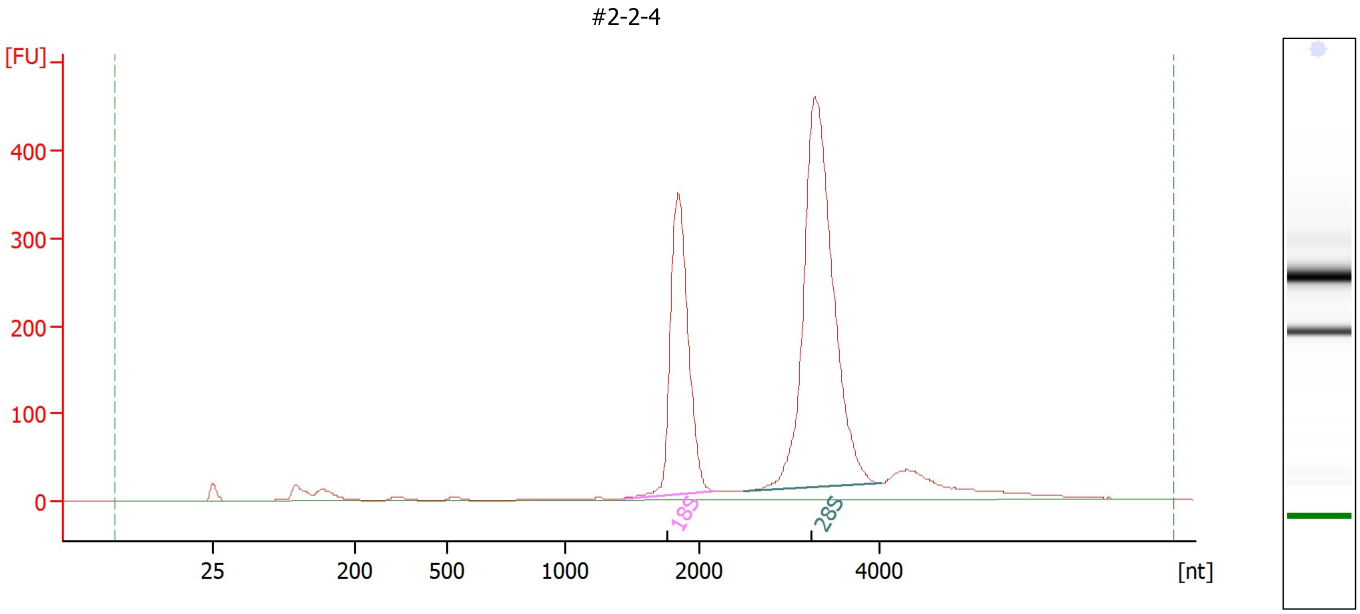

Overall Results for sample 9 : #2-2-4

RNA Area:	2,731.5	RNA Integrity Number (RIN):	10 (B.02.08)
RNA Concentration:	1,036 ng/µl	Result Flagging Color:	
rRNA Ratio [28s / 18s]:	1.9	Result Flagging Label:	RIN:10

Fragment table for sample 9 : #2-2-4

Name	Start Size [nt]	End Size [nt]	Area	% of total Area
18S	1,420	2,165	719.2	26.3
28S	2,475	4,014	1,363.0	49.9

 © Copyright 2003 - 2015 Agilent Technologies, Inc.

Assay Class: Eukaryote Total RNA Nano
Data Path: C:\...Eukaryote Total RNA Nano_DE54108122_2023-05-03_11-55-39.xad
Created: 5/3/2023 11:55:38 AM
Modified: 5/3/2023 12:19:27 PM

Electropherogram Summary Continued ...

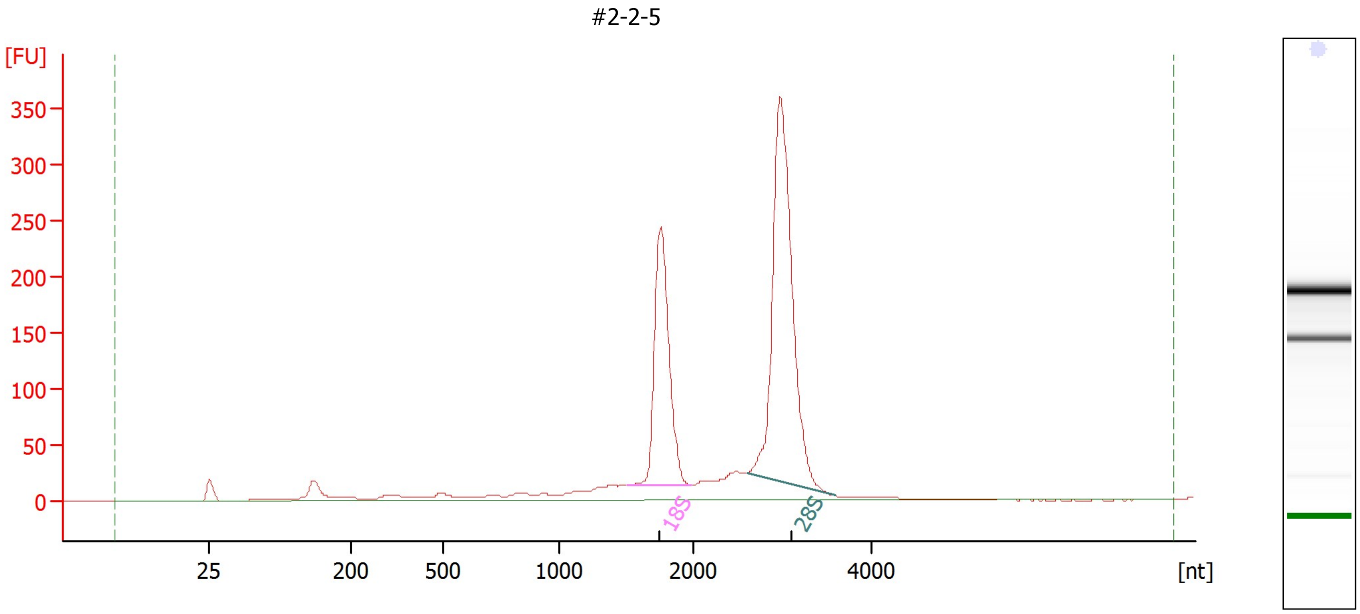

Overall Results for sample 10 : #2-2-5

RNA Area:	1,698.8	RNA Integrity Number (RIN):	9.8 (B.02.08)
RNA Concentration:	644 ng/µl	Result Flagging Color:	
rRNA Ratio [28s / 18s]:	1.8	Result Flagging Label:	RIN: 9.80

Fragment table for sample 10 : #2-2-5

Name	Start Size [nt]	End Size [nt]	Area	% of total Area
18S	1,503	1,981	401.6	23.6
28S	2,596	3,593	719.4	42.3

 © Copyright 2003 - 2015 Agilent Technologies, Inc.

Assay Class:	Eukaryote Total RNA Nano	Created:	5/3/2023 11:55:38 AM
Data Path:	C:\...Eukaryote Total RNA Nano_DE54108122_2023-05-03_11-55-39.xad	Modified:	5/3/2023 12:19:27 PM

Electropherogram Summary Continued ...

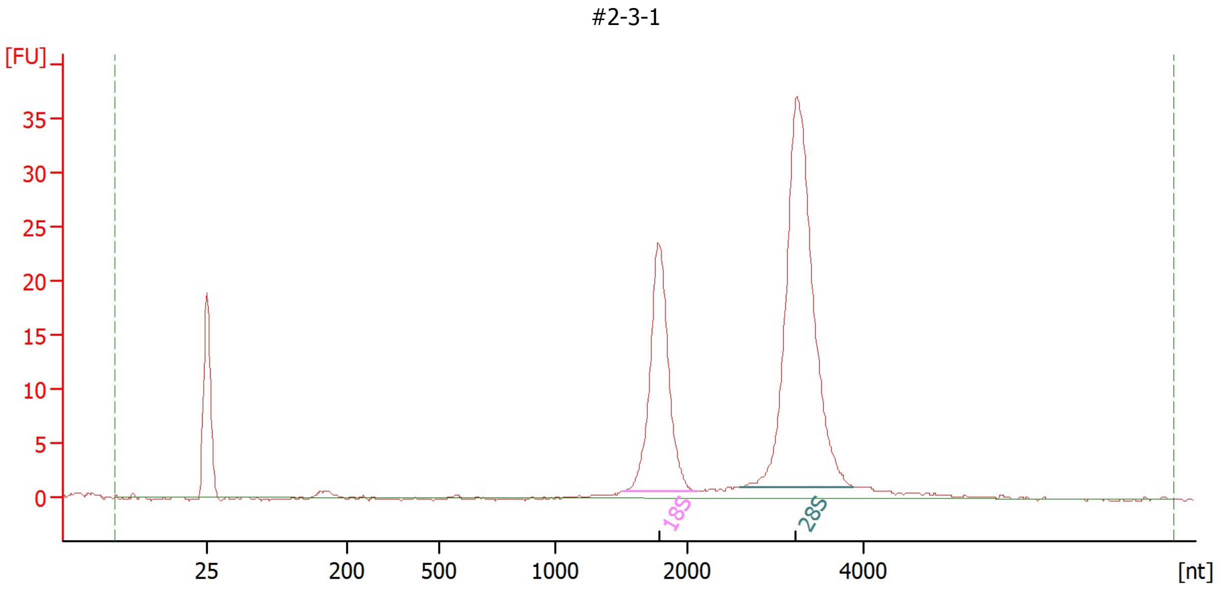

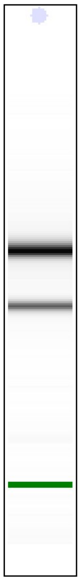

Overall Results for sample 11 : #2-3-1

RNA Area:	182.8	RNA Integrity Number (RIN):	10 (B.02.08)
RNA Concentration:	69 ng/µl	Result Flagging Color:	
rRNA Ratio [28s / 18s]:	2.0	Result Flagging Label:	RIN:10

Fragment table for sample 11 : #2-3-1

Name	Start Size [nt]	End Size [nt]	Area	% of total Area
18S	1,522	2,115	51.2	28.0
28S	2,586	3,867	102.0	55.8

 © Copyright 2003 - 2015 Agilent Technologies, Inc.

Assay Class: Eukaryote Total RNA Nano
Data Path: C:\...Eukaryote Total RNA Nano_DE54108122_2023-05-03_11-55-39.xad

Created: 5/3/2023 11:55:38 AM
Modified: 5/3/2023 12:19:27 PM

Electropherogram Summary Continued ...

#2-3-1

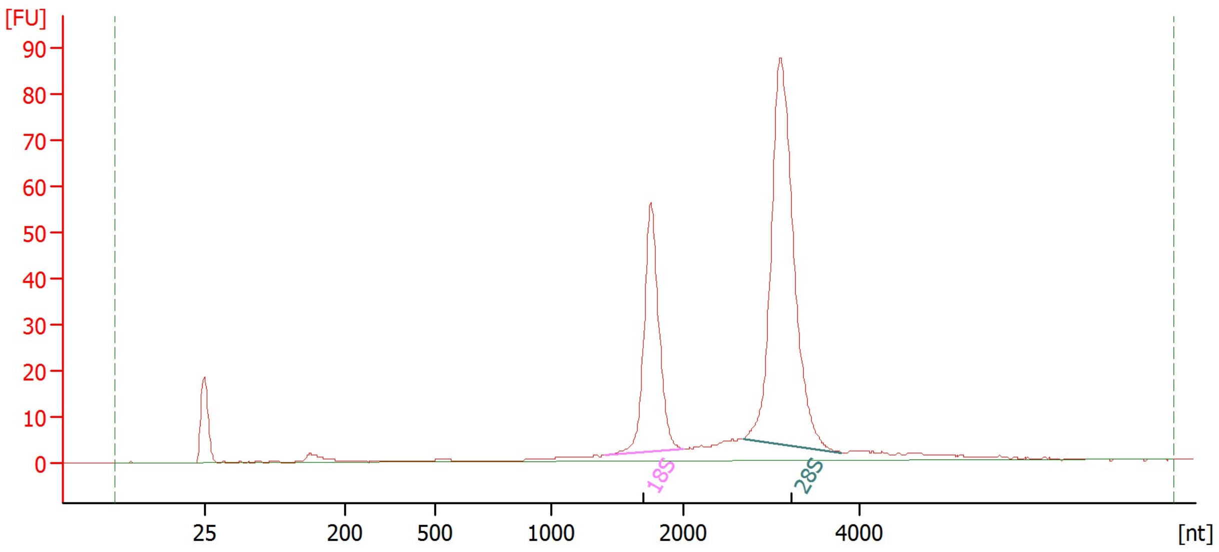

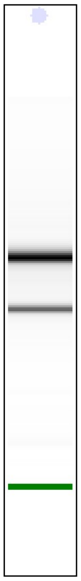

Overall Results for sample 12 : #2-3-1

RNA Area:	395.7	RNA Integrity Number (RIN):	10 (B.02.08)
RNA Concentration:	150 ng/µl	Result Flagging Color:	
rRNA Ratio [28s / 18s]:	2.1	Result Flagging Label:	RIN:10

Fragment table for sample 12 : #2-3-1

Name	Start Size [nt]	End Size [nt]	Area	% of total Area
18S	1,415	2,005	97.4	24.6
28S	2,684	3,801	202.2	51.1

 © Copyright 2003 - 2015 Agilent Technologies, Inc.

Assay Class: Eukaryote Total RNA Nano
Data Path: C:\...Eukaryote Total RNA Nano_DE54108122_2023-05-03_11-55-39.xad
Created: 5/3/2023 11:55:38 AM
Modified: 5/3/2023 12:19:27 PM

Gel Image

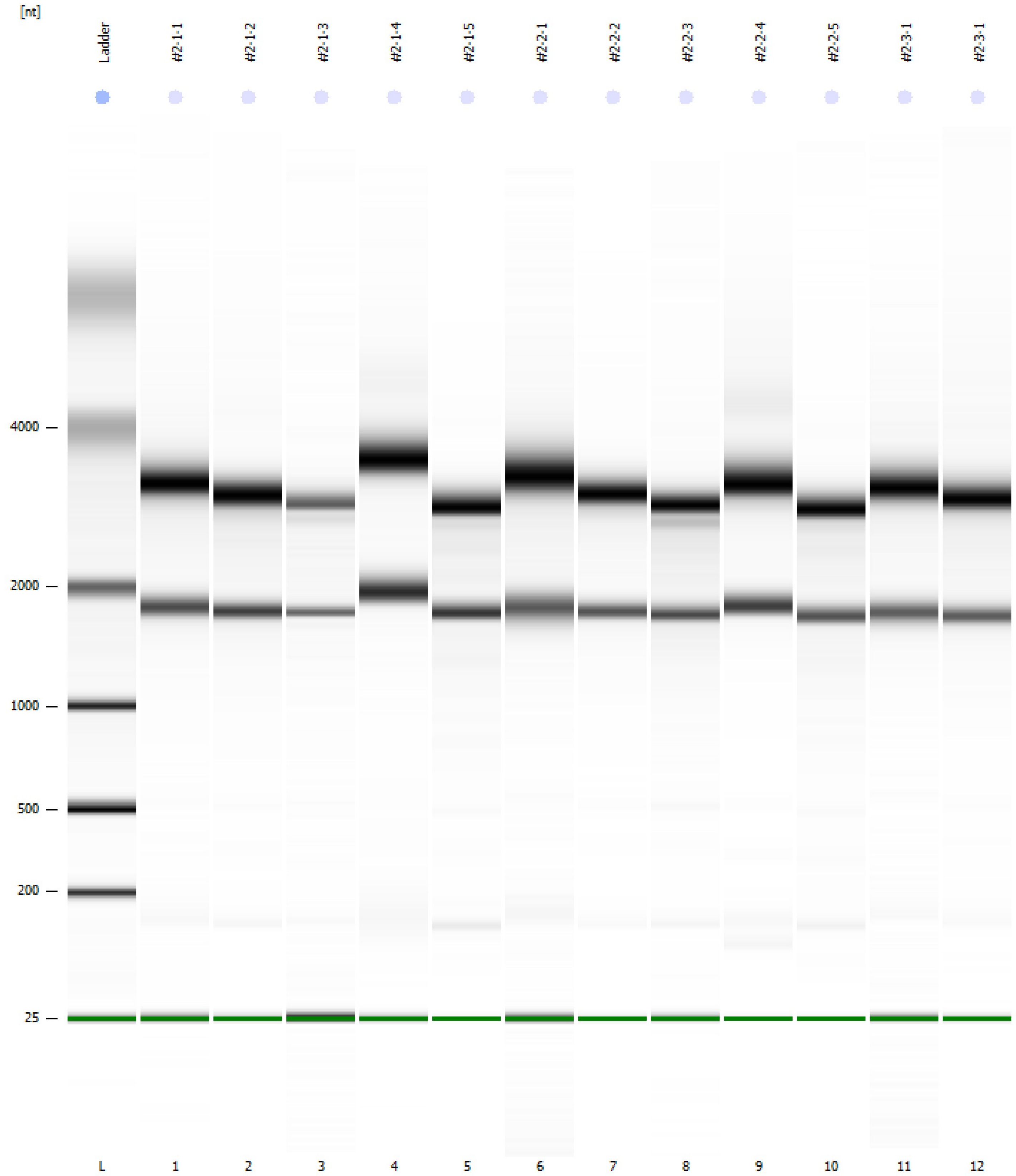

 © Copyright 2003 - 2015 Agilent Technologies, Inc. Printed: 5/3/2023 3:02:25 PM

Assay Class:	Eukaryote Total RNA Nano	Created:	5/3/2023 1:45:34 PM
Data Path:	C:\...Eukaryote Total RNA Nano_DE54108122_2023-05-03_13-45-35.xad	Modified:	5/3/2023 2:08:37 PM

Electrophoresis File Run Summary

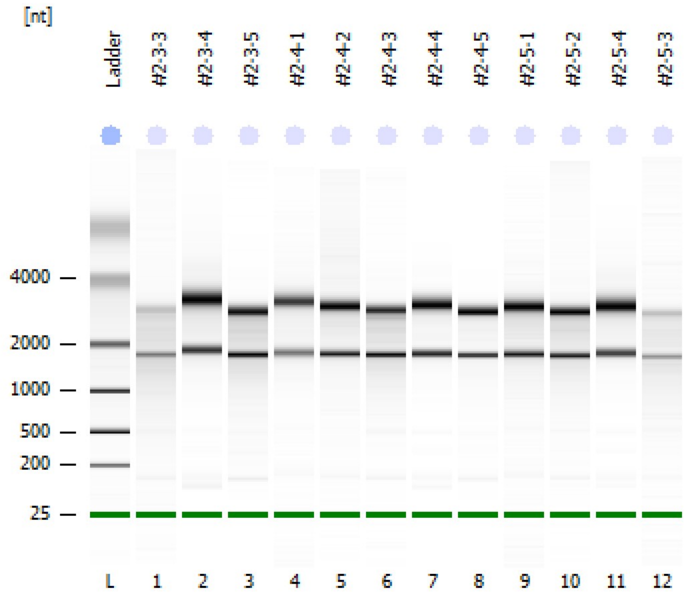

Instrument Information:

Instrument Name:	DE54108122	Firmware:	C.01.069
Serial#:	DE54108122	Type:	G2939A

Assay Information:

Assay Origin Path:	C:\Program Files (x86)\Agilent\2100 bioanalyzer\2100 expert\assays\RNA\Eukaryote Total RNA Nano Series II.xsy
Assay Class:	Eukaryote Total RNA Nano
Version:	2.6
Assay Comments:	Total RNA Analysis ng sensitivity (Eukaryote)

© Copyright 2003 - 2009 Agilent Technologies, Inc.

Chip Information:

Chip Lot #:

Reagent Kit Lot #:

Chip Comments:

#2-3-3

RIN: 7.50

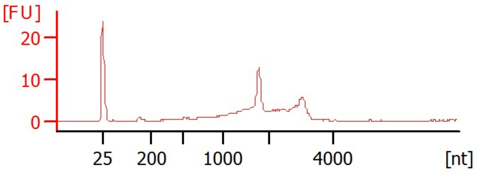

#2-3-4

RIN:10

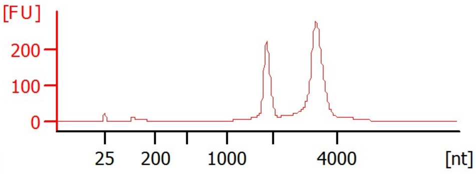

#2-3-5

RIN: 8.30

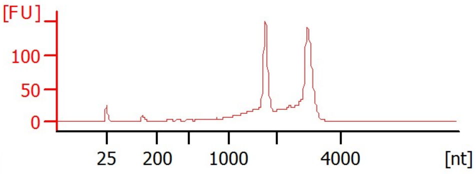

#2-4-1

RIN:10

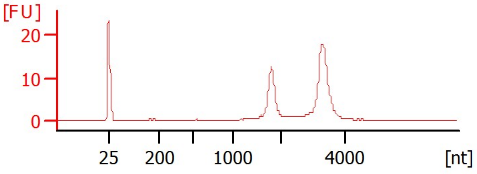

#2-4-2

RIN:10

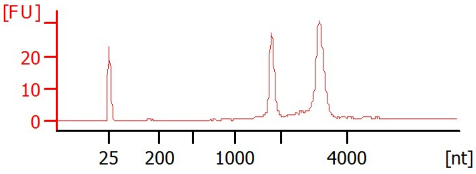

#2-4-3

RIN: 9.30

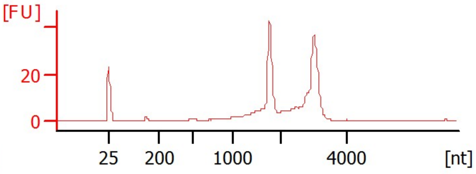

#2-4-4

RIN:10

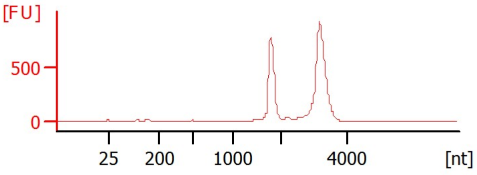

#2-4-5

RIN: 9.90

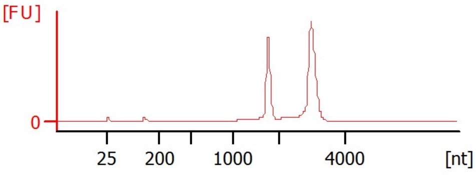

#2-5-1

RIN:10

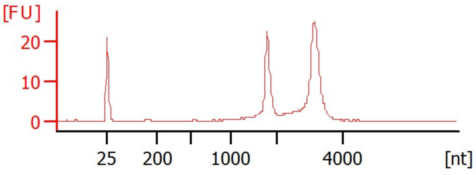

#2-5-2

RIN: 9.90

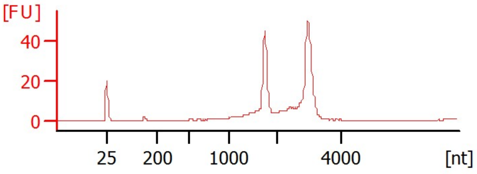

#2-5-4

RIN:10

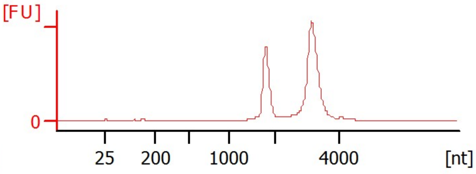

#2-5-3

RIN: 7.50

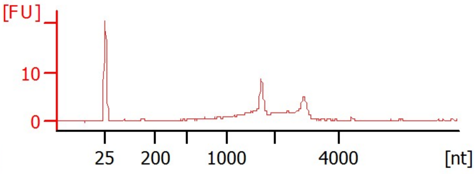

Assay Class: Eukaryote Total RNA Nano Created: 5/3/2023 1:45:34 PM
Data Path: C:\...Eukaryote Total RNA Nano_DE54108122_2023-05-03_13-45-35.xad Modified: 5/3/2023 2:08:37 PM

Electrophoresis File Run Summary (Chip Summary)

Sample Name	Sample Comment	Status	Result Label	Result Color
#2-3-3		✔	RIN: 7.50	
#2-3-4		✔	RIN:10	
#2-3-5		✔	RIN: 8.30	
#2-4-1		✔	RIN:10	
#2-4-2		✔	RIN:10	
#2-4-3		✔	RIN: 9.30	
#2-4-4		✔	RIN:10	
#2-4-5		✔	RIN: 9.90	
#2-5-1		✔	RIN:10	
#2-5-2		✔	RIN: 9.90	
#2-5-4		✔	RIN:10	
#2-5-3		✔	RIN: 7.50	
Ladder		✔	All Other Samples	

Chip Lot # **Reagent Kit Lot #**

Chip Comments :

 © Copyright 2003 - 2015 Agilent Technologies, Inc.

Assay Class: Eukaryote Total RNA Nano
Data Path: C:\...Eukaryote Total RNA Nano_DE54108122_2023-05-03_13-45-35.xad
Created: 5/3/2023 1:45:34 PM
Modified: 5/3/2023 2:08:37 PM

Electrophoresis Assay Details

General Analysis Settings

Number of Available Sample and Ladder Wells (Max.) : 13
Minimum Visible Range [s] : 17
Maximum Visible Range [s] : 70
Start Analysis Time Range [s] : 19
End Analysis Time Range [s] : 69
Ladder Concentration [ng/µl] : 150
Lower Marker Concentration [ng/µl] : 0
Upper Marker Concentration [ng/µl] : 0
Used Lower Marker for Quantitation
Standard Curve Fit is Logarithmic
Show Data Aligned to Lower Marker

Integrator Settings

Integration Start Time [s] : 19
Integration End Time [s] : 69
Slope Threshold : 0.6
Height Threshold [FU] : 0.5
Area Threshold : 0.2
Width Threshold [s] : 0.5
Baseline Plateau [s] : 6

Filter Settings

Filter Width [s] : 0.5
Polynomial Order : 4

Ladder

Ladder Peak	Size
1	25
2	200
3	500
4	1000
5	2000
6	4000

 © Copyright 2003 - 2015 Agilent Technologies, Inc.

Assay Class: Eukaryote Total RNA Nano
Data Path: C:\...Eukaryote Total RNA Nano_DE54108122_2023-05-03_13-45-35.xad
Created: 5/3/2023 1:45:34 PM
Modified: 5/3/2023 2:08:37 PM

Electropherogram Summary

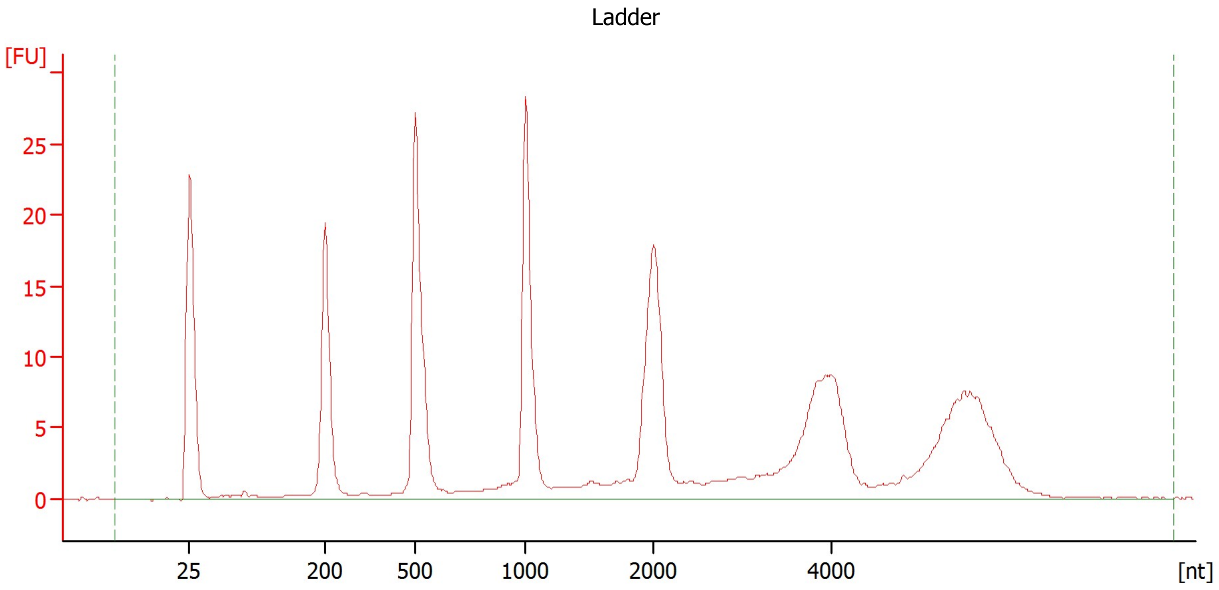

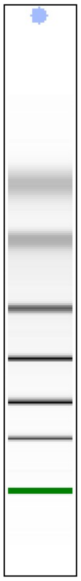

Overall Results for Ladder

RNA Area:	264.9	Result Flagging Color:	
RNA Concentration:	150 ng/µl	Result Flagging Label:	All Other Samples

 © Copyright 2003 - 2015 Agilent Technologies, Inc.

Assay Class:	Eukaryote Total RNA Nano	Created:	5/3/2023 1:45:34 PM
Data Path:	C:\...Eukaryote Total RNA Nano_DE54108122_2023-05-03_13-45-35.xad	Modified:	5/3/2023 2:08:37 PM

Electropherogram Summary Continued ...

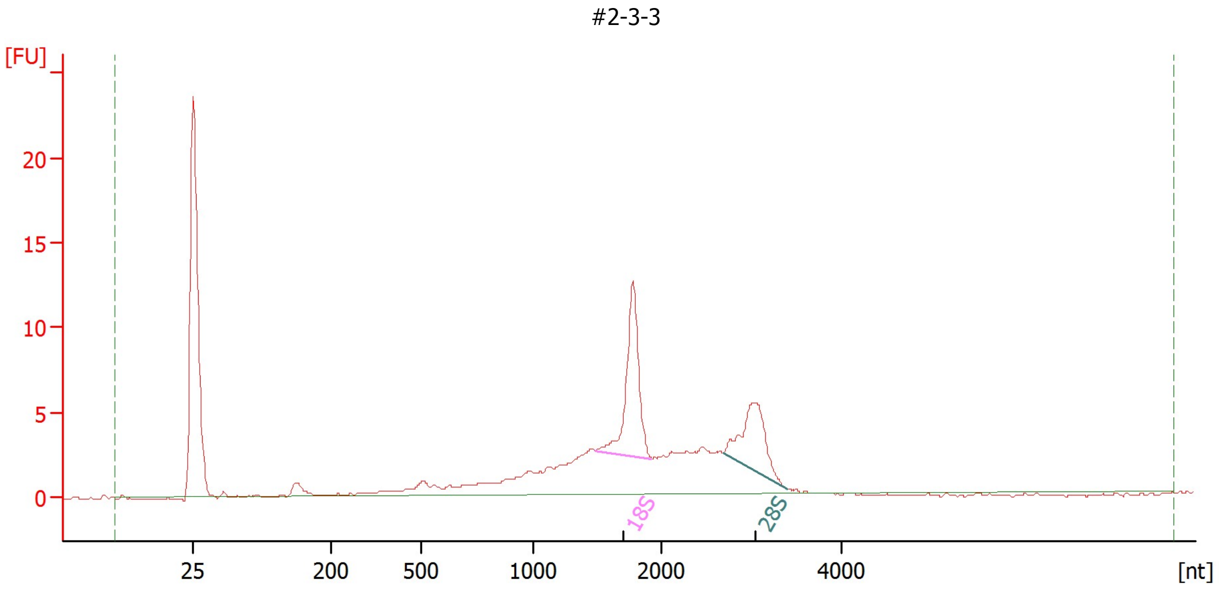

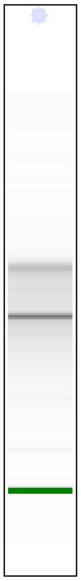

Overall Results for sample 1 : #2-3-3

RNA Area:	98.9	RNA Integrity Number (RIN):	7.5 (B.02.08)
RNA Concentration:	56 ng/µl	Result Flagging Color:	
rRNA Ratio [28s / 18s]:	0.7	Result Flagging Label:	RIN: 7.50

Fragment table for sample 1 : #2-3-3

Name	Start Size [nt]	End Size [nt]	Area	% of total Area
18S	1,489	1,919	15.6	15.8
28S	2,683	3,388	10.3	10.4

 © Copyright 2003 - 2015 Agilent Technologies, Inc.

Assay Class:	Eukaryote Total RNA Nano	Created:	5/3/2023 1:45:34 PM
Data Path:	C:\...Eukaryote Total RNA Nano_DE54108122_2023-05-03_13-45-35.xad	Modified:	5/3/2023 2:08:37 PM

Electropherogram Summary Continued ...

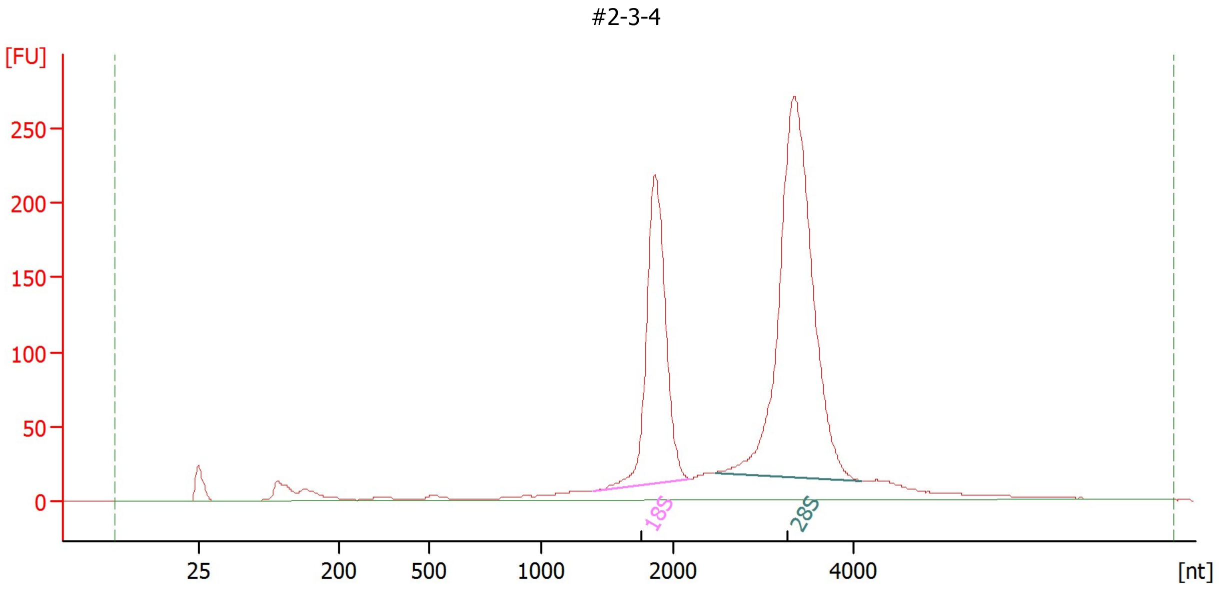

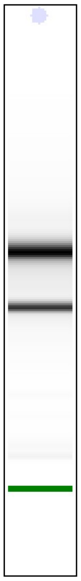

Overall Results for sample 2 : #2-3-4

RNA Area:	1,994.2	RNA Integrity Number (RIN):	10 (B.02.08)
RNA Concentration:	1,129 ng/µl	Result Flagging Color:	
rRNA Ratio [28s / 18s]:	1.9	Result Flagging Label:	RIN:10

Fragment table for sample 2 : #2-3-4

Name	Start Size [nt]	End Size [nt]	Area	% of total Area
18S	1,390	2,194	481.2	24.1
28S	2,478	4,097	902.4	45.3

 © Copyright 2003 - 2015 Agilent Technologies, Inc.

Assay Class:	Eukaryote Total RNA Nano	Created:	5/3/2023 1:45:34 PM
Data Path:	C:\...Eukaryote Total RNA Nano_DE54108122_2023-05-03_13-45-35.xad	Modified:	5/3/2023 2:08:37 PM

Electropherogram Summary Continued ...

#2-3-5

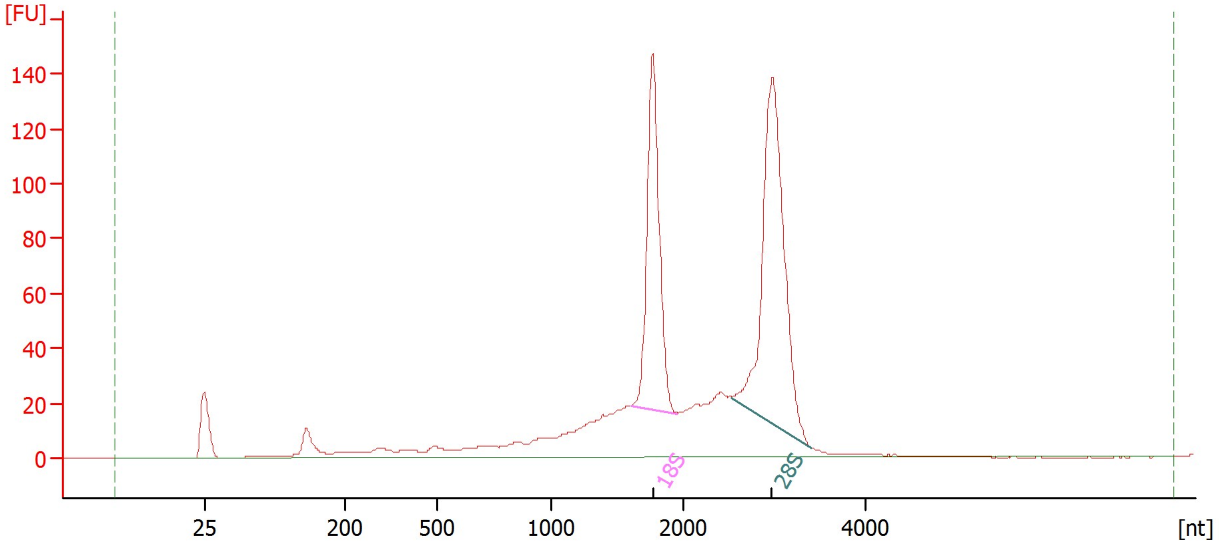

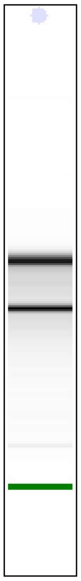

Overall Results for sample 3 : #2-3-5

RNA Area:	1,024.0	RNA Integrity Number (RIN):	8.3 (B.02.08)
RNA Concentration:	580 ng/µl	Result Flagging Color:	
rRNA Ratio [28s / 18s]:	1.5	Result Flagging Label:	RIN: 8.30

Fragment table for sample 3 : #2-3-5

Name	Start Size [nt]	End Size [nt]	Area	% of total Area
18S	1,601	1,957	192.4	18.8
28S	2,534	3,411	290.3	28.4

 © Copyright 2003 - 2015 Agilent Technologies, Inc.

Assay Class: Eukaryote Total RNA Nano
Data Path: C:\...Eukaryote Total RNA Nano_DE54108122_2023-05-03_13-45-35.xad
Created: 5/3/2023 1:45:34 PM
Modified: 5/3/2023 2:08:37 PM

Electropherogram Summary Continued ...

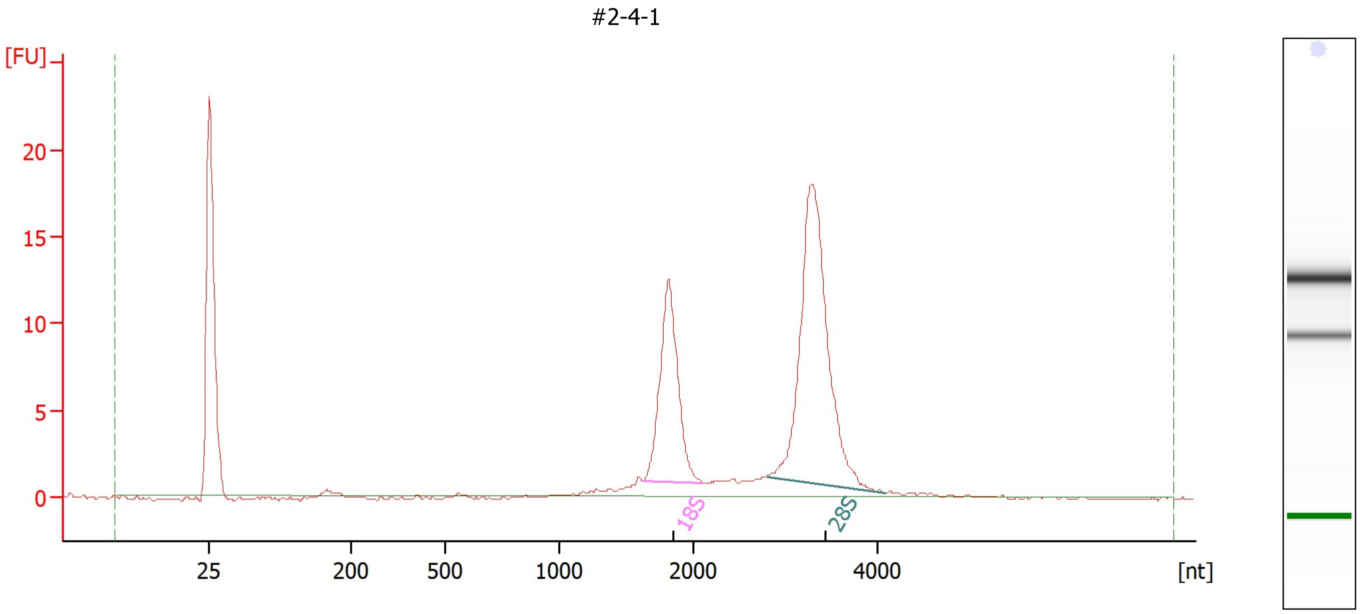

Overall Results for sample 4 : #2-4-1

RNA Area:	97.5	RNA Integrity Number (RIN):	10 (B.02.08)
RNA Concentration:	55 ng/µl	Result Flagging Color:	
rRNA Ratio [28s / 18s]:	2.0	Result Flagging Label:	RIN:10

Fragment table for sample 4 : #2-4-1

Name	Start Size [nt]	End Size [nt]	Area	% of total Area
18S	1,623	2,115	24.7	25.4
28S	2,798	4,070	48.6	49.9

 © Copyright 2003 - 2015 Agilent Technologies, Inc.

Assay Class:	Eukaryote Total RNA Nano	Created:	5/3/2023 1:45:34 PM
Data Path:	C:\...Eukaryote Total RNA Nano_DE54108122_2023-05-03_13-45-35.xad	Modified:	5/3/2023 2:08:37 PM

Electropherogram Summary Continued ...

#2-4-2

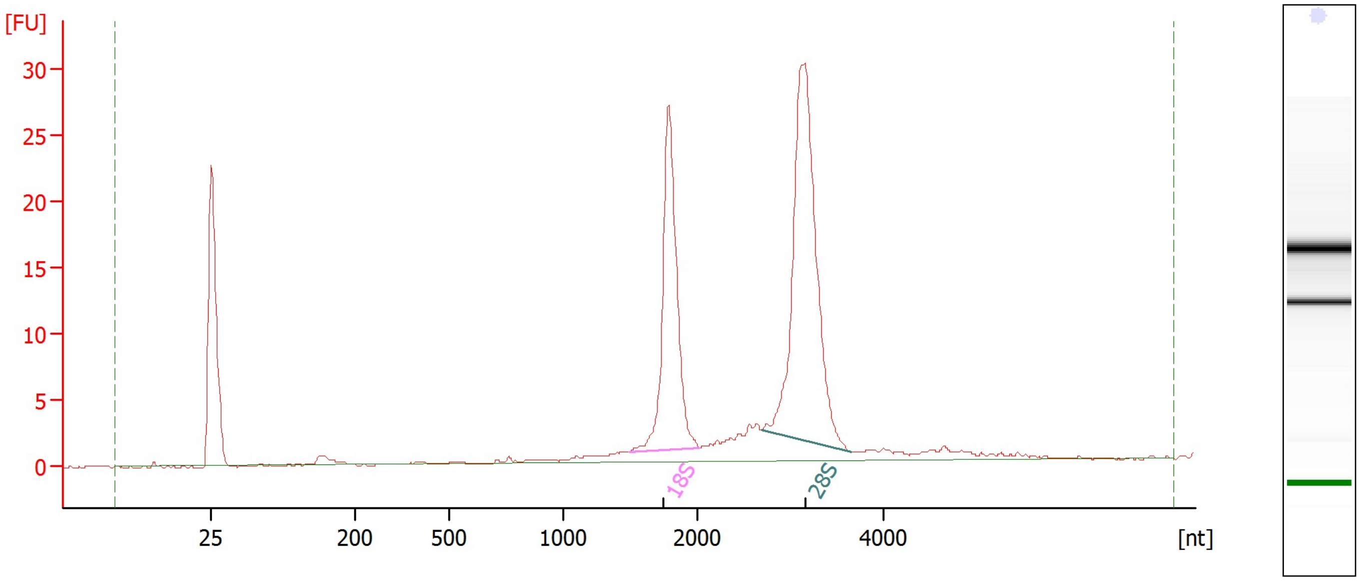

Overall Results for sample 5 : #2-4-2

RNA Area:	163.2	RNA Integrity Number (RIN):	10 (B.02.08)
RNA Concentration:	92 ng/µl	Result Flagging Color:	
rRNA Ratio [28s / 18s]:	1.6	Result Flagging Label:	RIN:10

Fragment table for sample 5 : #2-4-2

Name	Start Size [nt]	End Size [nt]	Area	% of total Area
18S	1,490	2,024	43.7	26.8
28S	2,680	3,660	69.0	42.3

Assay Class: Eukaryote Total RNA Nano
Data Path: C:\...Eukaryote Total RNA Nano_DE54108122_2023-05-03_13-45-35.xad
Created: 5/3/2023 1:45:34 PM
Modified: 5/3/2023 2:08:37 PM

Electropherogram Summary Continued ...

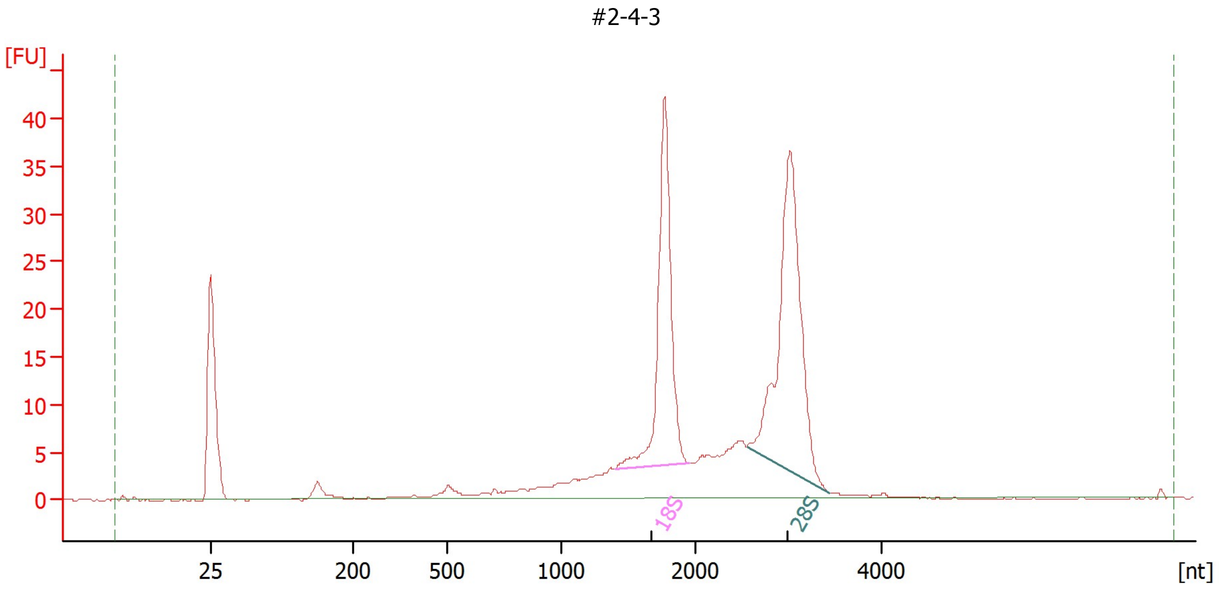

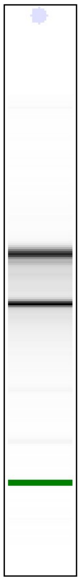

Overall Results for sample 6 : #2-4-3

RNA Area:	240.3	RNA Integrity Number (RIN):	9.3 (B.02.08)
RNA Concentration:	136 ng/µl	Result Flagging Color:	
rRNA Ratio [28s / 18s]:	1.3	Result Flagging Label:	RIN: 9.30

Fragment table for sample 6 : #2-4-3

Name	Start Size [nt]	End Size [nt]	Area	% of total Area
18S	1,393	1,960	58.4	24.3
28S	2,554	3,443	76.3	31.7

 © Copyright 2003 - 2015 Agilent Technologies, Inc.

Assay Class: Eukaryote Total RNA Nano
Data Path: C:\...Eukaryote Total RNA Nano_DE54108122_2023-05-03_13-45-35.xad

Created: 5/3/2023 1:45:34 PM
Modified: 5/3/2023 2:08:37 PM

Electropherogram Summary Continued ...

#2-4-4

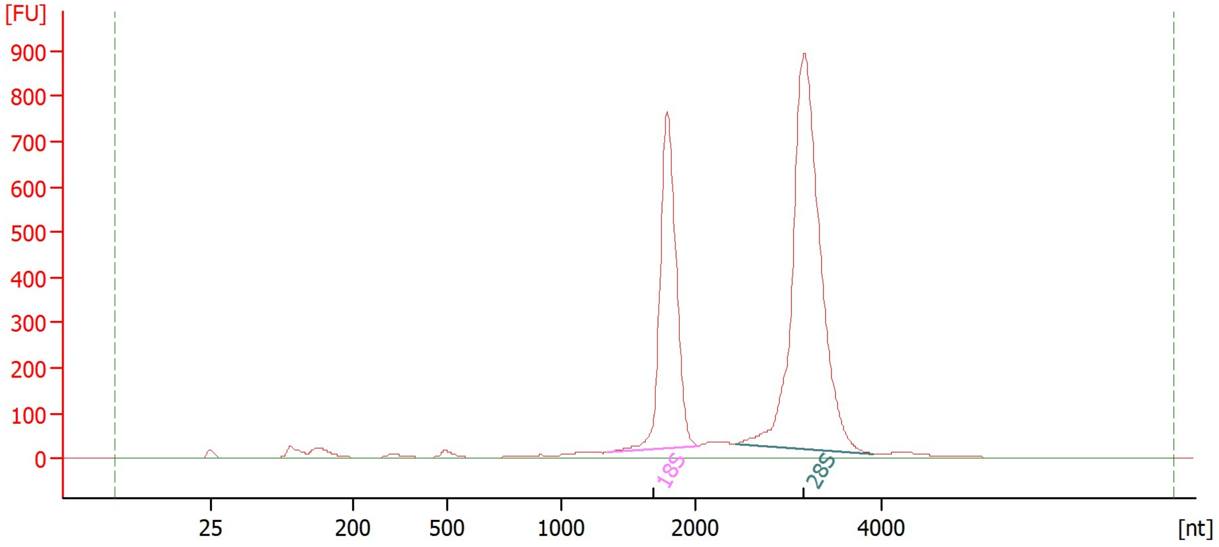

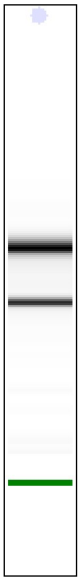

Overall Results for sample 7 : #2-4-4

RNA Area:	4,977.8	RNA Integrity Number (RIN):	10 (B.02.08)
RNA Concentration:	2,818 ng/µl	Result Flagging Color:	
rRNA Ratio [28s / 18s]:	1.8	Result Flagging Label:	RIN:10

Fragment table for sample 7 : #2-4-4

Name	Start Size [nt]	End Size [nt]	Area	% of total Area
18S	1,337	2,035	1,429.5	28.7
28S	2,427	3,917	2,516.4	50.6

 © Copyright 2003 - 2015 Agilent Technologies, Inc.

Assay Class: Eukaryote Total RNA Nano Created: 5/3/2023 1:45:34 PM
Data Path: C:\...Eukaryote Total RNA Nano_DE54108122_2023-05-03_13-45-35.xad Modified: 5/3/2023 2:08:37 PM

Electropherogram Summary Continued ...

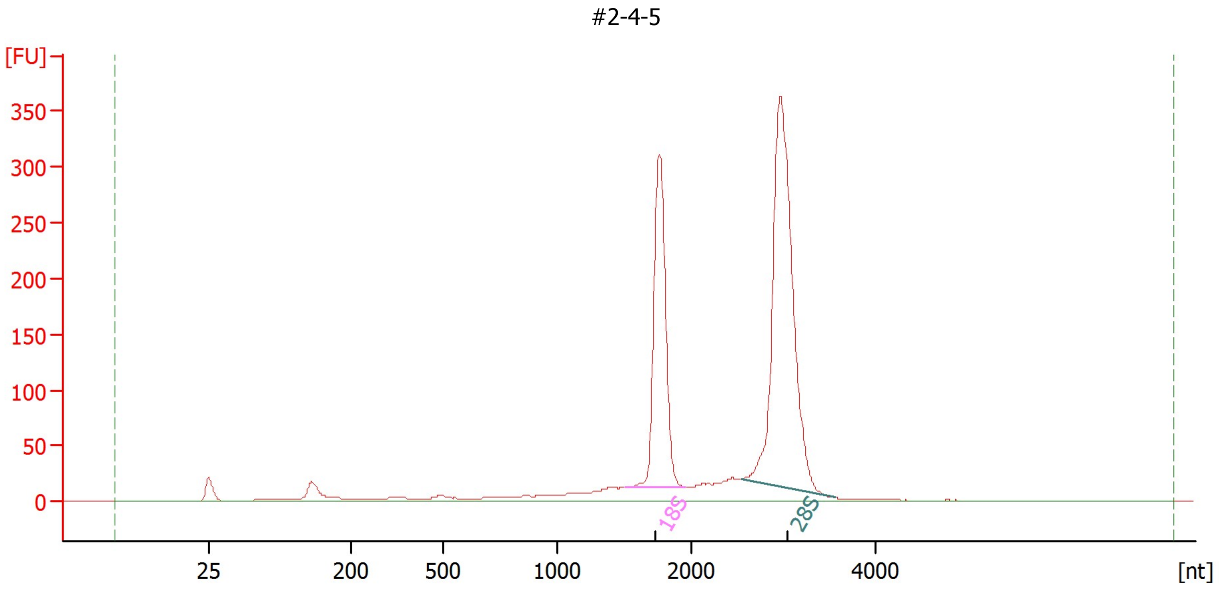

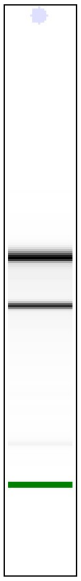

Overall Results for sample 8 : #2-4-5

RNA Area:	1,674.0	RNA Integrity Number (RIN):	9.9 (B.02.08)
RNA Concentration:	948 ng/µl	Result Flagging Color:	
rRNA Ratio [28s / 18s]:	1.8	Result Flagging Label:	RIN: 9.90

Fragment table for sample 8 : #2-4-5

Name	Start Size [nt]	End Size [nt]	Area	% of total Area
18S	1,510	1,959	418.5	25.0
28S	2,532	3,552	751.0	44.9

 © Copyright 2003 - 2015 Agilent Technologies, Inc.

Assay Class:	Eukaryote Total RNA Nano	Created:	5/3/2023 1:45:34 PM
Data Path:	C:\...Eukaryote Total RNA Nano_DE54108122_2023-05-03_13-45-35.xad	Modified:	5/3/2023 2:08:37 PM

Electropherogram Summary Continued ...

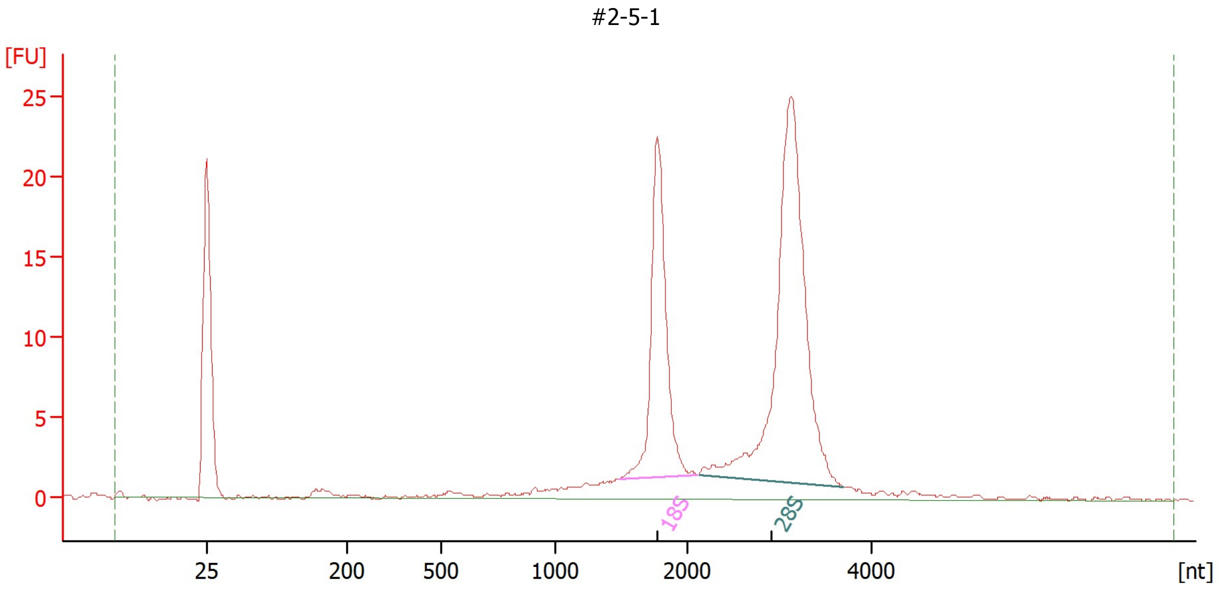

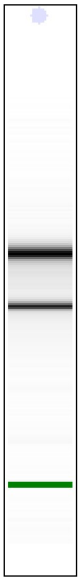

Overall Results for sample 9 : #2-5-1

RNA Area:	156.3	RNA Integrity Number (RIN):	10 (B.02.08)
RNA Concentration:	88 ng/µl	Result Flagging Color:	
rRNA Ratio [28s / 18s]:	1.9	Result Flagging Label:	RIN:10

Fragment table for sample 9 : #2-5-1

Name	Start Size [nt]	End Size [nt]	Area	% of total Area
18S	1,483	2,102	37.5	24.0
28S	2,126	3,709	71.8	45.9

 © Copyright 2003 - 2015 Agilent Technologies, Inc.

Assay Class:	Eukaryote Total RNA Nano	Created:	5/3/2023 1:45:34 PM
Data Path:	C:\...Eukaryote Total RNA Nano_DE54108122_2023-05-03_13-45-35.xad	Modified:	5/3/2023 2:08:37 PM

Electropherogram Summary Continued ...

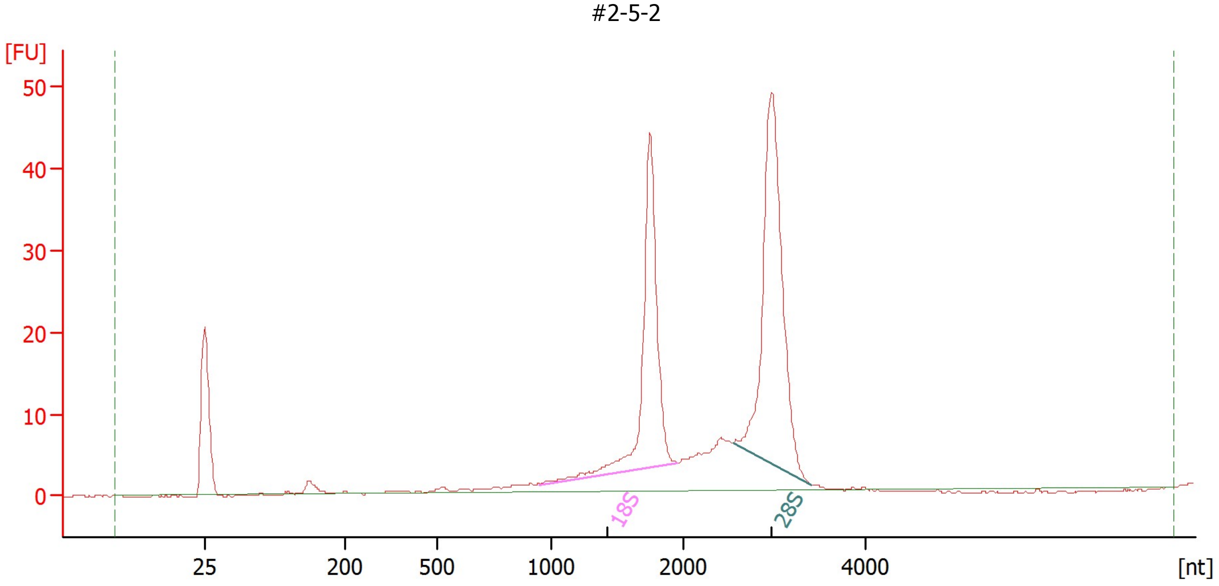

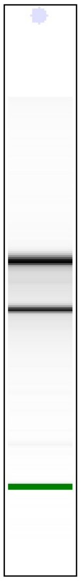

Overall Results for sample 10 : #2-5-2

RNA Area:	256.2	RNA Integrity Number (RIN):	9.9 (B.02.08)
RNA Concentration:	145 ng/µl	Result Flagging Color:	
rRNA Ratio [28s / 18s]:	1.4	Result Flagging Label:	RIN: 9.90

Fragment table for sample 10 : #2-5-2

Name	Start Size [nt]	End Size [nt]	Area	% of total Area
18S	950	1,957	68.1	26.6
28S	2,546	3,411	94.2	36.8

 © Copyright 2003 - 2015 Agilent Technologies, Inc.

Assay Class: Eukaryote Total RNA Nano
Data Path: C:\...Eukaryote Total RNA Nano_DE54108122_2023-05-03_13-45-35.xad
Created: 5/3/2023 1:45:34 PM
Modified: 5/3/2023 2:08:37 PM

Electropherogram Summary Continued ...

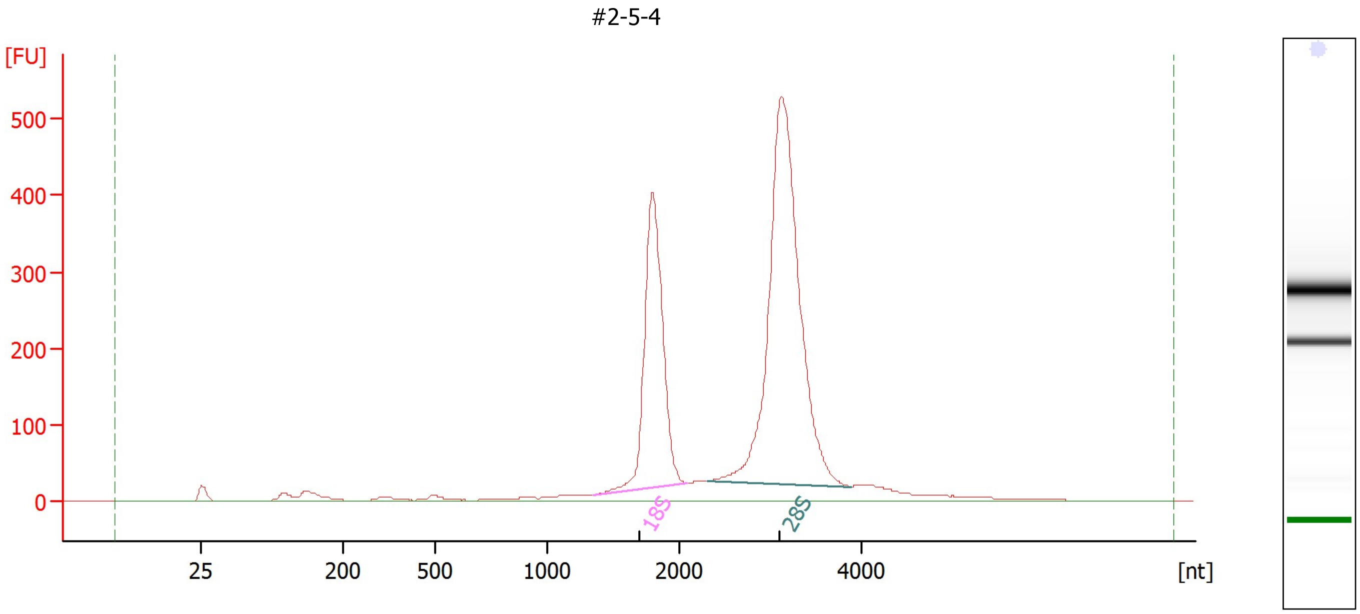

Overall Results for sample 11 : #2-5-4

RNA Area:	3,344.3	RNA Integrity Number (RIN):	10 (B.02.08)
RNA Concentration:	1,894 ng/µl	Result Flagging Color:	
rRNA Ratio [28s / 18s]:	1.9	Result Flagging Label:	RIN:10

Fragment table for sample 11 : #2-5-4

Name	Start Size [nt]	End Size [nt]	Area	% of total Area
18S	1,355	2,089	852.2	25.5
28S	2,312	3,885	1,635.5	48.9

 © Copyright 2003 - 2015 Agilent Technologies, Inc.

Assay Class: Eukaryote Total RNA Nano
Data Path: C:\...Eukaryote Total RNA Nano_DE54108122_2023-05-03_13-45-35.xad
Created: 5/3/2023 1:45:34 PM
Modified: 5/3/2023 2:08:37 PM

Electropherogram Summary Continued ...

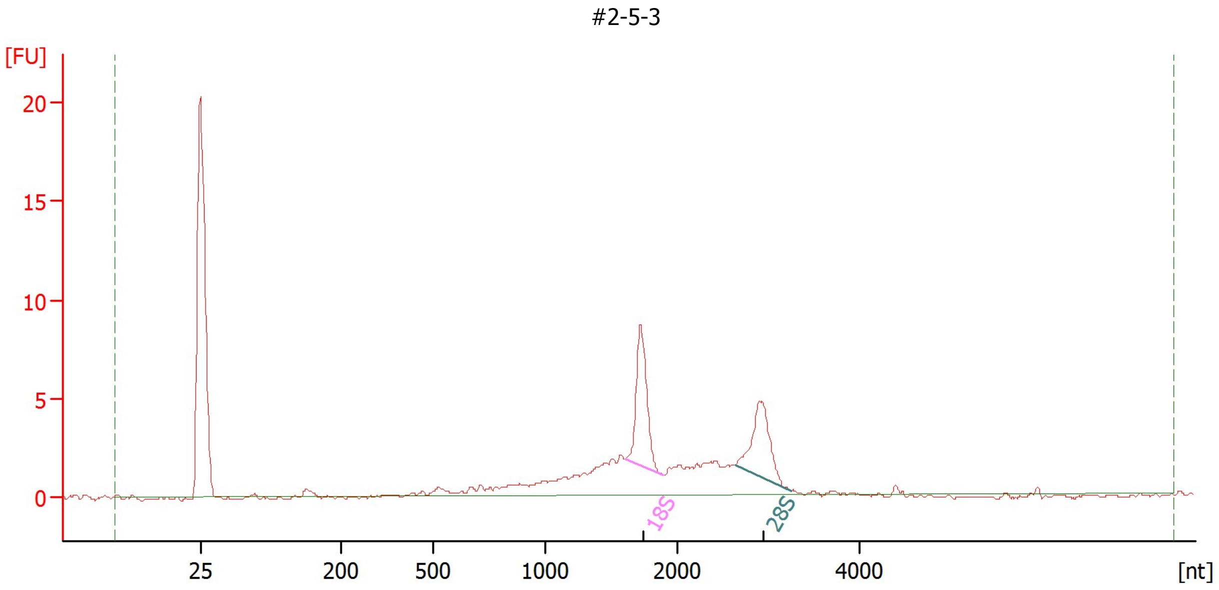

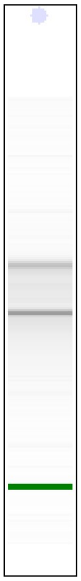

Overall Results for sample 12 : #2-5-3

RNA Area:	54.9	RNA Integrity Number (RIN):	7.5 (B.02.08)
RNA Concentration:	31 ng/µl	Result Flagging Color:	
rRNA Ratio [28s / 18s]:	0.8	Result Flagging Label:	RIN: 7.50

Fragment table for sample 12 : #2-5-3

Name	Start Size [nt]	End Size [nt]	Area	% of total Area
18S	1,605	1,890	9.2	16.8
28S	2,653	3,253	7.3	13.4

 © Copyright 2003 - 2015 Agilent Technologies, Inc.

Assay Class: Eukaryote Total RNA Nano
Data Path: C:\...Eukaryote Total RNA Nano_DE54108122_2023-05-03_13-45-35.xad
Created: 5/3/2023 1:45:34 PM
Modified: 5/3/2023 2:08:37 PM

Gel Image

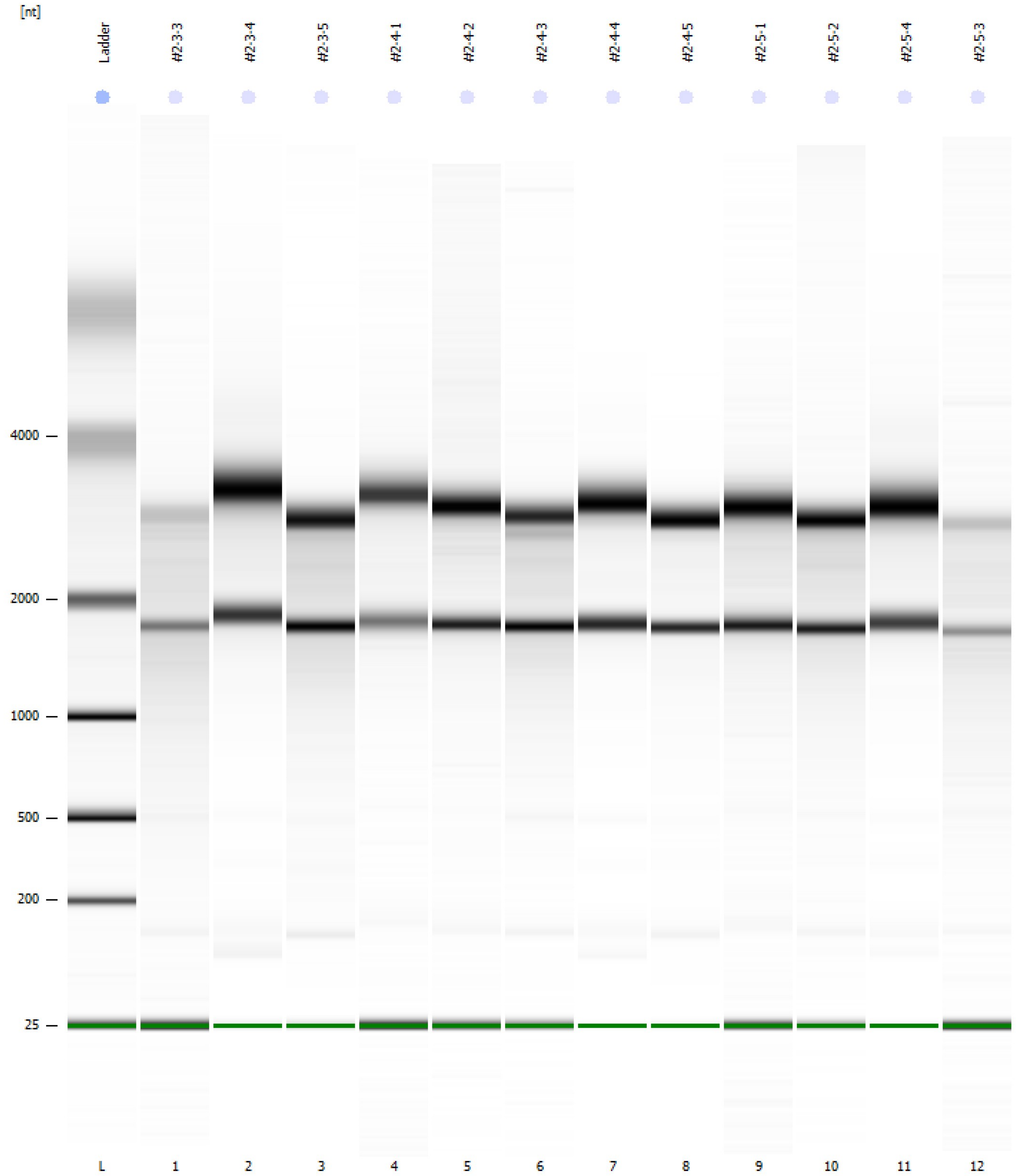

 © Copyright 2003 - 2015 Agilent Technologies, Inc. Printed: 5/3/2023 3:02:03 PM

Assay Class:	Eukaryote Total RNA Nano	Created:	5/3/2023 2:29:24 PM
Data Path:	C:\...Eukaryote Total RNA Nano_DE54108122_2023-05-03_14-29-24.xad	Modified:	5/3/2023 2:52:27 PM

Electrophoresis File Run Summary

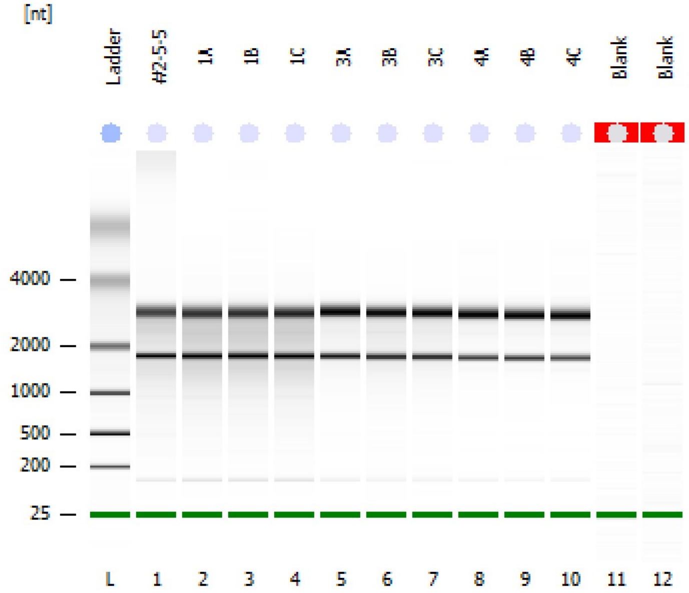

Instrument Information:

Instrument Name:	DE54108122	Firmware:	C.01.069
Serial#:	DE54108122	Type:	G2939A

Assay Information:

Assay Origin Path:	C:\Program Files (x86)\Agilent\2100 bioanalyzer\2100 expert\assays\RNA\Eukaryote Total RNA Nano Series II.xsy
Assay Class:	Eukaryote Total RNA Nano
Version:	2.6
Assay Comments:	Total RNA Analysis ng sensitivity (Eukaryote)

© Copyright 2003 - 2009 Agilent Technologies, Inc.

Chip Information:

Chip Lot #:

Reagent Kit Lot #:

Chip Comments:

#2-5-5

RIN: 8.40

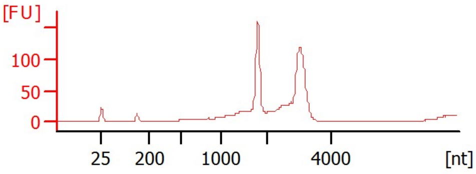

1A

RIN:8

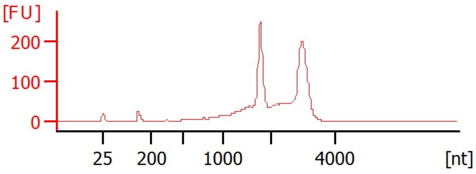

1B

RIN: 8.10

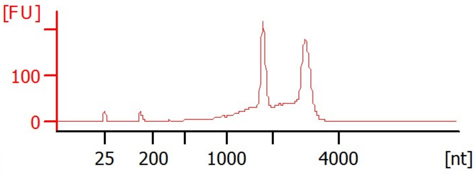

1C

RIN: 8.10

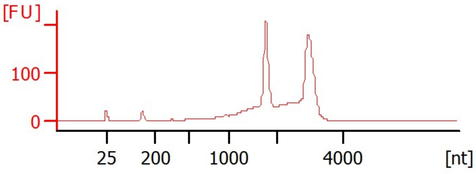

3A

RIN:10

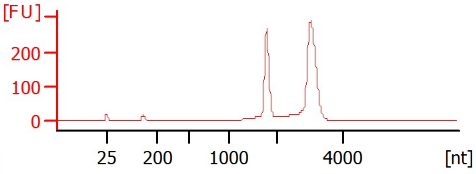

3B

RIN: 9.70

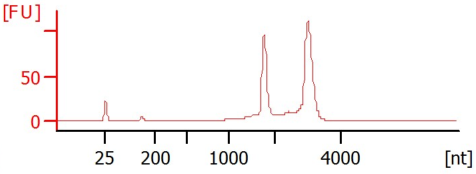

3C

RIN:10

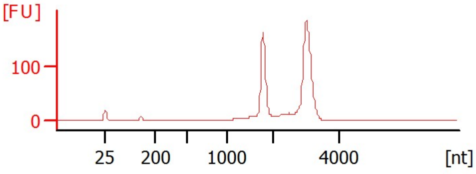

4A

RIN:10

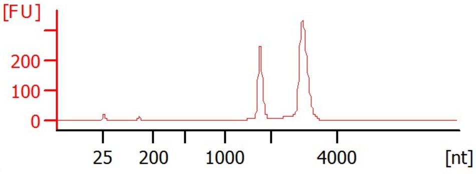

4B

RIN:10

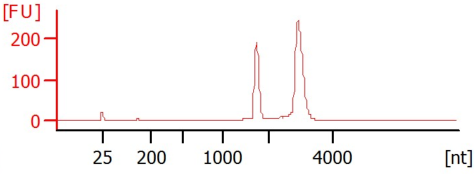

4C

RIN:10

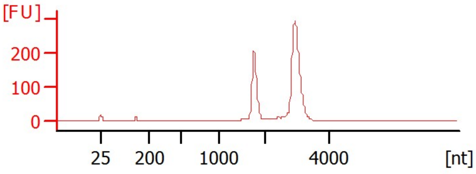

Blank

RIN N/A

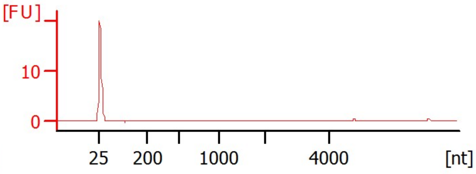

Blank

RIN N/A

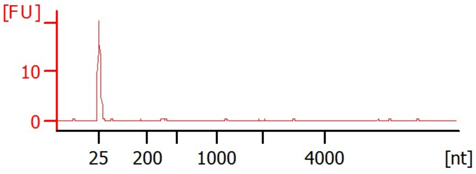

Assay Class: Eukaryote Total RNA Nano
Data Path: C:\...Eukaryote Total RNA Nano_DE54108122_2023-05-03_14-29-24.xad
Created: 5/3/2023 2:29:24 PM
Modified: 5/3/2023 2:52:27 PM

Electrophoresis File Run Summary (Chip Summary)

Sample Name	Sample Comment	Status	Result Label	Result Color
#2-5-5		✓	RIN: 8.40	
1A		✓	RIN:8	
1B		✓	RIN: 8.10	
1C		✓	RIN: 8.10	
3A		✓	RIN:10	
3B		✓	RIN: 9.70	
3C		✓	RIN:10	
4A		✓	RIN:10	
4B		✓	RIN:10	
4C		✓	RIN:10	
Blank		✓	RIN N/A	
Blank		✓	RIN N/A	
Ladder		✓	All Other Samples	

Chip Lot # **Reagent Kit Lot #**

Chip Comments :

 © Copyright 2003 - 2015 Agilent Technologies, Inc.

Assay Class:	Eukaryote Total RNA Nano	Created:	5/3/2023 2:29:24 PM
Data Path:	C:\...Eukaryote Total RNA Nano_DE54108122_2023-05-03_14-29-24.xad	Modified:	5/3/2023 2:52:27 PM

Electrophoresis Assay Details

General Analysis Settings

Number of Available Sample and Ladder Wells (Max.) : 13
Minimum Visible Range [s] : 17
Maximum Visible Range [s] : 70
Start Analysis Time Range [s] : 19
End Analysis Time Range [s] : 69
Ladder Concentration [ng/µl] : 150
Lower Marker Concentration [ng/µl] : 0
Upper Marker Concentration [ng/µl] : 0
Used Lower Marker for Quantitation
Standard Curve Fit is Logarithmic
Show Data Aligned to Lower Marker

Integrator Settings

Integration Start Time [s] : 19
Integration End Time [s] : 69
Slope Threshold : 0.6
Height Threshold [FU] : 0.5
Area Threshold : 0.2
Width Threshold [s] : 0.5
Baseline Plateau [s] : 6

Filter Settings

Filter Width [s] : 0.5
Polynomial Order : 4

Ladder

Ladder Peak	Size
1	25
2	200
3	500
4	1000
5	2000
6	4000

© Copyright 2003 - 2015 Agilent Technologies, Inc.

Assay Class:	Eukaryote Total RNA Nano	Created:	5/3/2023 2:29:24 PM
Data Path:	C:\...Eukaryote Total RNA Nano_DE54108122_2023-05-03_14-29-24.xad	Modified:	5/3/2023 2:52:27 PM

Electropherogram Summary

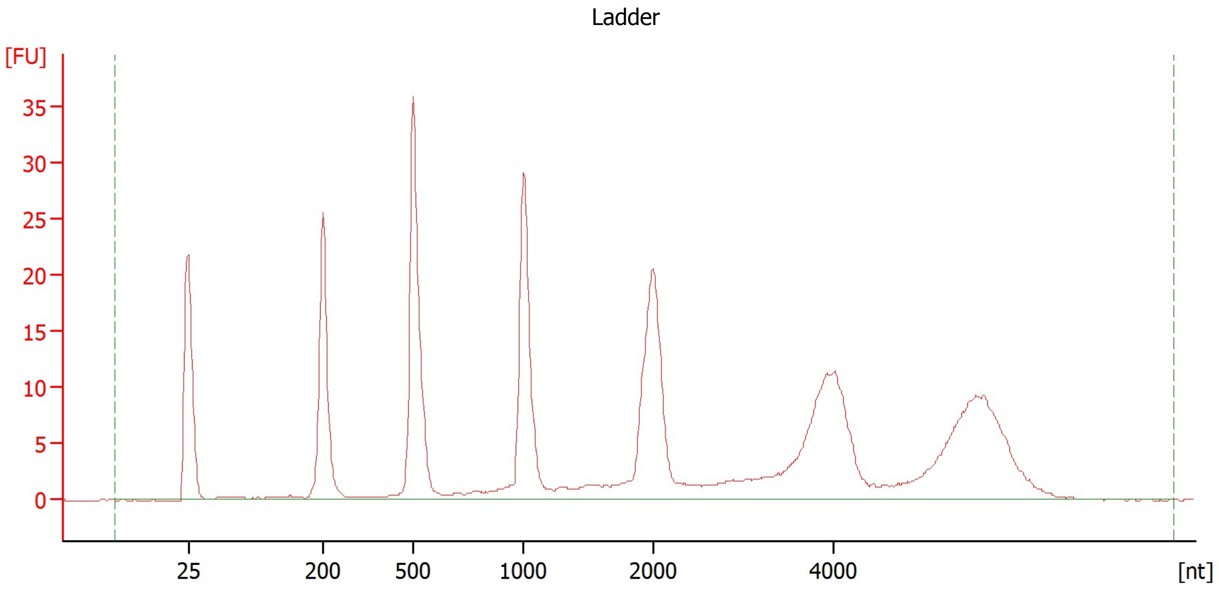

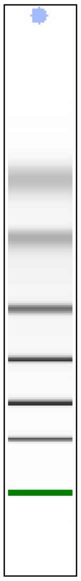

Overall Results for Ladder

RNA Area:	328.0	Result Flagging Color:	
RNA Concentration:	150 ng/µl	Result Flagging Label:	All Other Samples

 © Copyright 2003 - 2015 Agilent Technologies, Inc.

Assay Class: Eukaryote Total RNA Nano
Data Path: C:\...Eukaryote Total RNA Nano_DE54108122_2023-05-03_14-29-24.xad

Created: 5/3/2023 2:29:24 PM
Modified: 5/3/2023 2:52:27 PM

Electropherogram Summary Continued ...

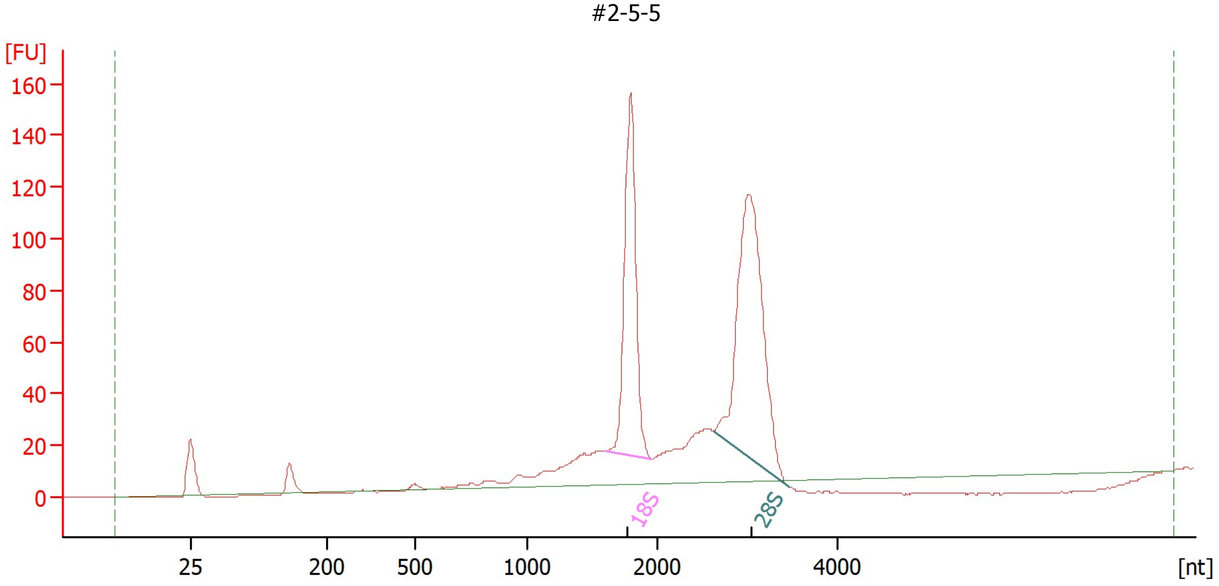

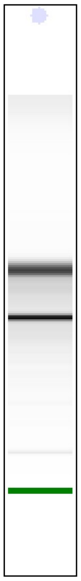

Overall Results for sample 1 : #2-5-5

RNA Area:	815.8	RNA Integrity Number (RIN):	8.4 (B.02.08)
RNA Concentration:	373 ng/µl	Result Flagging Color:	
rRNA Ratio [28s / 18s]:	1.5	Result Flagging Label:	RIN: 8.40

Fragment table for sample 1 : #2-5-5

Name	Start Size [nt]	End Size [nt]	Area	% of total Area
18S	1,597	1,959	196.0	24.0
28S	2,625	3,462	285.6	35.0

© Copyright 2003 - 2015 Agilent Technologies, Inc.

Assay Class:	Eukaryote Total RNA Nano	Created:	5/3/2023 2:29:24 PM
Data Path:	C:\...Eukaryote Total RNA Nano_DE54108122_2023-05-03_14-29-24.xad	Modified:	5/3/2023 2:52:27 PM

Electropherogram Summary Continued ...

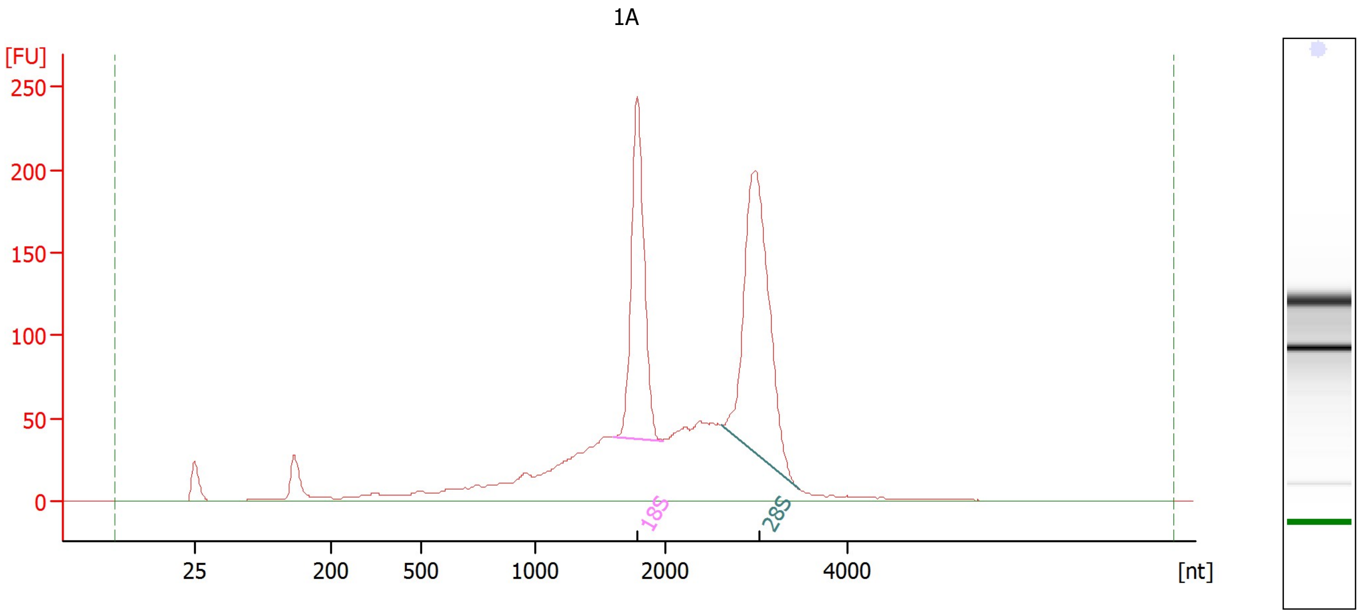

Overall Results for sample 2 : 1A

RNA Area:	1,945.1	RNA Integrity Number (RIN):	8 (B.02.08)
RNA Concentration:	889 ng/µl	Result Flagging Color:	
rRNA Ratio [28s / 18s]:	1.4	Result Flagging Label:	RIN:8

Fragment table for sample 2 : 1A

Name	Start Size [nt]	End Size [nt]	Area	% of total Area
18S	1,602	1,977	324.6	16.7
28S	2,622	3,475	465.5	23.9

 © Copyright 2003 - 2015 Agilent Technologies, Inc.

Assay Class: Eukaryote Total RNA Nano
Data Path: C:\...Eukaryote Total RNA Nano_DE54108122_2023-05-03_14-29-24.xad
Created: 5/3/2023 2:29:24 PM
Modified: 5/3/2023 2:52:27 PM

Electropherogram Summary Continued ...

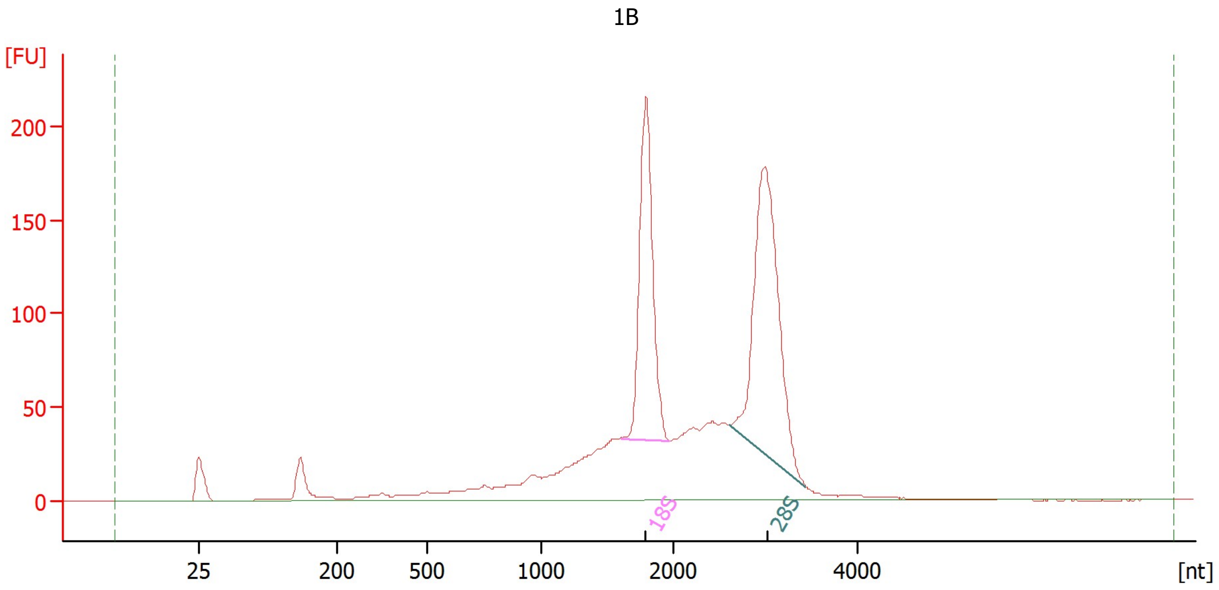

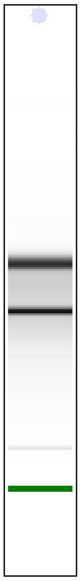

Overall Results for sample 3 : 1B

RNA Area:	1,670.9	RNA Integrity Number (RIN):	8.1 (B.02.08)
RNA Concentration:	764 ng/µl	Result Flagging Color:	
rRNA Ratio [28s / 18s]:	1.4	Result Flagging Label:	RIN: 8.10

Fragment table for sample 3 : 1B

Name	Start Size [nt]	End Size [nt]	Area	% of total Area
18S	1,607	1,971	289.8	17.3
28S	2,596	3,430	410.9	24.6

 © Copyright 2003 - 2015 Agilent Technologies, Inc.

Assay Class: Eukaryote Total RNA Nano
Data Path: C:\...Eukaryote Total RNA Nano_DE54108122_2023-05-03_14-29-24.xad

Created: 5/3/2023 2:29:24 PM
Modified: 5/3/2023 2:52:27 PM

Electropherogram Summary Continued ...

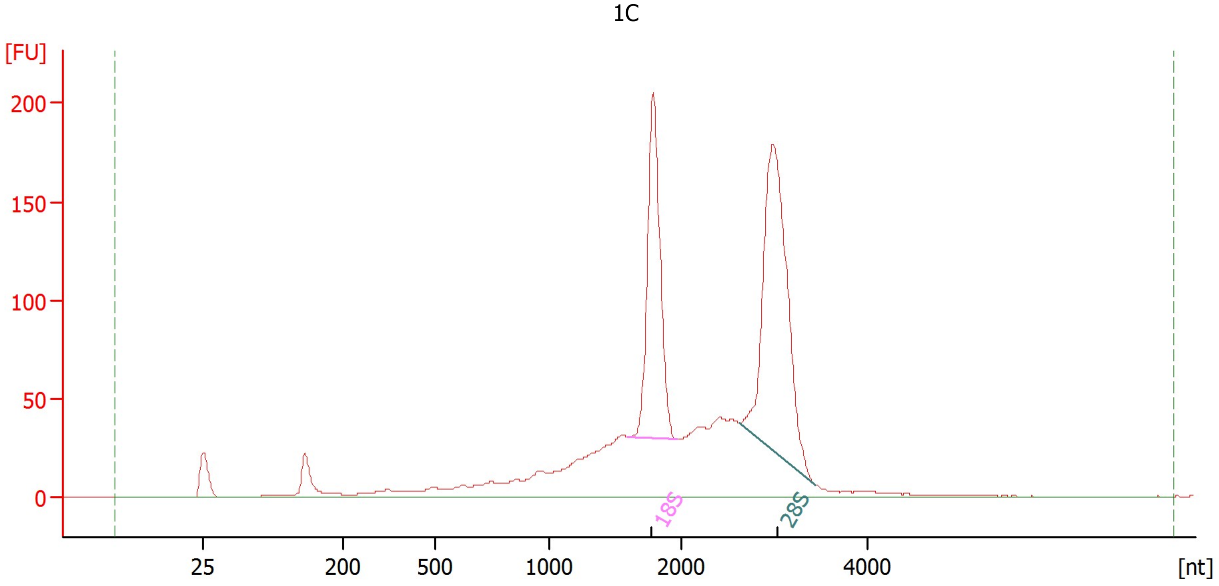

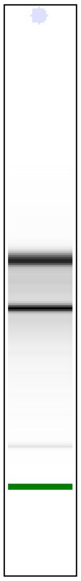

Overall Results for sample 4 : 1C

RNA Area:	1,605.6	RNA Integrity Number (RIN):	8.1 (B.02.08)
RNA Concentration:	734 ng/µl	Result Flagging Color:	
rRNA Ratio [28s / 18s]:	1.5	Result Flagging Label:	RIN: 8.10

Fragment table for sample 4 : 1C

Name	Start Size [nt]	End Size [nt]	Area	% of total Area
18S	1,596	1,957	270.2	16.8
28S	2,616	3,432	402.8	25.1

 © Copyright 2003 - 2015 Agilent Technologies, Inc.

Assay Class: Eukaryote Total RNA Nano
Data Path: C:\...Eukaryote Total RNA Nano_DE54108122_2023-05-03_14-29-24.xad

Created: 5/3/2023 2:29:24 PM
Modified: 5/3/2023 2:52:27 PM

Electropherogram Summary Continued ...

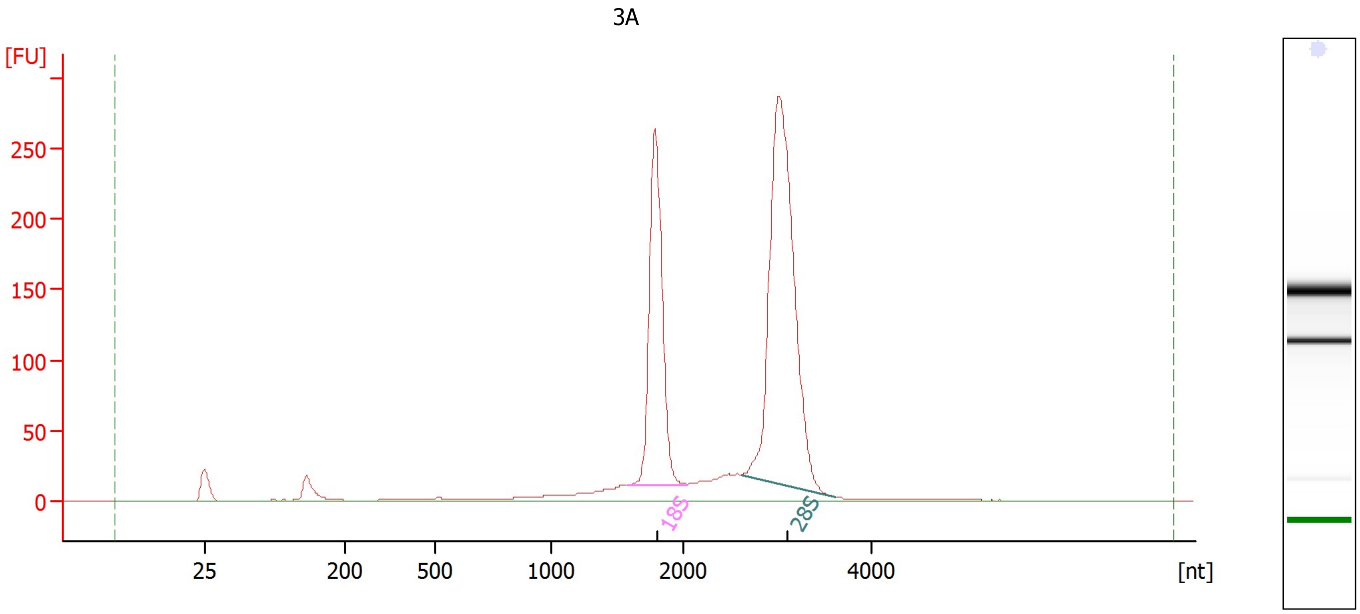

Overall Results for sample 5 : 3A

RNA Area:	1,544.2	RNA Integrity Number (RIN):	10 (B.02.08)
RNA Concentration:	706 ng/µl	Result Flagging Color:	
rRNA Ratio [28s / 18s]:	1.8	Result Flagging Label:	RIN:10

Fragment table for sample 5 : 3A

Name	Start Size [nt]	End Size [nt]	Area	% of total Area
18S	1,581	2,031	393.0	25.4
28S	2,615	3,612	717.2	46.4

 © Copyright 2003 - 2015 Agilent Technologies, Inc.

Assay Class: Eukaryote Total RNA Nano
Data Path: C:\...Eukaryote Total RNA Nano_DE54108122_2023-05-03_14-29-24.xad
Created: 5/3/2023 2:29:24 PM
Modified: 5/3/2023 2:52:27 PM

Electropherogram Summary Continued ...

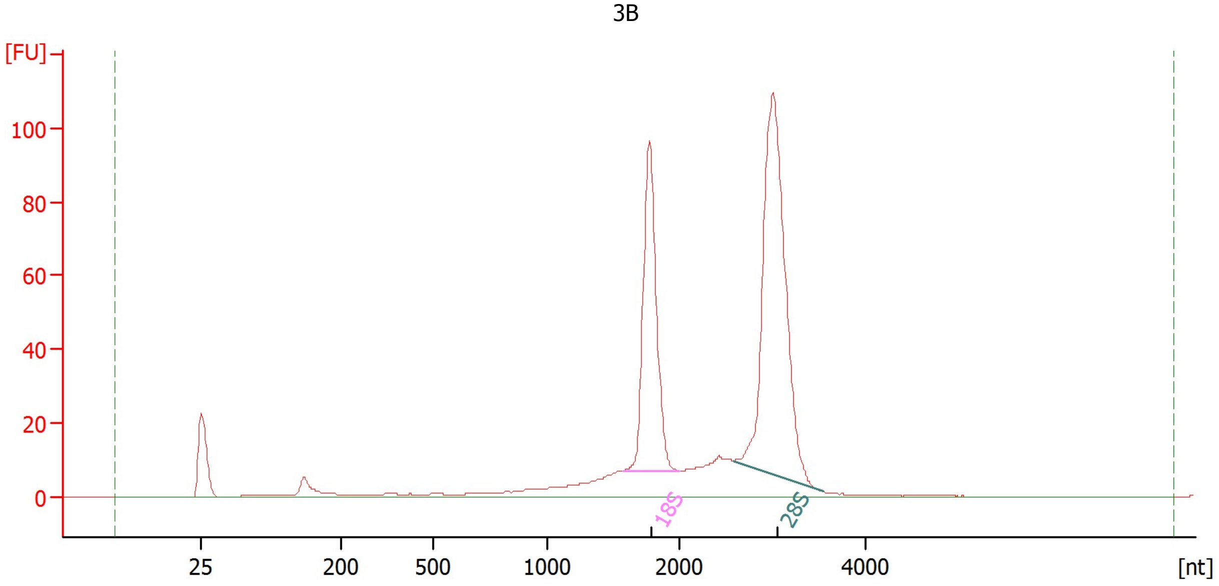

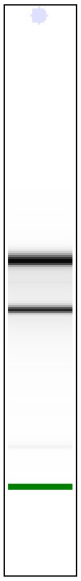

Overall Results for sample 6 : 3B

RNA Area:	613.4	RNA Integrity Number (RIN):	9.7 (B.02.08)
RNA Concentration:	280 ng/µl	Result Flagging Color:	
rRNA Ratio [28s / 18s]:	1.7	Result Flagging Label:	RIN: 9.70

Fragment table for sample 6 : 3B

Name	Start Size [nt]	End Size [nt]	Area	% of total Area
18S	1,571	1,989	142.7	23.3
28S	2,582	3,538	245.3	40.0

© Copyright 2003 - 2015 Agilent Technologies, Inc.

Assay Class: Eukaryote Total RNA Nano Created: 5/3/2023 2:29:24 PM
Data Path: C:\...Eukaryote Total RNA Nano_DE54108122_2023-05-03_14-29-24.xad Modified: 5/3/2023 2:52:27 PM

Electropherogram Summary Continued ...

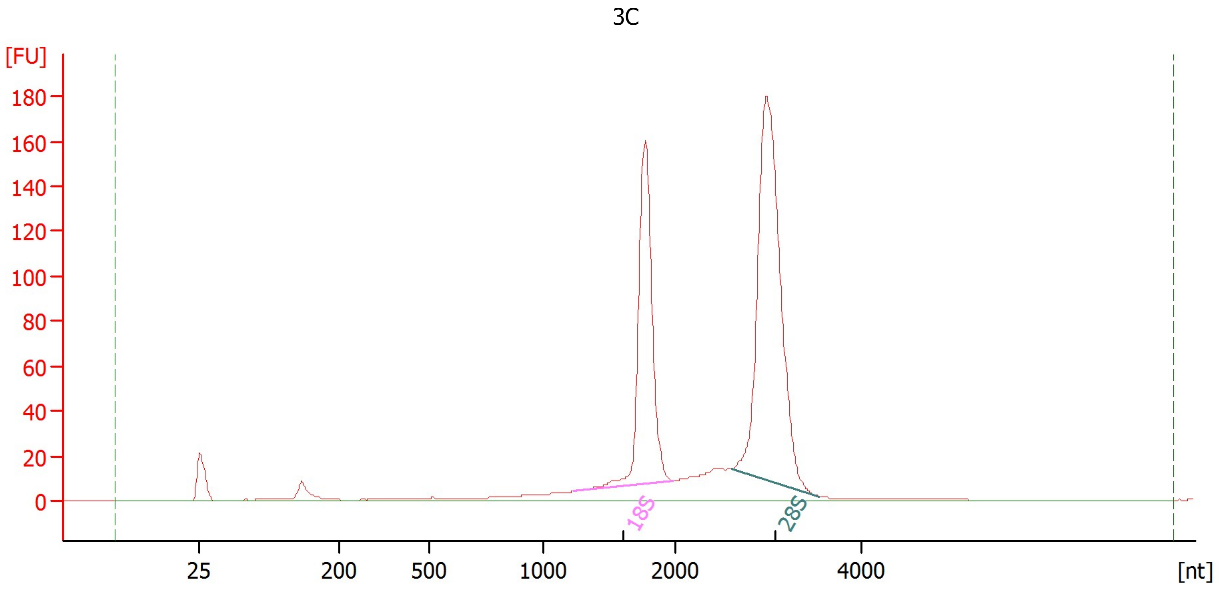

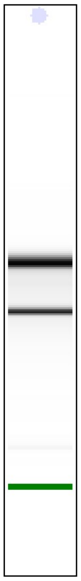

Overall Results for sample 7 : 3C

RNA Area:	952.7	RNA Integrity Number (RIN):	10 (B.02.08)
RNA Concentration:	436 ng/µl	Result Flagging Color:	
rRNA Ratio [28s / 18s]:	1.7	Result Flagging Label:	RIN:10

Fragment table for sample 7 : 3C

Name	Start Size [nt]	End Size [nt]	Area	% of total Area
18S	1,221	1,988	252.1	26.5
28S	2,607	3,543	418.6	43.9

 © Copyright 2003 - 2015 Agilent Technologies, Inc.

Assay Class: Eukaryote Total RNA Nano
Data Path: C:\...Eukaryote Total RNA Nano_DE54108122_2023-05-03_14-29-24.xad
Created: 5/3/2023 2:29:24 PM
Modified: 5/3/2023 2:52:27 PM

Electropherogram Summary Continued ...

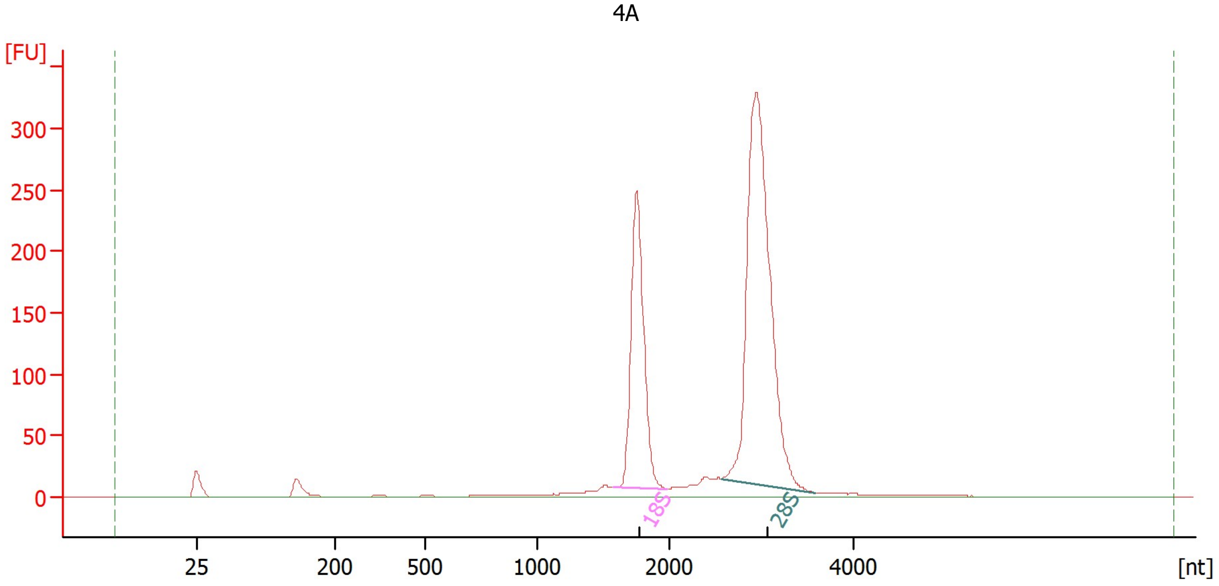

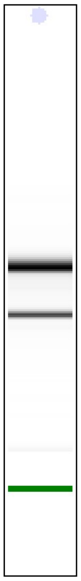

Overall Results for sample 8 : 4A

RNA Area:	1,465.8	RNA Integrity Number (RIN):	10 (B.02.08)
RNA Concentration:	670 ng/µl	Result Flagging Color:	
rRNA Ratio [28s / 18s]:	2.1	Result Flagging Label:	RIN:10

Fragment table for sample 8 : 4A

Name	Start Size [nt]	End Size [nt]	Area	% of total Area
18S	1,572	1,978	380.3	25.9
28S	2,562	3,598	794.5	54.2

 © Copyright 2003 - 2015 Agilent Technologies, Inc.

Assay Class: Eukaryote Total RNA Nano
Data Path: C:\...Eukaryote Total RNA Nano_DE54108122_2023-05-03_14-29-24.xad
Created: 5/3/2023 2:29:24 PM
Modified: 5/3/2023 2:52:27 PM

Electropherogram Summary Continued ...

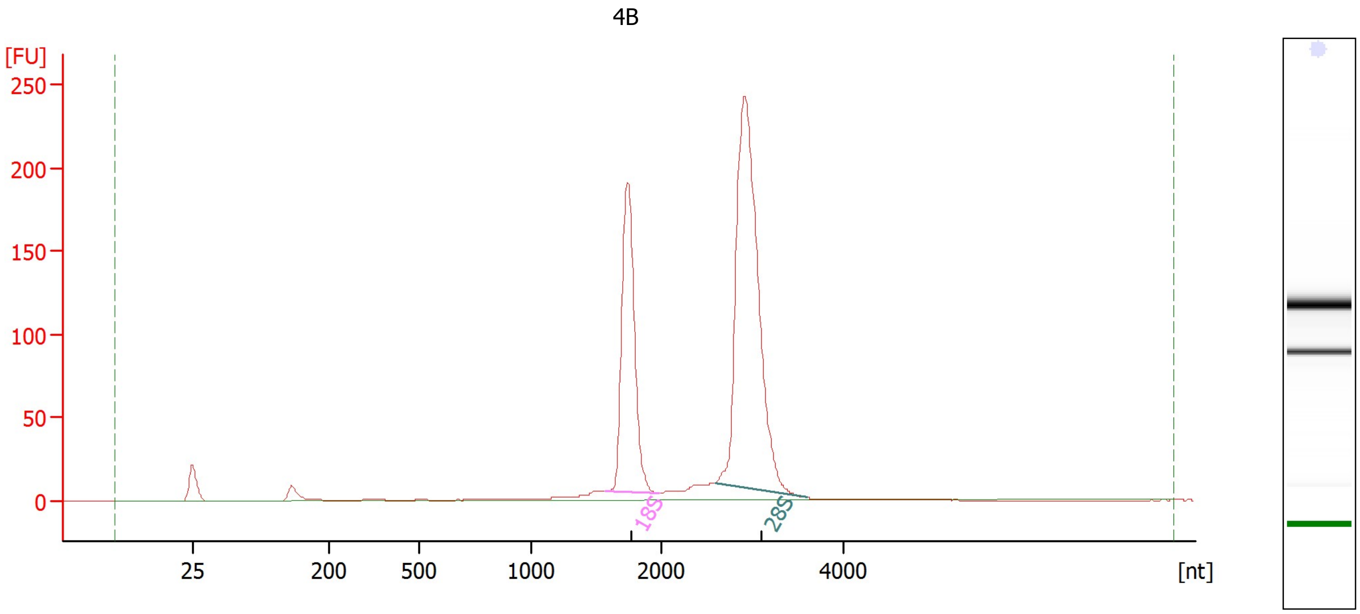

Overall Results for sample 9 : 4B

RNA Area:	1,043.1	RNA Integrity Number (RIN):	10 (B.02.08)
RNA Concentration:	477 ng/µl	Result Flagging Color:	
rRNA Ratio [28s / 18s]:	1.9	Result Flagging Label:	RIN:10

Fragment table for sample 9 : 4B

Name	Start Size [nt]	End Size [nt]	Area	% of total Area
18S	1,567	1,985	290.7	27.9
28S	2,588	3,597	562.3	53.9

 © Copyright 2003 - 2015 Agilent Technologies, Inc.

Assay Class: Eukaryote Total RNA Nano
Data Path: C:\...Eukaryote Total RNA Nano_DE54108122_2023-05-03_14-29-24.xad
Created: 5/3/2023 2:29:24 PM
Modified: 5/3/2023 2:52:27 PM

Electropherogram Summary Continued ...

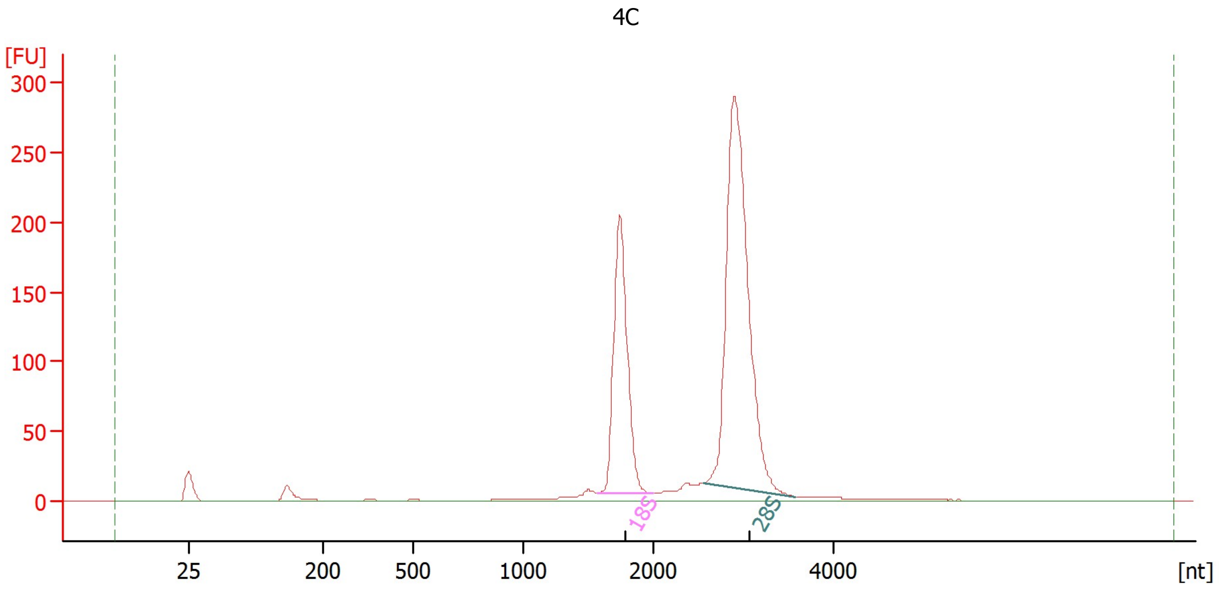

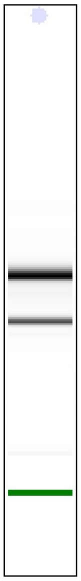

Overall Results for sample 10 : 4C

RNA Area:	1,255.6	RNA Integrity Number (RIN):	10 (B.02.08)
RNA Concentration:	574 ng/µl	Result Flagging Color:	
rRNA Ratio [28s / 18s]:	2.0	Result Flagging Label:	RIN:10

Fragment table for sample 10 : 4C

Name	Start Size [nt]	End Size [nt]	Area	% of total Area
18S	1,570	2,000	332.6	26.5
28S	2,556	3,573	680.3	54.2

Assay Class: Eukaryote Total RNA Nano
Data Path: C:\...Eukaryote Total RNA Nano_DE54108122_2023-05-03_14-29-24.xad
Created: 5/3/2023 2:29:24 PM
Modified: 5/3/2023 2:52:27 PM

Electropherogram Summary Continued ...

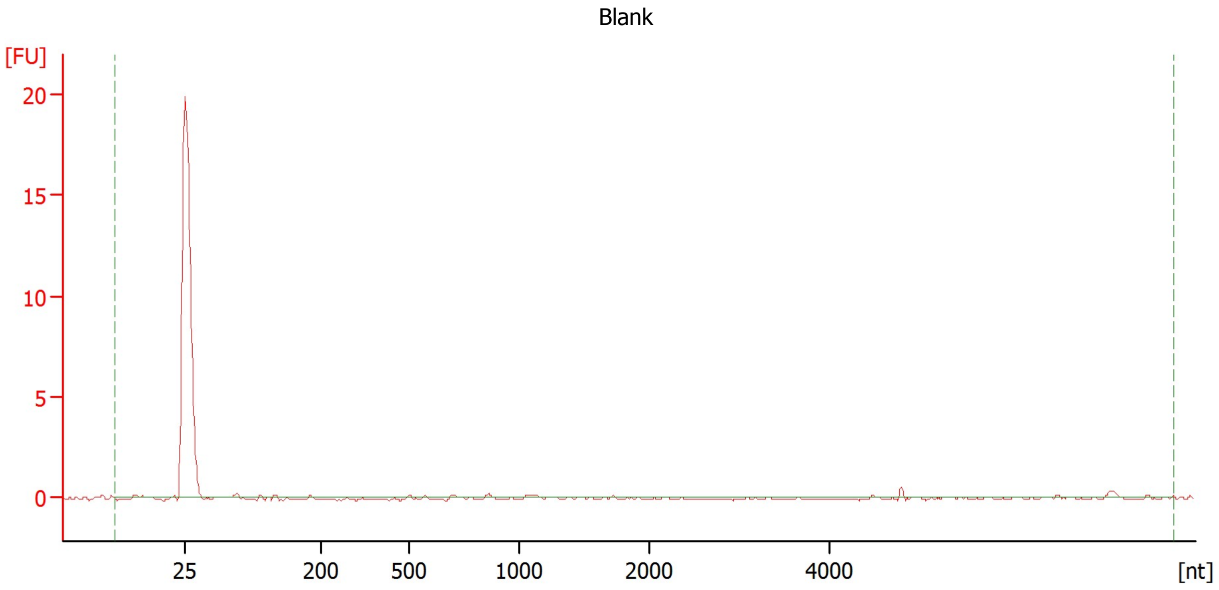

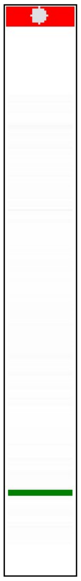

Overall Results for sample 11 : **Blank**

RNA Area:	1.8	RNA Integrity Number (RIN):	N/A (B.02.08)
RNA Concentration:	1 ng/µl	Result Flagging Color:	
rRNA Ratio [28s / 18s]:	0.0	Result Flagging Label:	RIN N/A

 © Copyright 2003 - 2015 Agilent Technologies, Inc.

Assay Class:	Eukaryote Total RNA Nano	Created:	5/3/2023 2:29:24 PM
Data Path:	C:\...Eukaryote Total RNA Nano_DE54108122_2023-05-03_14-29-24.xad	Modified:	5/3/2023 2:52:27 PM

Electropherogram Summary Continued ...

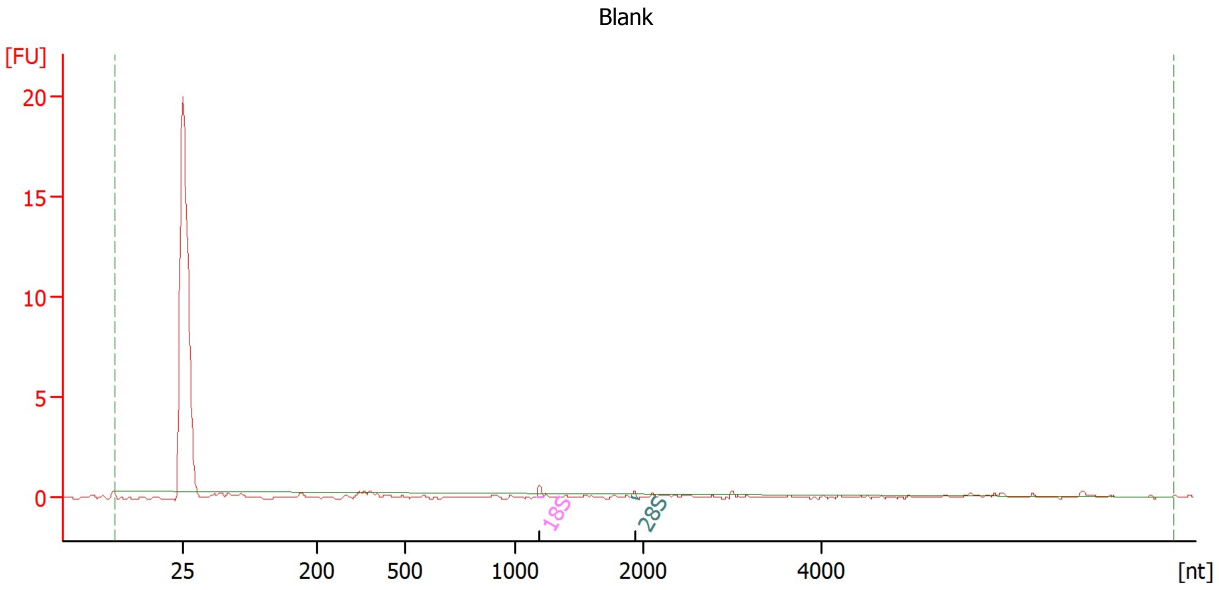

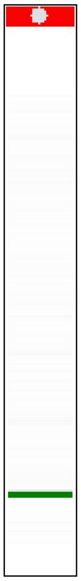

Overall Results for sample 12 : Blank

RNA Area:	0.7	RNA Integrity Number (RIN):	N/A (B.02.08)
RNA Concentration:	0 ng/µl	Result Flagging Color:	
rRNA Ratio [28s / 18s]:	0.5	Result Flagging Label:	RIN N/A

Fragment table for sample 12 : Blank

Name	Start Size [nt]	End Size [nt]	Area	% of total Area
18S	1,170	1,229	0.3	44.6
28S	1,905	1,972	0.2	23.8

 © Copyright 2003 - 2015 Agilent Technologies, Inc.

Assay Class:	Eukaryote Total RNA Nano	Created:	5/3/2023 2:29:24 PM
Data Path:	C:\...Eukaryote Total RNA Nano_DE54108122_2023-05-03_14-29-24.xad	Modified:	5/3/2023 2:52:27 PM

Gel Image

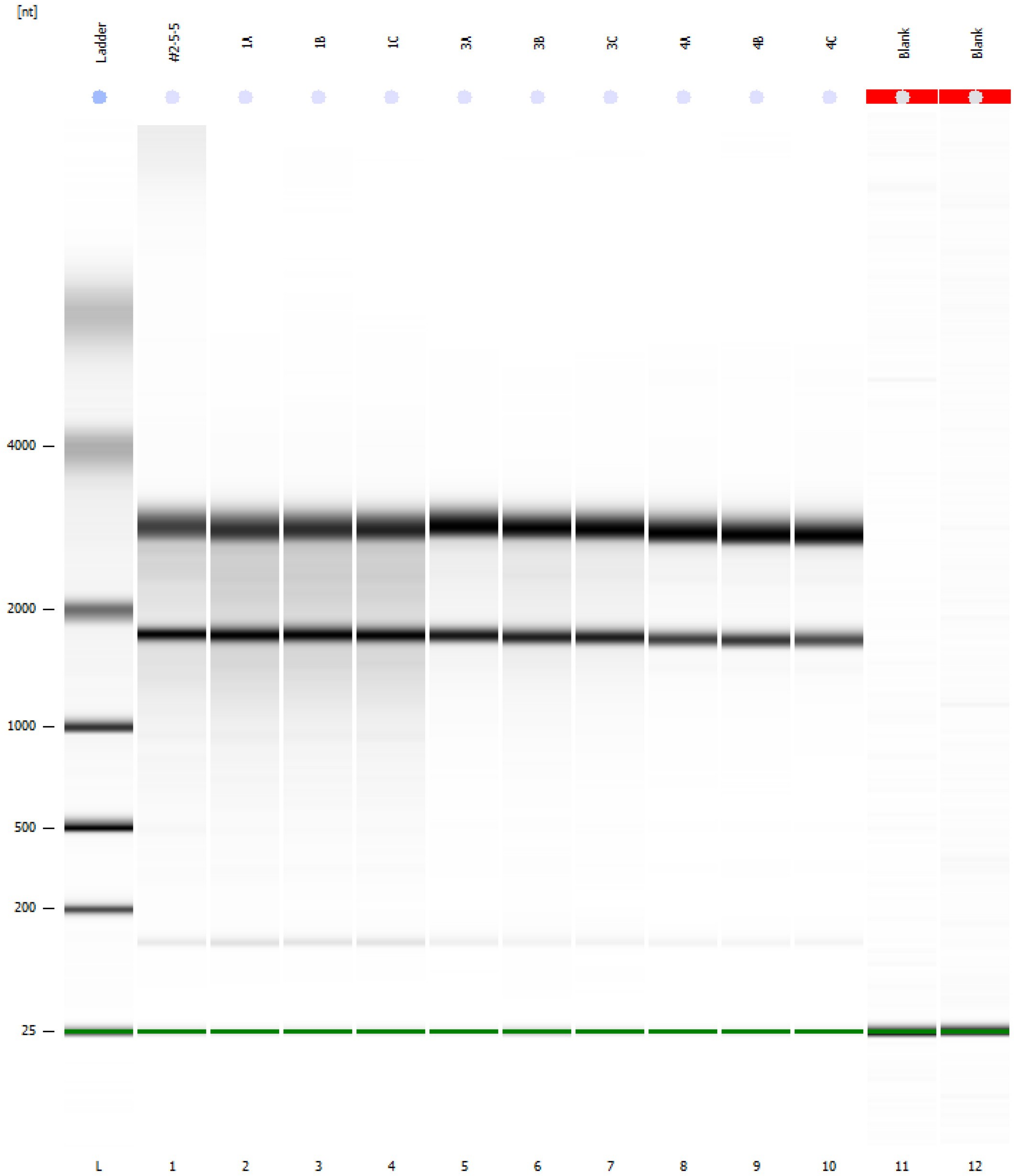

 © Copyright 2003 - 2015 Agilent Technologies, Inc.
