# Supplementary material for: CatSper mediates not only chemotactic behavior but also the motility of ascidian sperm
Source: Front Cell Dev Biol. 2023 Nov 2;11:1136537. doi: 10.3389/fcell.2023.1136537 (PMC10652287; doi:10.3389/fcell.2023.1136537)
Supplement: Supplementary file 1 [file DataSheet1.zip › Supplementary_text.docx]

Supplementary Material

CatSper mediates not only chemotactic behavior but also motility of ascidian sperm

Taiga Kijima^1^, Daisuke Kurokawa^1^, Yasunori Sasakura^2^, Michio Ogasawara^3^, Satoe Aratake^1^, Kaoru Yoshida^4^, and Manabu Yoshida^1^*

*** Correspondence:** Manabu Yoshida: [yoshida@mmbs.s.u-tokyo.ac.jp](mailto:yoshida@mmbs.s.u-tokyo.ac.jp)

# Supplementary Data

## Materials and methods for Supplemental figure

### Reverse transcriptase polymerase chain reaction (RT-PCR)

The predicted *Catsper* genes were identified from the genome database of *C. intestinalis* ( <http://ghost.zool.kyoto-u.ac.jp/cgi-bin/gb2/gbrowse/kh/>). Total RNA was extracted from each specimen using the RNeasy Mini Kit (Qiagen, Hilden, Germany), and the RNA concentration was determined by measuring the absorbance at 260 nm relative to the absorbance at 280 nm. One microgram of total RNA was reverse -transcribed to cDNA using a Transcriptor First Strand cDNA Synthesis Kit (Roche Diagnostics) with an anchored-oligo(dT)_18_ primer and a random hexamer primer. The 50-µl reaction mixture contained 32 µl nuclease-free water, 1.0 µl cDNA, 1.5 µl (10 µmol/l) each primer, 5 µl (2 mM) dNTPs, 3 µl (25 mM) MgSO_4_, 5 µl (10x) KOD plus ver.2 buffer, and 1 µl KOD plus (TOYOBO, Osaka, Japan). The primers used for detecting *Catsper1*:5′-TCA CAC AGC ATG GAC GGT AA-3′ and 5′-GCA TCA GGA TTG CTC CTA AAG A-3′; *Catsper2*:5′-CGC CCA AGT ATC ATC CTG AA-3′ and 5′-GAC ATG TAC TTA ATA GTG GCA ACA CC-3′; *Catsper3*:5′-CGA AAG CGA AGT TTT TAA TGG TCT-3′ and 5′-GCACTTCCTTATAAGCCATGTGTAA-3′ ; *Catsper4*:5′-AGG AAG AAC AGC GGT CGA AG-3′ and 5′-CTG AGG CTT CAG AGC ATC CA-3′; the household gene glyceraldehyde-3-phosphate dehydrogenase (*Gapdh*): 5′-ACC CAG AAG ACA GTG GAT GG- 3′ and 5′ -CAG GAC ACC AGC TTC ACA AA -3′. The amplification cycle started with denaturation at 94°C for 2 min, followed by 30 cycles of denaturation at 98°C for 10 s, annealing at 55°C for 30 s, and 68°C for 1 min. PCR products were analyzed on 2% (wt/vol) agarose gels stained with 0.5 µg/mL ethidium bromide and were visualized under UV light. Images of the gels were captured using a gel imaging device (Printgraph, AE-6932; ATTO, Tokyo, Japan), and the acquired images were processed using Adobe Photoshop and Adobe Illustrator (Adobe Systems, San Jose, CA, USA).

# Supplementary Table. Figures, and Videos

## Supplementary Table

**Supplementary Table S1:** Primers used for PCR experiments to detect ascidian *Catsper* genes.

| Gene (Gene model ID*) | set # | Primer sequence (5’> 3’) |
| --- | --- | --- |
| *Catsper1* (KY21.Chr10.327) | #1 fwd | GATCACTTGCCTCCATTCGTTTC |
|  | #1 Rev | CGATGGACACGAAGAGACCTC |
|  | #2 fwd | TGGATAATTTTCAACTTACGCTGCG |
|  | #2 Rev | TGCTGCCAAGTTATCGTCGG |
| *Catsper2* (KY21.Chr10.1075) | #1 fwd | GGAAGAACGGATGGAATGAATTGG |
|  | #1 Rev | GACCATTCCCGATCCACTAGC |
|  | #2 fwd | CTCATGACGTTGGACCACTGG |
|  | #2 Rev | AAACGACCCAACCCATATCCATG |
| *Catsper3* (KY21.Chr2.259) | #1 fwd | GGATCTTGGTTCTGCATTTCTCAC |
|  | #1 Rev | TATGGTGTAAATTCGTGAGGAGGG |
|  | #2 fwd | CGAACTCGCGCAGAAGATTAGC |
|  | #2 Rev | CAATGTCTGCTGCTGGAAAGTAAGG |
| *Catsper4* (KY21.Chr2.677) | #1 fwd | ACCTGATCATGGGCATCTTCATATG |
|  | #1 Rev | GTGTTACTGAAGAAAGCAAGCCAC |
|  | #2 fwd | GGTTCTGCTTTGAATCACAGGAAG |
|  | #2 Rev | TTGAAACAGATGCCGCAATGTC |
| *Gapdh* (KY21.Chr11.276) | fwd | ACC CAG AAG ACA GTG GAT GG |
|  | Rev | CAG GAC ACC AGC TTC ACA AA |

*Gene model ID is the number shown in the *C. intestinalis* type A genome database (Ghost: <http://ghost.zool.kyoto-u.ac.jp/default_ht.html>). ([Satou, Tokuoka, Oda-Ishii, Tokuhiro, Ishida, Liu, and Iwamura, 2022](#_ENREF_35))

## Supplementary Figures

**Supplementary Figure S1.** Expression of *Catsper* genes in the ascidian *Ciona intestinalis* in each tissue of mature adults (A) and developing juveniles (B). The household gene glyceraldehyde-3-phosphate dehydrogenase (*Gapdh*) was used as a control. Gene expression was examined using RT-PCR. Arrowheads indicate the bands of RT-PCR products.

**(other file)**

**Supplementary Figure S2.** Quality check results of RNA collected for qPCR measurements using a bioanalyzer.

**(other file)**

**Supplementary Figure S3.** Raw data of qPCR analysis shown in Figure 1.

**Supplementary Figure S4.** Another typical result of western blotting with the anti-CatSper3 antibody of sperm and testes from a *Catsper3* KO animal (right). The ascidian CatSper3 protein shows a 50 kDa band (arrowhead). As a negative control, we performed an experiment without the anti-CatSper3 primary antibody. Total electrophoresed proteins are shown after Coomassie Brilliant Blue R staining (right).

## Supplementary Videos

**Supplementary Movie S1.** Behavior of the wildtype sperm around the capillary containing 1 µM SAAF.

**Supplementary Movie S2.** Behavior of the *Catsper3* KO sperm around the capillary containing 1 µM SAAF.
