# Supplementary figures and images for: CatSper mediates not only chemotactic behavior but also the motility of ascidian sperm
Source: Front Cell Dev Biol. 2023 Nov 2;11:1136537. doi: 10.3389/fcell.2023.1136537 (PMC10652287; doi:10.3389/fcell.2023.1136537)

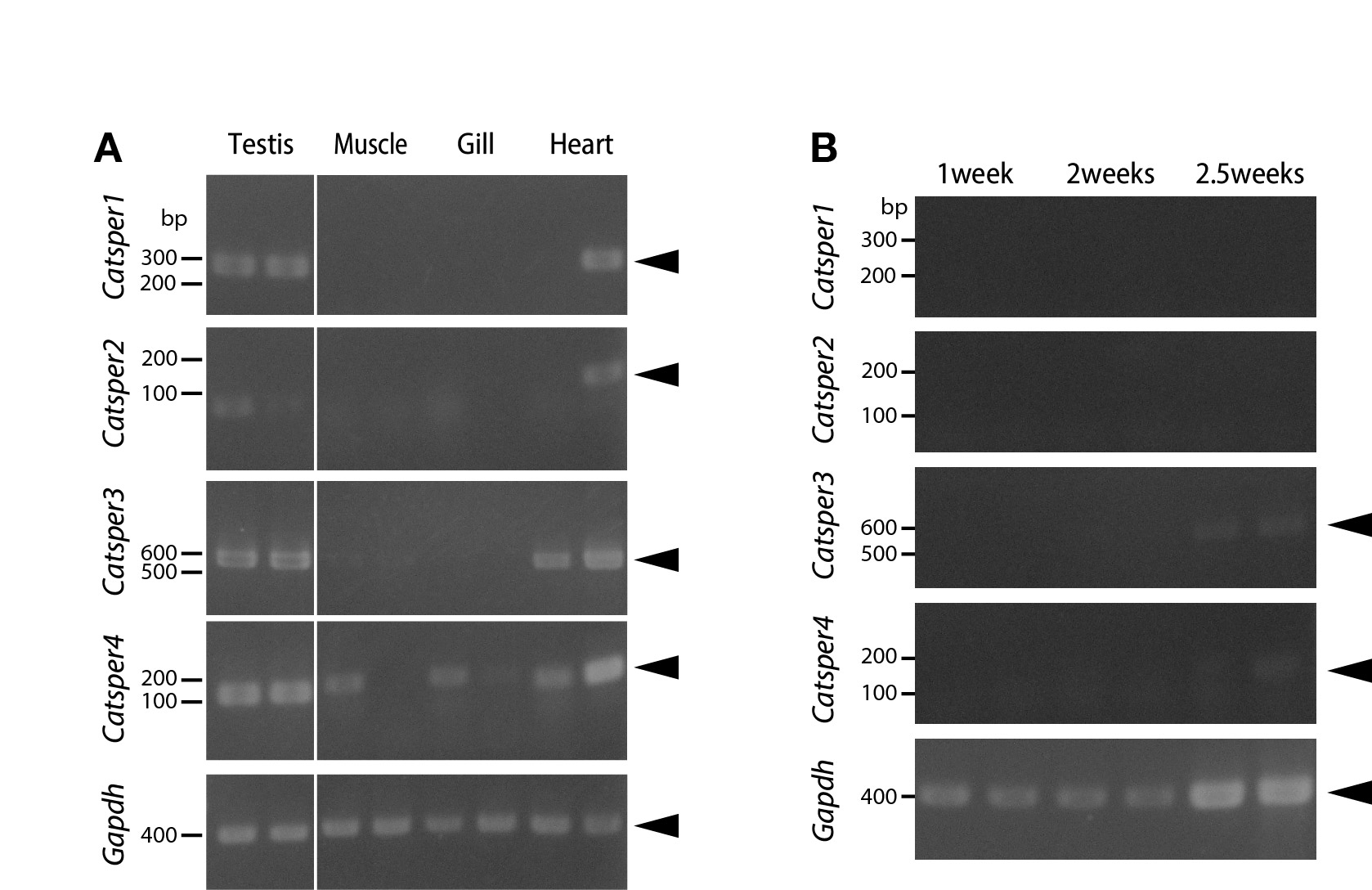

Supplement: Supplementary file 1 [file DataSheet1.zip › Fig_S1.jpg]

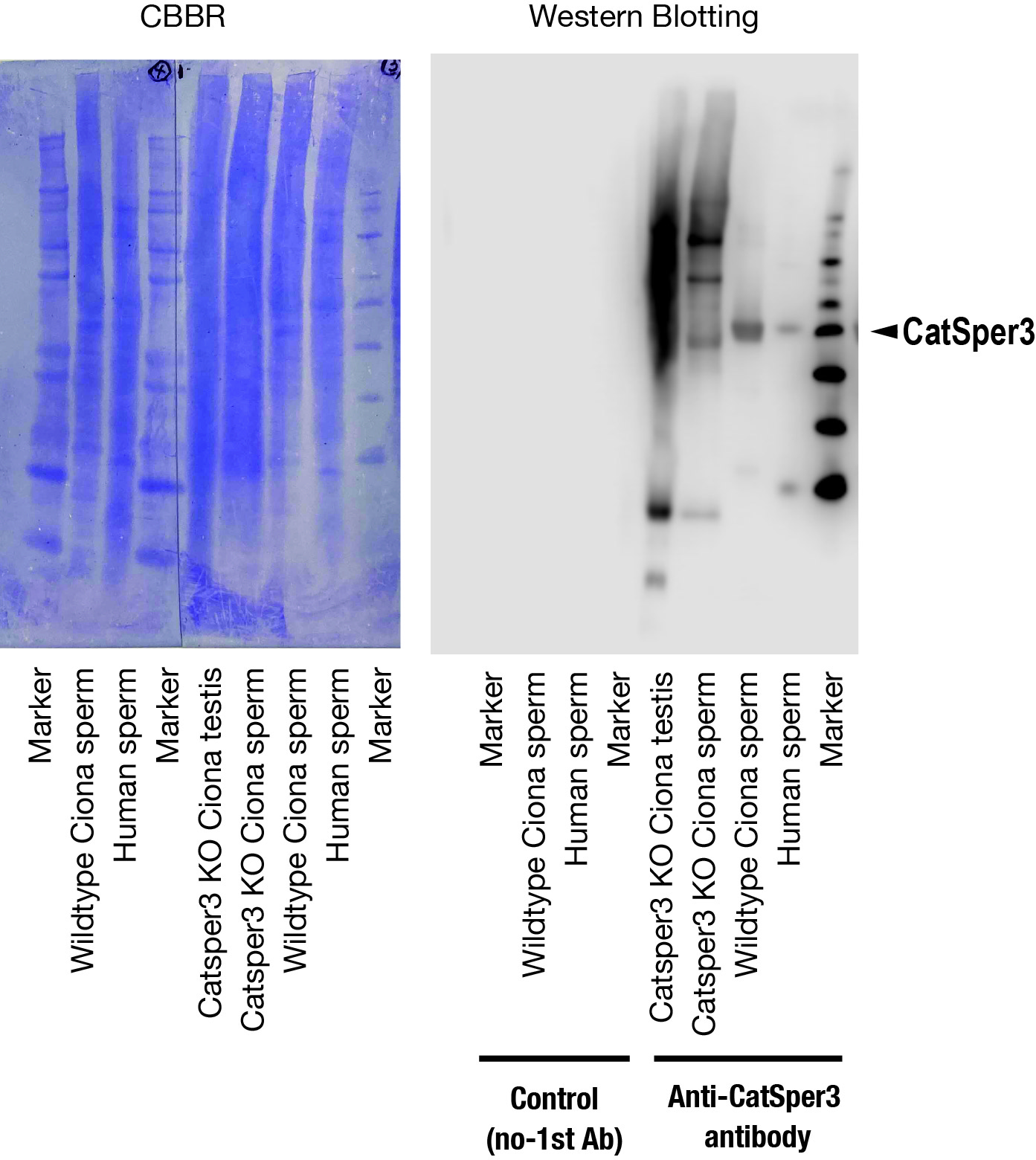

Supplement: Supplementary file 1 [file DataSheet1.zip › Fig_S4.jpg]
